# AlphaFlow: autonomous discovery and optimization of multi-step chemistry using a self-driven fluidic lab guided by reinforcement learning

**DOI:** 10.1038/s41467-023-37139-y

**Published:** 2023-03-14

**Authors:** Amanda A. Volk, Robert W. Epps, Daniel T. Yonemoto, Benjamin S. Masters, Felix N. Castellano, Kristofer G. Reyes, Milad Abolhasani

**Affiliations:** 1 Department of Chemical and Biomolecular Engineering, North Carolina State University, 911 Partners Way, Raleigh, NC 27695-7905 USA; 2 Department of Chemistry, North Carolina State University, Raleigh, NC 27695-8204 USA; 3 Department of Materials Design and Innovation, University at Buffalo, Buffalo, NY 14260 USA

**Keywords:** Optical materials, Chemical engineering, Nanoparticles, Design, synthesis and processing

## Abstract

Closed-loop, autonomous experimentation enables accelerated and material-efficient exploration of large reaction spaces without the need for user intervention. However, autonomous exploration of advanced materials with complex, multi-step processes and data sparse environments remains a challenge. In this work, we present AlphaFlow, a self-driven fluidic lab capable of autonomous discovery of complex multi-step chemistries. AlphaFlow uses reinforcement learning integrated with a modular microdroplet reactor capable of performing reaction steps with variable sequence, phase separation, washing, and continuous in-situ spectral monitoring. To demonstrate the power of reinforcement learning toward high dimensionality multi-step chemistries, we use AlphaFlow to discover and optimize synthetic routes for shell-growth of core-shell semiconductor nanoparticles, inspired by colloidal atomic layer deposition (cALD). Without prior knowledge of conventional cALD parameters, AlphaFlow successfully identified and optimized a novel multi-step reaction route, with up to 40 parameters, that outperformed conventional sequences. Through this work, we demonstrate the capabilities of closed-loop, reinforcement learning-guided systems in exploring and solving challenges in multi-step nanoparticle syntheses, while relying solely on in-house generated data from a miniaturized microfluidic platform. Further application of AlphaFlow in multi-step chemistries beyond cALD can lead to accelerated fundamental knowledge generation as well as synthetic route discoveries and optimization.

## Abstract (web summary)

Autonomous exploration of materials design space is hindered by its high dimensionality and the scarcity of data. In this work, we present AlphaFlow, a self-driven lab guided by reinforcement learning that enables accelerated discovery and optimization of multi-step chemistries.

## Introduction

Integration of machine learning (ML) with automated experimentation techniques in chemistry and materials science have heralded the arrival of new research strategies, i.e., self-driving labs (SDLs), capable of exploring chemistry and materials science problems with unparalleled speed and efficiency^1–7^. These SDLs are composed of the automated physical (experiment conduction) and digital (data processing and algorithm-guided experiment selection) steps. While proof-of-concept SDLs have been realized to an extent for several examples, including robotics-integrated lab spaces and microfluidic reaction systems^8–12^, truly self-guided, exploratory autonomous research is still limited to applications with well-studied, constrained parameter spaces. For SDLs in chemistry and materials science to reach widespread adoption, technologies must overcome two main barriers when dealing with complex multi-stage chemistries: dimensionality and data scarcity. “The curse of dimensionality” is a common term in data science that is used to describe the exponential increase in a parameter space size as the dimensionality of a problem increases^13^. This issue is prominent in multi-step decision-making processes, including multi-step syntheses, ubiquitous in chemistry and materials science, which exhibit large parameter space complexity after only a few decision steps.

Precision synthesis of heterostructure quantum dots (QDs) using the colloidal atomic layer deposition (cALD) technique is an exemplary multi-stage chemistry with a high-dimensional experimental space. Conventional cALD involves the sequential injection, removal, and washing of reactants and stabilizing ligands to grow hetero-nanostructures in a room temperature, controlled, layer-by-layer manner. Compared to other shelling techniques that have been studied in automated reactors^14–17^, the self-limiting, monolayer precision of cALD makes it a promising strategy to synthesize hetero-nanostructures with tuned confinement regimes and nanometer scale heterostructure layers^18^. In addition to control over luminescent and electronic properties, the self-limiting potential of cALD can preserve the size dispersity of starting QDs. Beyond applications to metal-chalcogenide QDs, since cALD is a room temperature synthesis technique, it may be applied to more temperature-sensitive materials, such as metal halide perovskite QDs.

**Fig. 1:**
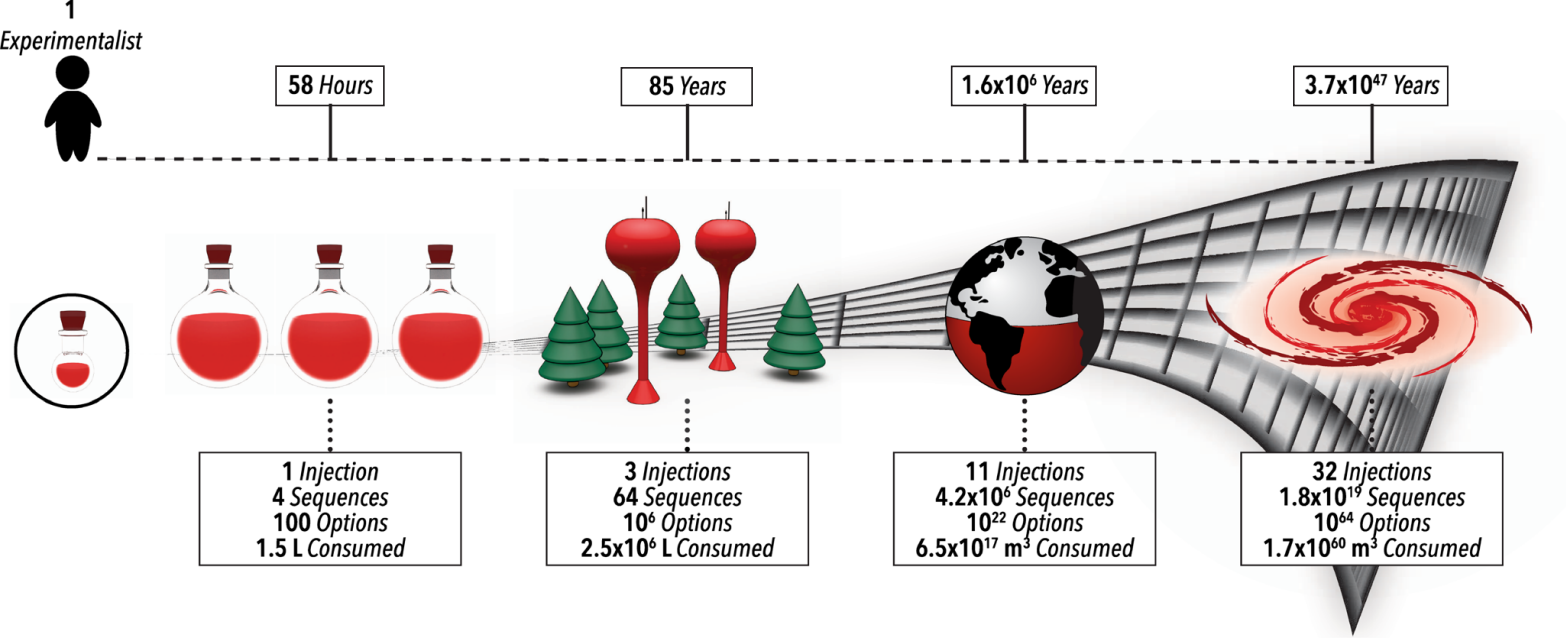
Curse of dimensionality in multi-step chemistry. Illustration of the exponentially increasing complexity and required resources for a batch multi-step synthesis consisting of four possible step choices, up to 32 sequential steps. Reaction option estimates are based on four possible reagents, five possible volumes, and five possible reaction times. Volume estimates are based on 10 ml starting material and an additional 5 ml of reagent per step. Time estimates include preparation and sampling time and are based on 30 min to start each experiment and 5 min per addition of a reagent.

In cALD chemistry, with each sequence step (either a new surface reaction, ligand addition, or wash step), the parameter space of cALD grows exponentially (Fig. 1). Likewise, the time and material cost of conventional parameter space exploration grows exponentially. In addition to expanding dimensionality, each cALD cycle requires precise control over reaction sequence, relative concentrations, and reaction time, as many reaction pathways can happen in parallel depending on these parameters. For example, it was recently shown that beyond colloidal stabilization, oleate ligands are necessary for metal oxide nucleation and growth via single-phase cALD approaches^19^. Such steps can also be nondeterministic. That is, the outcome of an action taken at a given material state, like many syntheses with complex kinetics, can change based on hidden states which are unable to be directly quantified in situ (e.g., the surface coverage of ligands). cALD-based chemistries, because of their expansive parameter space, as well as laborious multi-step and dynamic nature, require new approaches beyond existing SDLs to explore and optimize.

Several prior studies have leveraged SDLs with retrosynthetic planning algorithms to enable on-demand production of user-selected small molecules through elaborate multi-step synthesis routes both using batch^20–24^ and flow reactors^4,5,25–27^. However, these studies rely on the integration of physics-based models with extensive applicable literature data for every individual reaction step. Therefore, the retrosynthetic planning approach is less applicable to many of the challenges posed by under-studied or immeasurable reaction routes. Nanoscience, in particular, presents many reactive systems that are difficult to reproduce from lab-to-lab or reactor-to-reactor, have limited applicable literature data, and possess complex heterogenous structures and reaction intermediates that cannot be conclusively identified. Consequently, many SDL studies involving nanoparticles rely strictly on data generated by one reactor^9,11,28–33^. Therefore, ML techniques which can handle sequence-dependent processes with in-house generated data sets are imperative to solving novel complex multi-step systems.

Reinforcement learning (RL) has recently emerged as a powerful subset of ML, which has the potential to surpass human performance in such dynamic systems^34^. Contrasting with the more commonly applied black-box methods, which seek to identify reaction space behaviors by observing the final outputs that result from a given set of input parameters, RL operates by monitoring the current state of the system and mapping an action to the resulting response from that state. The structure of RL is inherently compatible with long, multi-step processes because, instead of attempting to solve the entire reaction space at once, RL can break down decisions into isolated steps and predict the future effects of those steps.

One notable example of the potential of RL is AlphaGo, the first computer program to defeat a professional Go player in 2016, 20 years after its Chess playing predecessor, Deep Blue^35^. The delay in developing both strategy game programs was due to the inability of older algorithms (used in Deep Blue) to handle the large number of possible moves found in Go. Algorithm-driven chemistry research has reached a similar impasse, where a new approach, beyond traditional supervised learning (SL), is necessary to solve and discover novel materials and molecules with high dimensionality and dynamic syntheses. In addition to using RL-based algorithms, AlphaGo trained itself through many iterations of trial-and-error, thereby creating a data-rich environment without prior knowledge. Algorithm-guided synthesis platforms must also be able to perform trial-and-error exploration to reach the full potential of RL, learn from unforeseen results, and alleviate common data scarcity and reproducibility issues in literature. RL-based strategies have been demonstrated in silico towards process synthesis and synthetic route discovery^36–38^. However, the real-time iterative learning of RL-based approaches makes it a powerful tool that has not yet been integrated with closed-loop experimentation strategies. Miniaturized and automated experimentation strategies have the potential to integrate the trial-and-error aspects of RL with minimal material and time loss upon experiment termination/failure. In addition, these strategies can meet the data generation needs of ML-guided experimentation.

In this work, we introduce AlphaFlow, an RL-guided SDL with modular fluidic processing units which can autonomously generate new chemistry knowledge and identify optimal synthetic routes for high-complexity, multi-step reactions. The multi-step chemistry explored by AlphaFlow is based on cALD reactions for the precision synthesis of hetero-nanostructures^39–41^. With cadmium selenide (CdSe)/cadmium sulfide (CdS) core-shell QDs as a demonstrative hetero-nanostructure, we use AlphaFlow to explore and discover multi-step chemistries that exceed the shell growth capabilities of the conventional cALD chemistry, without any prior knowledge of conventional reagent addition orders or constraints. We show that the developed RL-guided SDL is effective at autonomously navigating the expansive multi-step reaction space. Without any pretraining or any prior knowledge of conventional cALD sequences (i.e., without any domain knowledge of reagent sequences), AlphaFlow successfully identified a new reaction sequence that resulted in nanomaterials with a higher absorption peak wavelength (i.e., higher shell growth) than the conventional sequence route. In addition, AlphaFlow was able to optimize reaction conditions to improve nanomaterial quality for the discovered route. AlphaFlow marks the first integration of RL with automated multi-step chemistry. Through this integration, we have developed an SDL, contrasting to cheminformatic and retrosynthetic planning methods, that can autonomously and independently explore, learn, and optimize multi-step reactions with parameter space complexities exceeding 40 dimensions. In this way, the developed SDL demonstrates strictly algorithm-driven discovery of high-level concepts that were previously only accessible through manual time-, labor-, and resource-intensive experimentation as well as human intuition and direction—illustrated in Supplementary Fig. 1. This autonomous experimentation strategy extends and augments the intellectual reach of human researchers by enabling rapid, intelligent, and constant exploration of complex reaction spaces. We expect the further application of AlphaFlow to expand opportunities for lateral innovation through new observations and discoveries that otherwise could not be elucidated in high-dimensionality, dynamic reactions.

## Results

### SDL hardware: modular fluidic micro-processors

**Fig. 2:**
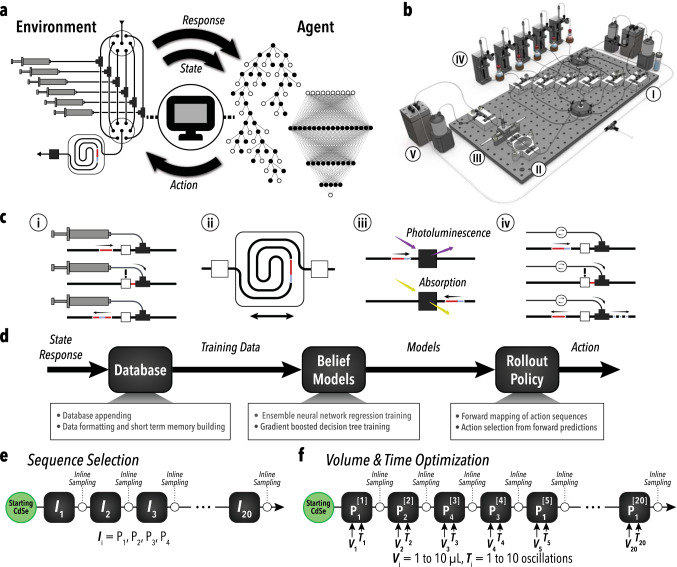
Overview of AlphaFlow. **a** Illustration of an RL-based feedback loop between the learning agent and the automated experimental environment. **b** Schematic of full reactor system with (I) reagent injection, (II) droplet oscillation, (III) optical sampling, (IV) phase separation, (V) waste collection, and (VI) refill modules. **c** Schematics of individual module functions corresponding to (i) formulation, (ii) synthesis, (iii) characterization, and (iv) phase separation. **d** General flow diagram of learning agent condition selection process. **e**, **f** Block diagram of the reaction space exploration campaigns, sequence selection, and volume-time optimization, respectively. P_1_, P_2_, P_3_, and P_4_ correspond to an arbitrary set of injection reagents, which for the purpose of this study, are oleylamine, sodium sulfide, cadmium acetate, and formamide, respectively. Sequence selection was performed using constant reagent injection volumes and reaction times and directing the system to select the order that reagents are injected. Volume-time optimization was conducted by using an autonomously learned order of reagent injections, specified by the sequence selection campaign, and setting the system to identify optimal injection volumes and reaction times for each of the twenty steps.

The developed SDL, shown in Fig. 2a, operates from a starting position of no prior information on the reaction sequence, then rapidly generates data on a multi-step process by leveraging RL and a high-efficiency microdroplet flow reactor. Microscale flow reactors encompass a growing class of reaction systems that leverage the high efficiency and facile automation capabilities of microfluidics to produce novel insights and unique control of reactive processes^42–44^. Prior studies have leveraged microscale flow reactors to achieve large data sets through process automation, high-throughput screening, and closed-loop experimentation^10,11,28^. However, microdroplet-based systems suffer from several drawbacks with respect to the scalability and solid materials handling. A variety of methods can be employed to directly transfer gained knowledge from the single microdroplet system towards larger scale systems, including non-fouling continuous flow formats for biphasic reactions^45^. Further development in these areas is required for broader application and adoption of microscale flow reactors within SDLs.

The multi-step chemistry studies of AlphaFlow presented here were facilitated by the versatility and data generation efficiency of the single microdroplet system and developed modular fluidic micro-processors. It should be noted that compared to the in situ techniques used in this work, destructive characterization techniques, which would require a new experiment to be started after each characterization, would likely have a slower throughput and less efficient data generation. Although such techniques have been used successfully in other ML-guided SDLs^46^, they may be less amenable to the data requirements of RL-guided experimentation. To mitigate this issue, it is possible to generate more than one reactive microdroplet to increase data generation throughput.

As shown in Fig. 2b, the hardware of the SDL presented in this work, utilizes a single microdroplet format (10 μL). The platform features four integrated modules: (i) formulation, (ii) synthesis, (iii) in situ characterization, and (iv) in-line phase separation. The formulation is carried out by transferring the reactive droplet into an isolated reagent injection channel, where an optical sensor is used to position the microdroplet in a junction for the on-demand addition of the desired reagent. Similar to the oscillatory configuration presented in prior studies, the synthesis micro-processor oscillates the single microdroplet to achieve mixing and repeated spectra analysis^47–49^. In situ optical sampling is conducted automatically at the end of each oscillation, rapidly generating data sets of extracted spectral characteristics using non-invasive measurements, including first absorption peak wavelength (λ_AP_), first absorption peak intensity (λ_PI_), absorption peak-to-valley ratio (*R*_PV_), and photoluminescence peak intensity (*I*_PL_). The phase separation fluidic module segments off the immiscible reagent addition phases using a precisely timed nitrogen (N_2_) injection into the moving microdroplet. Additionally, the SDL is equipped with an automatic reactor washing protocol and reagent syringe refilling mechanism to enable uninterrupted operation over an extended period of time (over 1000 h, depending on precursor stability). The syringe refilling mechanism operates by connecting the syringe to a precursor vial, pressurized under an inert atmosphere, via a selector valve detailed in Supplementary Fig. 3.

AlphaFlow’s hardware and software were built, from the ground up, to be flexible toward system modifications and reaction exploration. Experiments were controlled through a stepwise recipe system, discussed in more detail in Supplementary Note 1 and Supplementary Table 1, where a list of function blocks with specified parameters can be executed in series to conduct larger, more complex multi-step processes. Module development (shown in Supplementary Fig. 2) is then streamlined, as new functions can be developed in isolation before incorporation into the larger system. The full function operation protocols are detailed in Supplementary Figs. 3–9 and Supplementary Movie 1. Furthermore, steps within each of the functions are executed by generalizable action blocks, which call from a library of hardware communication drivers to send commands to a large variety of equipment. The user can individually select the specific make and model of the equipment used in the primary control software and seamlessly change out system components with different hardware modules. This modular approach of AlphaFlow creates a versatile and simple-to-use experimental platform for non-expert researchers. Coupling these traits with the low-cost, accessible tubing-based design of the fluidic micro-processors enables the realization of many of the early promises of plug-and-play, droplet-based microreactors.

Relative to the larger field of flow chemistry, the two primary developments in the module designs of AlphaFlow, which have enabled the exploration of multi-step, multi-phase chemistries, are the isolated reagent injection network (formulation module) and the in-line phase separation module. The reagent injection network is composed of a series of valves, pressurization lines, fluidic connections, and syringe pumps. In brief, AlphaFlow can automatically direct the reactive microdroplet along any of the *n* parallel channels, where the desired reagent can then be injected directly into the microdroplet (*n* = 6 in this study). This configuration resolves one of the fundamental limitations of prior single microdroplet reactor designs in that reagents may be injected in any sequence and with any time delay between injections, without contaminating the droplet with undesired reagents—as shown in Supplementary Fig. 10. This approach makes single microdroplet reactors functionally more valuable tools across a much larger range of reactive systems for SDLs and has enabled the variation in reagent addition sequences studied within this work. Furthermore, this strategy allows for the facile addition of extra injection lines or alternate reaction step modules in isolated sections of the reactor.

In addition to flexible reagents and solvent addition, many multi-step chemistries require the removal of an immiscible phase from the primary reactive solution. In the cALD chemistry studied in this work, the reagent addition and washing steps are carried out through the repeated injection and removal of a polar phase (formamide), with the nanoparticles in the nonpolar (toluene) phase. The phase separation module introduced in this work enables facile phase separation of immiscible fluids with a reusable design. Prior in-line separation methods involved the use of a permeable membrane channel^50,51^. However, these methods are designed for continuous flow systems and are, therefore, difficult to implement in a single microdroplet microreactor due to the propensity for droplet breakup and loss in the permeable channel. Furthermore, the use of colloidal nanoparticles imposes the risk of membrane clogging, which is largely circumvented through the phase separator design. In short summary of the separation system, the central control software calculates the current polar phase microdroplet length using the microdroplet transit times and spectral data. The microdroplet is then driven forward into a separation tee where argon segments off the formamide phase using a specific timing calculated from phase separation calibration curves (Supplementary Fig. 11). This method allows for the precise and reproducible separation and addition of an immiscible phase into the reactive droplet for over 50 consecutive steps. While the in-line phase separation is being carried out, the ML algorithm processes all the current data and either select the next step in the reaction or terminates the experiment due to non-viable reaction conditions. For syntheses which require higher temperatures, depending on the phase compositions of the reactive droplet, the reaction can be carried out in the single microdroplet reactor of AlphaFlow using a heating block housing the tube-based reactor with a carrier gas (up to 220 °C).

### SDL software: RL-guided multi-step synthesis

Shown in Fig. 2c, the developed single microdroplet reactor functions as the environment in the RL algorithm, termed the agent, is interacting with. The RL agent evaluates the state and response from the reactor, given a prior state and action, and decides the next best action to navigate through a high-dimensional space intelligently and efficiently. In these models, the state is represented through a short-term memory (STM) containing the four prior injection conditions. This state definition is designed to account for the expected relevant hidden parameters in the reaction space, which assumes that only the last four injections impact the current decision. While this assumption is unlikely to apply to every achievable state, it is assumed to be a sufficient heuristic for experiment selection. It should be noted that extensions of this parameter could lead to different exploration outcomes and a larger data requirement for the RL algorithm. The response is then represented in the form of a reward based on the in situ measured characteristics of the product (i.e., spectra of the hetero-nanostructures). The agent contains a belief model composed of an ensemble neural network regressor (ENN) that predicts the reward for a given state and action, and a gradient-boosted decision tree that classifies the state-action pairs as either viable or unviable. The belief model of the agent is constantly retrained on new information (new experimental data from the environment) to update its understanding. After retraining, the agent uses a model-based rollout policy to predict the outcome/reward of forward-mapped sequences and, using a decision policy, decides the best next action take.

Selecting a reward function in RL systems is critical for the agent to work towards the correct desired objective. In this study, there are multiple optimization target parameters—λ_AP_, *R*_PV_, and *I*_PL_. A widely acceptable multi-objective optimization strategy is the use of an objective function composed of the weighted sum of individual objectives. However, using this form of reward in a multi-step system can result in undesirable material properties. For example, some cALD reaction conditions, such as the injection of cationic and anionic reagents without washing the product, can induce a large increase in λ_AP_, but the final QD has a significantly lower *R*_PV_ than what can be achieved at that same λ_AP_ through slower reaction steps. As shown in Supplementary Fig. 12A–D, this large increase in λ_AP_ can result in a higher weighted sum reward despite inflicting considerable damage to the nanoparticles and ultimate product quality. For this reason, we have designed the reward to be based on the trajectory of the material properties in the output parameter space, represented by the slope of a local reward metric as a function of λ_AP_. However, applying only a slope reward on the weighted mean reward (local reward) and λ_AP_ alone can also result in undesirable outcomes. Some experimental conditions, such as the initial injection of oleylamine, result in a reduction in both the weighted mean reward (local reward) and λ_AP_. As demonstrated in Supplementary Fig. 12E, F, this combined reduction in quality increases the slope reward metric at later injection steps, despite negatively impacting final material properties. Therefore, the trajectory reward calculation has been modified to only consider improvements in the local reward and to treat all changes in λ_AP_ as positive. The reward, *r*, is then the fitted slope of the local reward improvement (defined by only positive increases in a weighted sum of λ_AP_, *R*_PV_, and *I*_PL_) as a function of the absolute value of the change in λ_AP_ plus λ_AP_, within an eight-point moving window (the slope of the improvement). This application of reward trajectory allows for the agent to favor synthetic routes that retain high *R*_PV_, and *I*_PL_ while maintaining consistent increases in λ_AP._

The viability classifier used in AlphaFlow provides a predicted probability that a state-action pair will result in a terminal condition. Terminal conditions in this work encompass numerous situations that can arise when working with data collection in real-world experimentation. Specifically, terminal conditions include metrics that represent irrecoverable experiments as well as erroneous data. As an example, in this cALD case study, the reward is based heavily on the ability to detect the first absorption peak of the hetero-nanostructures in the reactive droplet. However, if the balance of anionic reagent and stabilizing ligands is incorrect, the nanoparticles can become colloidally unstable and transfer into the formamide phase. This scenario results in no measurable nanoparticle features in the reactive droplet, and that experiment will be terminated. In short, terminal classification can be summarized as (i) there is an undetectable volume of the reactive droplet, (ii) there are no measurable features in the in situ measured UV-Vis absorption spectra, or (iii) there is an insufficient concentration of nanoparticles in the reactive droplet. In these cases, the experiment is automatically terminated, and the droplet is sent to waste collection. The classifier is trained on a binary representation of terminal or non-terminal, and the regressor is trained on a constant penalty value for terminal states, which correspond to experiments with irrecoverably poor outcomes. It should be noted that some fraction of all experiments will have an operational error that results in a failed experiment regardless of the reaction parameters given. In these cases, the experiment is given a false positive terminal classification. While there are inherently self-correcting factors in the RL agent, such as the valuation of uncertainty, that correct these errors, the most effective approach is to prevent failed experiments from occurring. The hardware of AlphaFlow exhibited a low failure rate throughout experimentation (less than 1% of injections) and was able to consistently operate unassisted for multiple days without notable failure.

In the model-based rollout policy, the RL agent of AlphaFlow uses the belief model to predict the outcome/reward of hypothetical future action sequences and decides the next best action to take using a decision policy applied across all predicted action sequences. This forward mapping is conducted by cycling through the model to calculate a predicted reward, given an action and prior state, for each simulated forward action (reagent injection step). The viability probability of each step, predicted by the classifier, is multiplied with prior steps, functioning as a discount factor to discount the likelihood of success (and thus the reward) for steps simulated further in the future and to classify terminal condition sequences when the probability falls below a certain value. Maximum predicted discounted rewards in a forward-mapped sequence are grouped by the first step in the injection sequence, and by applying an upper confidence bound (UCB) decision policy, the standard deviation and mean reward estimates are used to autonomously select and run the next condition.

UCB is a statistical inference policy that balances the exploration of parameter space with the exploitation of a model. In low-dimensional spaces, exploration can often be sufficiently conducted using purely random condition selection^52^, but the multi-step chemistry studied here presents significant challenges due to the high dimensionality and existence of terminal states. For example, in the sequence selection study, when all possible combinations of the first three injection steps were tested, 45% of combinations resulted in terminal conditions. Such a high failure rate can result in considerable experimental costs before sufficient exploration of high-value regions of the parameter space can take place. UCB circumvents this issue by directing the reaction towards known favorable conditions, while simultaneously exploring in regions where the model predicts potential reward.

Using the generalized RL architecture discussed, AlphaFlow was tested in two separate campaigns: (i) autonomous discovery of viable sequences of 20 reagent or solvent additions to efficiently carry out cALD based on understood spectroscopic metrics and reagents—illustrated in Fig. 2d; (ii) self-tuning the reagent injection volume and reaction time at each reagent injection step using the injection sequence discovered in the first campaign—shown in Fig. 2e.

### SDL case study 1: autonomous multi-step synthetic route discovery

In the first case study of AlphaFlow, four injection options were provided for the RL algorithm—oleylamine (OAm), sodium sulfide nonahydrate (Na_2_S*9H_2_O, referred to in this text as Na_2_S), cadmium acetate dihydrate (CdAc_2_*2H_2_O, referred in this text as CdAc_2_), and formamide (FAm)—with constant injection volumes and reaction times for each, selected based on conditions known not to result in terminal values for the first half cycle. Each reaction step was selected in real-time as new data from each experiment iteration was used to inform the decisions of the RL learning agent. The RL agent tested reagent injections by selecting one reagent at a time (using the rollout policy), and then updating the belief model before the next action was chosen. It should be noted that the RL agent was not given any prior domain knowledge of the reaction sequence, which conventionally requires the sequence of OAm-Na_2_S-FAm-FAm-CdAc_2_-FAm-FAm for one full cycle (with phase separation steps between each injection). Furthermore, the RL agent was not explicitly required to carry out any repeated pattern. Because the STM was chosen to not depend on the injection number, the models will favor sequences that are known to produce high rewards, regardless of their injection step number (1–20) (although updates to models occur throughout injection sequences). It should be noted that the reagent compositions were optimized for the conventional literature sequence. This constraint implicitly leverages prior literature knowledge to provide an initial basis for performing experiments and expedite material discovery. Starting with the literature sequence with optimized volumes, times, and compositions also provides a direct comparison between algorithm and human-designed synthetic strategies.

**Fig. 3:**
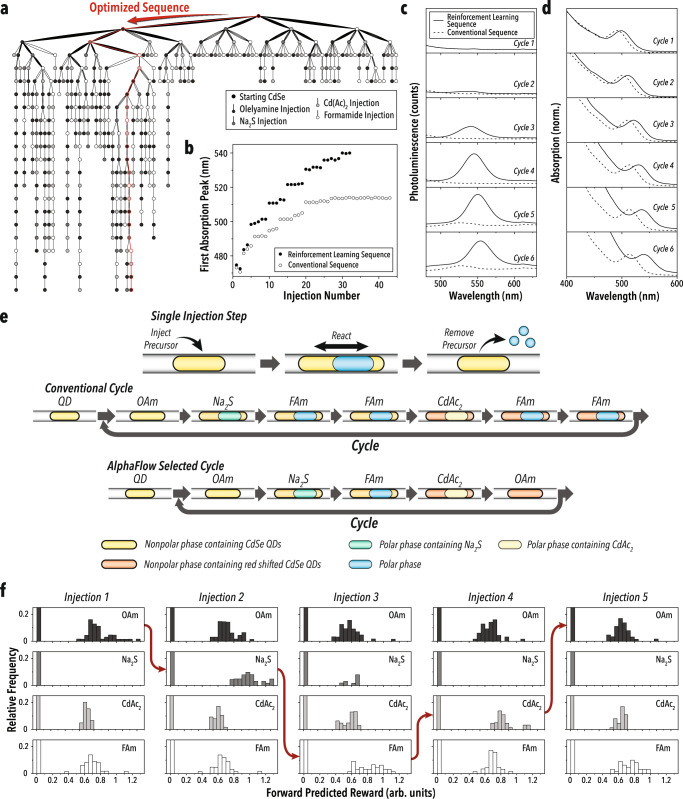
cALD sequence selection campaign results. **a** Illustration of all injection sequences in the exploration runs with the exploited sequence highlighted in red. **b** First absorption peak wavelength of CdSe/CdS hetero-nanostructures as a function of injection number for the RL-selected cALD chemistry and the manually input conventional injection sequence. **c** UV-Vis photoluminescence and absorption (**d**) spectra after each full cALD cycle for the RL-discovered and conventional cALD sequences. **e** Schematic of reagent addition sequences for conventional cALD cycles and the AlphaFlow-selected sequence. **f** Frequency histograms of the forward predicted reward for the four reagent injection options in the cALD chemistry exploration campaigns. The red arrow indicates the path taken when exploiting the agent. Upstream injections assume prior injections followed the exploited path. The learning agent was trained on the full data set of the cALD chemistry exploration campaign.

The sequence selection campaign was conducted for 140 microdroplets/experiments, with a maximum of 20 injections per microdroplet. First, every combination of three sequential injections were conducted to provide an initial data set for the RL agent. From there, RL algorithms were used with UCB1 as the decision policy to build a rapid and accurate understanding of the reactions and possible optimal routes. As shown in Fig. 3a, the agent quickly identified unviable early injection sequences and directed exploratory experiments toward more favorable paths. After 920 total injections, the RL agent was exploited for one microdroplet/experiment, without a limit on the number of injections. In the exploitation experiment, the RL agent selected six repeating cycles of the sequence: OAm-Na_2_S-FAm-Cd(Ac)_2_-OAm, which bears similarities to the conventional cALD consecutive half-cycle method (OAm-Na_2_S-FAm-FAm-Cd(Ac)_2_-FAm-FAm), illustrated in Fig. 3e. The RL-selected sequence mimics the half cycle structure posed in literature^53,54^, where an initial sulfide layer is added and removed, then a cadmium layer is added. Additionally, the first three injection steps of the RL-selected sequence by AlphaFlow are identical to the conventional sequence, which, given the systematic exploration of these injections applied in the pretraining data set, validates aspects of literature methods. However, the AlphaFlow*-*discovered cALD sequence also features several notable differences from the conventional cALD chemistry.

First, one of the FAm washing steps that occur after the first half cycle of reagent additions (OAm-Na_2_S) of the conventional cALD chemistry is removed. The one experiment where two wash steps were performed after the first half cycle (OAm-Na_2_S) reached a terminal condition after the addition of OAm. It is possible that the exploration of this sequence was terminated by the RL agent. Regardless, the development of the single wash step of colloidal QDs enabled continued cALD cycling with the retention of optical features.

Second, AlphaFlow discovered that the two FAm wash steps after the addition of CdAc_2_ could be replaced by a single OAm injection. In the conventional cALD chemistry, OAm is added to form ionic complexes with chalcogenide ions (S^2−^) and cadmium reagents (Cd(Ac)_2_), which enables the phase transfer and subsequent reaction of ions at the nanoparticle surface. OAm also serves to stabilize charged nanoparticle surfaces in the nonpolar phase. It is proposed that excess stabilizing ligands (OAm) in solution, without sufficient washing of the nanoparticle phase, can cause the retention of ionic complexes of each half-cycle reagent in the nonpolar phase, leading to unwanted secondary nucleation and formation of CdS nanoparticles, as well as poorly controlled shell growth^53^. Thus, in conventional cALD chemistry, only enough OAm is added to enable surface reactions and retain the colloidal stability of nanoparticles in the nonpolar phase. However, the synthetic route chosen by AlphaFlow here indicates a broader function of stabilizing ligands during the multi-step cALD chemistry. That is, replacing a wash step with an additional OAm injection enables continued cALD cycling, with an improved first absorbance peak-to-valley ratio (i.e., better nanoparticle size uniformity), despite one OAm injection being sufficient to achieve surface reactions in the first cALD cycle and retain colloidal stability. Finally, the AlphaFlow-discovered cALD chemistry is two full injections shorter than the conventional cALD chemistry, which translates into a lower total experimental cost over multiple cycles.

Due to the size of the parameter space in this system (Fig. 1), which exceeds 10^12^ possible sequences, identifying and proving a global optimum is infeasible. However, compared to a conventional cALD sequence, AlphaFlow achieved considerable improvements without prior literature knowledge. Shown in Fig. 3b, a conventional cALD sequence optimized based on literature protocols was compared to the AlphaFlow discovered route for six consecutive cycles. For the starting CdSe QDs used in this study, the first absorption peak shift plateaued after three cycles (i.e., halted shell growth) in the conventional cycling, while the RL-selected cALD sequence continued the CdS shelling for all six cycles. This continued growth enabled a first absorption peak wavelength shift that is 26 nm higher and a photoluminescence intensity that is 450% higher than the conventional cALD sequence. Furthermore, the AlphaFlow-selected cALD chemistry resulted in an on average 9 nm larger peak shift (i.e., thicker shell growth) per cycle in the cycles preceding the conventional sequence plateau, despite implementing a shorter reagent injection and wash sequence—Fig. 3c, d. Detailed comparisons are shown in Supplementary Fig. 13.

For these studies, oleic acid-capped CdSe QDs were used in a diluted 0.007 mM solution over the course of weeks^55^. However, when the CdSe solution was freshly diluted (<2 days old), neither sequence’s λ_AP_ plateaued within six cycles, although the RL sequence still had improved *I*_PL_ and was less affected by Na_2_S*9H_2_O/FAm solution aging under N_2_ compared to the conventional cALD sequence (Supplementary Fig. 14). Combined with the differences between the RL-selected sequence and the conventional cALD cycling, these results lead us to consider the starting surface chemistry and sulfide solution byproducts as important considerations to cALD sequence optimization and the basis for improvements in the RL-discovered sequence route. Specifically, we hypothesize that the role of decreased washing and increased OAm, chosen by AlphaFlow and not in literature methods, is multifaceted. No-wash steps and increased OAm after Cd(Ac)_2_ could serve to form and retain oleate ions in solution, which passivate surface defects introduced by S^2−^ byproducts through Cd-oleate and primary amines. A wash step is still necessary after the sulfide addition to avoid increased chalcogenide reactant availability, which can lead to a transition to kinetic growth^56^. However, interestingly, even without a wash step after the Cd(Ac)_2_ addition, the eV change per cycle of the RL sequence closely resembles successive ionic layer adsorption and reaction (SILAR) protocols^57^ optimized to avoid homonuclei formation at elevated temperatures and correlates to sub-monolayer growth (Supplementary Fig. 15). These results suggest that OAm plays a key role in preventing the formation of homonuclei in solution, which could be formed from the reaction of metal reagents with the hydrogen sulfide in the aged sodium sulfide solution. OAm-sulfide complexes alone may preferentially react at the CdSe surface than form homonuclei at room temperature, as well as make reactive sulfide sources available in the aged solution by preventing polysulfide formation^58,59^. In this way, the cALD limiting half cycle is the sulfide-reagent half cycle, while excess Cd^2+^ and OAm in solution aid surface mobility of reactants and passivation to improve crystalline monolayer growth. Additional studies using AlphaFlow with different chalcogenide sources, stabilizing ligands, and reaction temperatures will likely provide more fundamental insight into complex cALD and SILAR-based reactions.

The sequence selection behaviors of AlphaFlow can be better understood by evaluating the forward reward prediction at each step in the optimized cALD sequence. The algorithm simulates a collection of action sequences four steps into the future and selects the next injection that produces the greatest predicted future reward, shown in Fig. 3f. This approach quickly filters out conditions with known detrimental effects, such as the injection of Na_2_S without OAm (which causes the phase transfer of QDs) and directs selection towards more consistent reward increases. The RL algorithm also distinguishes between neutral investment conditions, such as the injection of FAm at the first step, and conditions that provide improvements further in the future, such as the injection of OAm. Such delayed benefits do not appear when the predicted reward is mapped out fewer steps into the future, see Supplementary Fig. 16, demonstrating the need for predicting rewards multiple steps ahead.

### SDL case study 2: autonomous multi-step synthesis-property mapping

Following the autonomous discovery of the cALD injection sequence, an RL-guided reagent volume and reaction time optimization campaign was performed by AlphaFlow to further improve the spectral properties of hetero-nanostructures achieved in the synthetic route discovery campaign by tuning the reaction conditions at each cALD cycle. These closed-loop experimental campaigns used the RL-identified cALD chemistry with three different starting CdSe QD sizes. Experimentally accessible volume and time ranges of 1 to 10 µL and 40 to 400 s (corresponding to 1 to 10 microdroplet oscillations in the synthesis fluidic micro-processor) were used, respectively. An experimental budget for RL-guided exploration of ~700 injection steps was given for each of the three different starting CdSe QD sizes. The non-invasive, in situ spectral characterization, enabled access to the nanoparticle properties at each droplet oscillation, transforming 700 injection steps to over 9000 total experimental conditions to be used in ML model training. Like the cALD sequence exploration campaigns, after running exploratory policies, the models were exploited to identify optimal conditions. Within these campaigns, the STM was cycle number and injection number dependent so that each step of the cALD sequences were individually optimized. This high-dimensional approach proved to be necessary as each injection step had an optimal volume and reaction time, which was different depending on the cycle number and hetero-nanostructure core size. Full exploited conditions sets are shown in Supplementary Table 2.

**Fig. 4:**
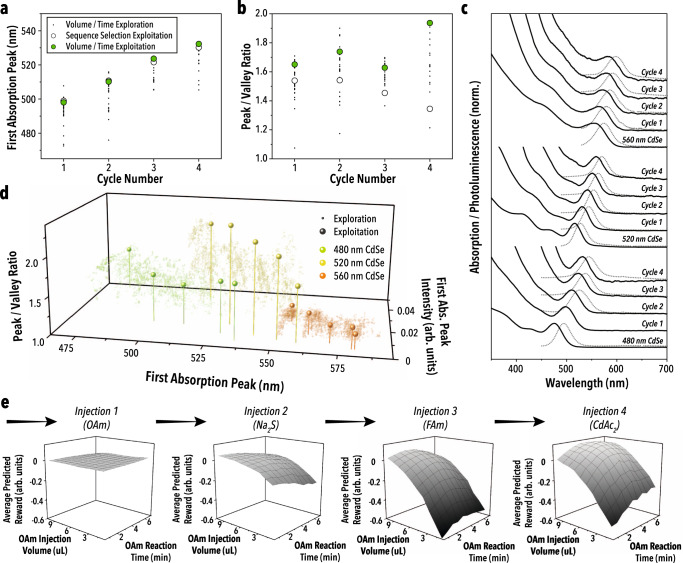
Volume and time optimization campaign results of AlphaFlow. **a** First absorption peak wavelength and peak-to-valley ratio (**b**) as a function of the full cALD cycle number for the volume and time exploration and exploitation and the cALD chemistry exploitation. **c** Absorption and photoluminescence spectra for the complete cALD cycles of the volume and time exploitation runs on each of the three starting CdSe nanoparticle sizes studied. **d** Output parameter space for the exploration and exploitation of the three CdSe nanoparticle sizes. **e** Average predicted reward for a single step in the volume and time optimization campaign as a function of the injection volume and reaction time of the first injection (OAm). Note that surface plot colors are a topographic guide to the eye. Injecting OAm has little immediate influence on the measured reward, but forward predicting ahead shows that the decision significantly affects downstream reward. The RL agent was trained on the full data set for the 480 nm CdSe nanoparticle volume and time optimization campaign.

For the first tested CdSe QD size with a starting λ_AP_ of 480 nm, the exploitation experiment resulted in a λ_AP_ shift equivalent to the cALD sequence selection exploitation results, while simultaneously producing a 40% higher *R*_PV_ by the fourth cALD cycle, shown in Fig. 4a, b. Furthermore, the exploitation experiment produced nanoparticles with spectral features in the upper regions of all conducted measurements, suggesting a successful exploitation of the cALD parameter space by AlphaFlow. Similar results were found for the two other CdSe QD samples tested—shown in Supplementary Fig. 17 and Fig. 4c, suggesting that the methods employed by AlphaFlow are directly transferable to other starting nanoparticles with spectroscopic metrics.

In some injection steps, AlphaFlow-selected conditions that would temporarily lower *R*_PV_ so that a higher *R*_PV_ could be achieved in later cALD steps. This result demonstrates the RL agent’s ability to select steps that are not immediately favorable but enable higher rewards downstream. Like the sequence selection study, forward prediction in the RL agent plays a critical role in achieving high rewards. As shown in Fig. 4e, short-term reward prediction insufficiently details the impact of a chosen set of injection conditions. For the first injection in the sequence, there is no discernable difference in the predicted reward for different OAm injection volumes and reaction times. However, those volumes and reaction times determine if a step is terminal two or three injections ahead. Moreover, the optimal conditions are not simply an amount of reagent above a threshold which is sufficient to complete and promote surface reactions. For example, Fig. 4e shows that there is an optimal volume and reaction time for OAm that, if above or below, results in worse hetero-nanostructure properties, which are not apparent until further into the cALD cycling. Although some of these delayed negative effects are understood based on literature, such as the influence of too few ligands on colloidal stability, the algorithmic determination of these optimal conditions through prior methods poses a considerable challenge because of non-transferrable conditions and hidden states (such as reagent age and nanoparticle quality), as well as inconsistent protocol reporting. Therefore, in addition to being able to navigate the immense parameter space of multi-step reactions, AlphaFlow is also successful in its optimizations because of reproducible, in-house generated data. Furthermore, the optimized conditions presented could be closely replicated in a conventional batch reaction system (Supplementary Fig. 18), indicating that the synthesis routes identified by AlphaFlow are transferable to larger scales. Compared to the presented work, optimized conditions for other syntheses, including mass-transfer limited reaction, are likely not directly transferable to batch techniques. However, reaction pathways can still be explored and discovered. In addition, these systems would benefit from scaled-out techniques to make optimized conditions transferable^60,61^.

The performance of the RL algorithm relative to manual model-driven studies and closed-loop Bayesian optimization (BO) methods was tested using a digital representation of the experimental system. Using the data generated from the 480 nm QD volume and time optimization campaign, a digital twin model was trained to provide a prediction of the three output parameter values and the viability for each injection,, similar to the strategies employed in prior studies^52^. AlphaFlow’s digital twin was used as a stand-in for the real-world experimentation system in a digital environment. This technique allows for the performance of different optimization algorithms to be compared without requiring an excessive number of real-world experiments. Within this context, function evaluations are limited, prohibiting the effective use of methods such as genetic algorithms. In addition, there are few guarantees about the function’s properties to be optimized, such as convexity. Within this evaluation-limited, high-dimensional experimental context, BO and local searches, such as basin-hopping, are considered state-of-the-art methods for autonomously optimizing physical experiments. Therefore, it is critical to investigate and demonstrate the shortcomings of such methods in high-dimensional experimental spaces.

The first algorithm used on this system was an ENN-based BO method. Using the UCB policy and a model structure similar to the one used in the RL agent, an ENN model was trained on the local reward after all 20 reagent injections, which is equivalent to a 40-dimensional input space. Predictably, the high-dimensional parameter space proved to be too large a challenge for standard BO methods. After 100 experiments (equivalent to 2000 total injections) the BO algorithm failed to identify a set of 20 consecutive viable injection conditions, resulting in no measured reward—see Supplementary Fig. 19. The AlphaFlow algorithm, however, achieved a viable set of 20 reagent injection conditions after only four experiments, with a local reward that is 87% of the known optimum, and continued to improve the material quality throughout 100 total experiments to a final reward that is 94% of the known optimum. In total, the RL algorithm identified 12 viable condition sets out of the 100 attempted experiments. Although BO is likely to perform better in optimizing only over a six-injection cycle, equivalent to one full cALD cycle, this strategy would not be reflective of optimizing through a 20-injection budget. That is, optimizing within one full cALD cycle using BO does not account for the long-term effects of actions taken in each cALD cycle. The RL campaigns with 20-injection sequences include a moving window of STM and forward predictions that can map the long-term effects of prior cycle injections to predicted rewards. It is likely that if the RL agent only had a six-injection budget, i.e., without the moving STM mapping, the optimized six-injection sequence would be different but worse when extrapolated through multiple cycles. It should also be noted that strategies like BO may perform better if given the opportunity to sample from the environment, as the surrogate was built from RL-based experimentation data.

Next, the importance of RL-based dimensionality reduction as well as real-time decision-making, were demonstrated by exploiting the digital twin for an optimized set of reaction conditions. The digital twin optimum was calculated through 20 replicates of a basin hopping function maximization using the Limited-memory Broyden–Fletcher–Goldfarb–Shanno algorithm (L-BFGS)^62^. Similar to the BO algorithm, the basin hopping function attempted to maximize the local reward after 20 consecutive reagent injection conditions. It should be noted that this global optimum method could only be applied in a digital environment as over 50,000 simulated experiments (equivalent to 1,000,000 reagents and solvent injections) were required to reach the final optimum. Comparatively, since the RL-digital twin campaigns required only 100 total experiments to reach 94% of the optimum, the opportunity cost of using RL-based algorithms in a closed-loop environment is greater than global optimization strategies. It is likely that by allowing for more experimental campaigns with the digital twin, the AlphaFlow algorithm would reach a reward closer to the optimum.

In a manual model-driven study, an experimentalist will compile all available data and train a model to illustrate strong predictability. Then the model will be exploited to generate a full set of test conditions to be used in real experiments. To evaluate this strategy with cALD, the basin hopping optimum conditions were tested in AlphaFlow’s platform. As shown in Supplementary Fig. 20, the parameters predicted by the digital twin showed strong agreement with the experimental values for *R*_PV_ and λ_PI_, with the 20 reagent injection predictions falling within 0.04 and 0.008 of the measured values, respectively. However, an overly optimistic prediction was made for λ_AP_, and the 20-injection measurement deviated from the prediction by 15 nm. Due to the inaccurately high λ_AP_ prediction, the local reward from the basin hopping optimum prediction was 10% higher than the same conditions performed in AlphaFlow’s real-world closed-loop platform. Additionally, both the predicted and real-world basin hopping optimum local rewards were lower than that of the real-world AlphaFlow conditions. This result could be attributed to the real-time adaptation of AlphaFlow experiments to updates in the material states, illustrating the importance of closed-loop experimentation strategies. Extensive exploitation of a high-dimensional model does not account for deviations that are likely to occur during experimentation, especially in complex and sensitive multi-step nanomaterial syntheses, which tend to have nondeterministic actions compared to organic syntheses and other RL-based applications such as multi-step strategy games. Updating the belief models during RL-guided experiments by AlphaFlow provides the necessary adjustments for these deviations and allows for more precise tuning of reagent injection conditions. In addition, accounting for real-world deviations in complex parameter space can aid in building foundational knowledge about the nature of complex hidden states.

## Discussion

The intelligent robotic research assistant presented in this work helps to resolve a dimensionality barrier in algorithm-guided multi-step chemistry and enables SDLs to optimize and discover synthesis routes in more complex, sparsely populated problem spaces. AlphaFlow effectively explored and optimized a 40-dimensional parameter space with a chemical consumption of less than one five-hundredth of what is needed through manual methods and at a data generation rate equivalent to the throughput of more than 100 researchers working simultaneously. Coupling this experimental capability with real-time, intelligent decision-making enabled control over a reaction space with complexity well beyond all prior SDL studies. Further implementation of the SDL presented in this work will enhance the efficiency with which high-dimensionality, dynamic, multi-step reactions may be studied, thereby extending the intellectual reach and innovative capabilities of researchers and leading to novel insights into multi-step processes and advanced functional materials. As an example, AlphaFlow enables facile future exploration and exploitation studies of cALD-based chemistries, previously limited by large parameter spaces, dynamic reactions, and arduous experimentation to create next-generation high-performance semiconductor nano-heterostructures for applications in energy and chemical technologies. Beyond intelligent experimentation, the high-throughput data generation capabilities of AlphaFlow alone may be useful for other algorithm-guided studies and fundamental insights into multi-stage chemistries. To perform such data mining of SDL-generated information in an efficient and reproducible way, the eventual creation of standardizations in (meta)data reporting and experimental setups would be beneficial.

In addition, this work demonstrates the potential of RL in solving complex, multi-step reactions, enabling algorithm-guided exploration of syntheses that may be influenced by dynamic time scales, hidden states, synthesis step order, or even unstable intermediates and reagents. For example, outside of colloidal nanoscience, the presented RL approaches may be of value to conventional atomic layer deposition and molecular layer deposition processes. Although these processes are usually carried out in self-limiting growth regimes, it is possible that novel insights about film composition and performance could be found by using RL algorithm-based exploration of reactant sequence, partial pressure, temperature, purging, and exposure times. Furthermore, AlphaFlow has applications in telescoped reactions, where unstable intermediates form time-sensitive, hidden states that are critical components of the reaction system.

## Methods

### Chemical preparation

The full chemical inventory is included in Supplementary Note 2.

#### Formamide degassing

All formamide used in reagent preparation and washing in the reaction system was first degassed and flushed with nitrogen. This process was carried out by first degassing 80 mL of formamide under a vacuum for 18 h with vigorous stirring. After degassing, the vial was repressurized with nitrogen and then held under a vacuum for 30 s. This cycle was repeated three times before the final pressurization.

#### Toluene drying

All toluene used for reference collection, CdSe dilution, and the OAm mixture was dried over molecular sieves for at least 48 h before use.

#### Sulfide reagent

The sulfide reagent was prepared by adding a stir bar and 200 mg of sodium sulfide nonahydrate into a 25 mL round bottom flask with a septum. While under vacuum, 20 mL of degassed formamide was injected into the sodium sulfide flask. The flask was then left under vacuum and vigorous stirring for 2 h, followed by three cycles of nitrogen pressurization and flushing. After final nitrogen pressurization, the reagent was wrapped in parafilm and loaded into the reactor system, and left to sit for an additional 5 h before use.

Note that the sulfide reagent is sensitive to environmental exposure and aging time. After the stirring step, the solids should be completely dissolved, and the solution should be transparent. If the solution has any solids remaining or a slight yellow tint, the reagent will not behave as reported. Additionally, the vial cannot be agitated or moved after loading into the reactor.

#### Cadmium reagent

The cadmium reagent was prepared by adding 30 mg of cadmium acetate to a 15 mL glass vial with a septum. While under vacuum, 10 mL of degassed formamide was added to the vial. The vial was held under a vacuum and swirled occasionally until all solids were fully dissolved—approximately 3 min. The vial was then pressurized with nitrogen and wrapped in parafilm before use.

#### Oleylamine-toluene mixture

The OAm solution was prepared under ambient conditions by adding 9.25 mL of toluene to 750 µL of OAm and shaking until combined.

#### CdSe quantum dot synthesis

The synthesis procedure was adopted from the previous methods^63^. A Cd precursor was prepared by dissolving 0.240 g CdO (1.87 mmol), 2 mL OA (6.30 mmol), and 10 mL ODE (31.25 mmol) in a 250 mL three-necked round bottom flask and heated to 100 °C under vacuum for 30 min while stirring. Simultaneously, a Se precursor was prepared by dissolving 0.100 g Se (1.27 mmol) in 10 mL ODE in a 250 mL three-necked round bottom flask and heated to 100 °C under vacuum for 30 min while stirring. After the elapsed 30 min, the Cd precursor was exposed to nitrogen and heated until the solution turned colorless (~200 °C) and then decreased to 100 °C. Simultaneously, the Se solution was exposed to nitrogen and heated slowly to 300 °C and maintained at that temperature until all the black Se powder was dissolved, and the solution turned a yellow color. Once the Se is dissolved, the Se precursor was reduced to 240 °C prior to injection, and a degassed syringe was used to inject 12 mL of the Cd precursor. The reaction mixture times were then monitored and altered to yield the desired wavelength. Once the necessary reaction time expired, the heating mantel was removed, and 40 mL of ice-cold toluene was injected to quench the reaction. The CdSe quantum dots (QDs) were allowed to cool to room temperature and washed. After the solution was cooled, the QDs were precipitated first by the addition of acetone (1 mL acetone:1.5 mL QDs) and centrifugation. Small pellets then precipitated out to the bottom, which was the excess OA, and the red solution was kept for further washing. To further remove excess OA, the QD solution was centrifuged again with no acetone, and the liquid was further separated from the OA. Then, acetone was added in a ratio of ~6 mL acetone:1 mL QD solution to further precipitate the QDs, and subsequent centrifugation was repeated three times. Ethanol was then added in the same proportions, and this process was repeated 3 times. After the final precipitation, the QDs were redissolved in ~5 mL toluene.

### Reactor operation protocols

The full equipment inventory used to build the system is included in Supplementary Note 3. A complete process flow diagram of the system configuration used in this study is shown in Supplementary Fig. 21.

Automated experiment conduction in the single microdroplet reactor is divided into distinct action modules that may be called in the desired order. These modules are initial nanoparticle injection (Supplementary Figs. 3A–4C), additional reagent injection (Supplementary Fig. 5A–D), optical spectra collection & oscillation (Supplementary Fig. 6A–D), phase separation (Supplementary Fig. 7A–D), droplet waste and reactor cleaning (Supplementary Fig. 8A–E), and syringe refilling (Supplementary Fig. 9A–C). An alternative protocol enables droplet oscillations without sampling, which allows for longer reaction time studies, but for the purpose of generating data in this study, sampling was conducted with each oscillation. Additionally, a sub-protocol is called whenever the position of the primary selector valves needs to change.

Changing the positions of the primary selector valves requires a specific sequence to avoid droplet breakup in the downstream channel (Supplementary Figs. 3A–9C). During regular reactor operation, pressure variations can develop among the isolated injection channels. For example, if a small pressure decrease occurs in an injection channel due to regular leakage, then the downstream selector valve is switched to the low-pressure channel, and the droplet will rapidly move upstream during pressure equilibration. This rapid movement often causes the droplet to separate into several smaller droplets, thereby terminating the experiment prematurely. To account for this challenge, an upstream pressurization valve was added before the upstream primary selector.

Additionally, throughout each experiment, the system must identify where the droplet is positioned in the reactor with a high degree of precision (mm and ms scale position and timing). This is done by reading the voltage output through a collection of low-cost, infrared phase sensors positioned throughout the system. The phase sensors operate by waiting until the voltage reading increases past a specified threshold, indicating that the droplet is at the position of the phase sensor, and remains past that threshold for a set duration—approximately 200 ms. The threshold is set every time the phase sensor is called by taking the current reading, which presumably is of an empty tube, then adding 0.4 V. This method proved to be robust for continuous reactor operation, but further operational consistency was achieved by timing the individual steps in each of the protocols and only calling the phase sensors within a time window where the droplet is expected it appears.

### Reference collection, reactive phase isolation, and feature isolation

The formamide and toluene absorption references are collected by injecting a 10 µL droplet of formamide into the reactor, collecting five replicates of absorption data, injecting a 10 µL droplet of toluene, then collecting another five replicates of the absorption data. During the sampling process, spectra are continuously collected over approximately 4 s, so many saved spectra are taken off the carrier gas. To isolate the droplet, all spectra with light source signal intensities at 770 nm above 36,000 counts are saved—see Supplementary Fig. 22A. The final spectra for each sample collection are calculated by removing the highest and lowest 90% of counts for all measured wavelengths. Then, the final reference spectra are calculated by averaging the spectra from all five replicates—shown in Supplementary Fig. 22B, C. During the operation of the experimental system, new reference spectra would be collected every time the user came in physical contact with any part of the system and at least once every 24 h.

In addition to calculating the Beer–Lambert absorption spectra, these references are used for phase isolation on reactive droplets during regular system operation. Like the reference phase extraction method, the signal intensity of the biphasic droplet at 770 nm is used to identify the reactive phase. All spectra collected with absorption counts within ±2000 of either reference at 770 nm is grouped with the corresponding reference phase. The same trimmed mean procedure is used to isolate the relevant spectra from the phase subgroups. The final reported spectra are calculated using Beer–Lambert absorption (*A*):

1 $$A=-{{{{{{\rm{log }}}}}}}_{10}\left(\frac{{I}_{{{{{{{\mathrm{Sample}}}}}}}}-{I}_{{{{{{{\mathrm{DR}}}}}}}}}{{I}_{{{{{{{\mathrm{LR}}}}}}}}-{I}_{{{{{{{\mathrm{DR}}}}}}}}}\right)$$

Where *I*_Sample_ is the isolated reactive phase spectra, *I*_DR_ is the absorption dark reference, and *I*_LR_ is the absorption light reference, for formamide or toluene.

Photoluminescence spectra are extracted similarly, except there is not a clear feature in the raw spectral data at any wavelength that can be consistently used to identify the reactive droplet phases. Instead, the spectra are sorted by photoluminescence intensity, and five samples with the highest peaks in the expected photoluminescence range (480 to 680 nm) are averaged with a 50% trimmed mean.

Due to the size of the data sets and the autonomous approach used in this work, robust methods for automating the extraction of spectral features are critical. The following methods were optimized to produce consistent identification of features across a diverse set of spectra. First, a third-order polynomial Savitzky-Golay filter with a 21-frame window was used to smooth the absorption spectra—shown in Supplementary Fig. 23. Note that all reported spectra in this manuscript have not been smoothed, but smoothing was applied for feature extraction.

The smoothed spectra were then resampled using an antialiasing lowpass filter through the Matlab (Version 2021b) function *resample* to interpolate between spectra measurements. Next, the first absorption peak position was detected using the Matlab function *findpeaks* with a minimum peak prominence of 0.002. The prominence filter was applied to ensure that local maxima due to noise were not included in the set of potential peaks. Finally, the first absorption peak position is assigned to the highest wavelength detected peak within the range of 350 to 750 nm. This position is also used for the absorption peak height. For the peak-to-valley ratio, the valley height is calculated by measuring the lowest absorption value in the range of 100 nm before the first absorption peak wavelength.

### Terminal condition metrics

For training the classifier model and setting penalties in the regressor, unviable reaction conditions, also referred to as terminal conditions, need to be distinguished from viable conditions. An experiment is labeled terminal if any of the following are true:

1. Less than 75% of the total droplet is assigned to either the toluene or formamide phase. The total length of the droplet is measured through the light source signal at 770 nm, where all spectra with values above 30,000 counts are considered part of the droplet. If 75% of the spectra do not fall within the formamide and toluene ranges specified in the section above, then there is a high probability that the solution has become colloidally unstable.
2. Less than 25% of the total droplet is assigned to the toluene phase. This condition can occur when there is colloidal instability in the toluene phase, or there is not enough toluene to consistently continue the experiment. Toluene can be lost throughout experiments due to absorption into the formamide phase or imperfect phase separation steps.
3. There are no detectable first absorption peaks, using the minimum peak prominence filter. Peaks at the boundary of the 350 to 750 nm range are not included.
4. The absorption signal at 350 nm is below 0.03. If the concentration of the quantum dot is low enough, signal to noise ratio can become too low to effectively continue measurements. The concentration of quantum dots can decrease if there is an excessive dilution of the toluene phase or, more commonly, there is a dropout into the formamide phase.

### Droplet length measurement

Droplet phase lengths (*L*_Tol_ and *L*_FAm_) are calculated using the droplet velocity from the phase sensors (*u*_Droplet_) and the phase passing time (*t*_(Pass,Tol)_ and *t*_(Pass,FAm)_) from the absorption spectra. Velocities are calculated by measuring the time to pass from the phase sensor at the beginning of the reactor spiral to the phase sensor before the flow cell (*t*_(ReactorTransit)_), which has a fixed tubing length of 55 cm. This velocity is measured with every optical sampling cycle. The phase passing times are calculated by measuring the integral of the sampling time and a binary array corresponding to positively identified phases in the time-resolved absorption spectra. The droplet length is simply calculated with:

2 $${u}_{{{{{{{\mathrm{Droplet}}}}}}}}={L}_{{{{{{{\mathrm{Reactor}}}}}}}}/{t}_{{{{{{\mathrm{Reactor}}}}}}\;{{{{{\mathrm{Transit}}}}}}}$$

3 $${L}_{{{{{{{\mathrm{Tol}}}}}}}}={{u}_{{{{{{{\mathrm{Droplet}}}}}}}}t}_{{{{{{{\mathrm{Pass}}}}}}},{{{{{{\mathrm{Tol}}}}}}}}$$

4 $${L}_{{{{{{{\mathrm{FAm}}}}}}}}={{u}_{{{{{{{\mathrm{Droplet}}}}}}}}t}_{{{{{{{\mathrm{Pass}}}}}}},{{{{{{\mathrm{FAm}}}}}}}}$$

Using this method, 29 randomly selected formamide and toluene injection volume combinations, each ranging from 3 to 10 µL, were briefly oscillated at 800 µL/min and measured for their phase length. As shown in Supplementary Fig. 24A, B, the measurement technique shows a strong linear relationship between the injection volume and the measured phase length. The toluene, which for this study used a timed injection from a continuous flow carrier pump, showed a slightly higher variance than the formamide injection, which used a high-end syringe pump with a 500 uL glass syringe. However, this discrepancy is likely due to the injection precisions of the two methods and not a factor of the technique itself. A second test was run using a single 3 µL toluene droplet—injected with a syringe pump—with repeated droplet length measurements at randomly selected volumetric flow rates (Supplementary Fig. 24C). The mean length prediction does not scale with the droplet velocity—a linear fit of the data set results in a slope of 1.9 × 10^−7^ cm/[uL/min]—but the measurement variance increases with higher flow rates. This change is likely associated with the sampling step time resolution.

To ensure that solvent loss does not occur during regular reactor operation, droplet length measurements were taken over 50 to 100 oscillations through various sections of the reactor. Full details are shown in Supplementary Note 4 and Fig. 25.

### Phase separator

Phase separator operation relies on a timed reversal of the primary carrier flow pump while the separator pump continues to flow forward. The timing of this reversal is based on the measured formamide phase length, as determined by the most recent optical sampling protocol, and the separator delay calibration curve. This curve was calculated by testing 16 biphasic droplets composed of 6 µL of toluene and 6 µL of formamide. For each of the droplets, a random flow reversal delay time (*t*_Delay_) from 1600 to 2600 ms was applied in the separation protocol. By measuring the total droplet length before and after separation for each of the delay times, a specific change in droplet length (∆*L*_Droplet_) was associated with a specific delay. As shown in Supplementary Fig. 11A, this relationship was fitted to produce the equation:

5 $${t}_{{{{{{{\mathrm{Delay}}}}}}}}=\frac{\triangle {L}_{{{{{{{\mathrm{Droplet}}}}}}}}+1.23\;{{{{{{\mathrm{cm}}}}}}}}{0.00105\frac{{{{{{{\mathrm{cm}}}}}}}}{{{{{{{\mathrm{ms}}}}}}}}}$$

Operation of the adaptive phase separation system applied a modified version of this calibration curve, which used the measured formamide phase length and a 0.1 cm removal buffer:

6 $${t}_{{{{{{{\mathrm{Delay}}}}}}}}=\frac{{L}_{{{{{{{\mathrm{FAm}}}}}}}}+1.13\;{{{{{{\mathrm{cm}}}}}}}}{0.00105\frac{{{{{{{\mathrm{cm}}}}}}}}{{{{{{{\mathrm{ms}}}}}}}}}$$

The adaptive separation system was tested by conducting separations on biphasic droplets of random toluene and formamide volume combinations—the same droplets that were used to generate Supplementary Fig. 22A, B. Phase lengths were measured before and after the separation protocol for each. As shown in Supplementary Fig. 11B–E, this method produced consistent retention of the toluene phase and near-complete removal of the formamide phase.

Note that with minor modifications to the flow reversal timing and flow balancing arrangement, this method is applicable to the removal of the alternate phase, i.e., the encapsulating phase.

### Reaction conduction precision

Successful navigation of a large parameter space requires a high degree of precision in experiment conduction^44^. Because the case study system relies on specifying a sequence of reagent injections, it is important to verify that a given sequence will reproducibly result in a specific set of optical features. As shown in Supplementary Fig. 26, five replicates of conventional full cycles were conducted on the reactor. Throughout the entire cycle, all five replicates produced optical features within proximity to each other. The final spectra of the cycles, after seven sequential injections, had standard deviations of 0.004 for the absorption intensity at 350 nm, 0.01 for the peak-to-valley ratio, 0.4 nm for the first absorption peak position, 0.002 nm for the absorption half-width at half-maximum, and 2.5 s for the experiment conduction time (after 57 min of continuous operation each cycle).

Similarly, it is also important to verify that experiments may be conducted independently of each other. If, for example, a specific set of reaction steps caused fouling of the reactor channel that could not be sufficiently removed, then the next experiment would not behave as expected. To verify the efficacy of the washing protocol and the independence of each new experiment, four random injections were added to a new droplet, then the washing/waste protocol was applied, and a full cycle was conducted on a new droplet. This sequence was repeated five times (Supplementary Fig. 27). Despite using a different injection sequence in between each full cycle experiment, the full cycles showed a reproducibility similar to the consecutive full cycles across all optical features. While there was no visible aggregation on the tubing wall at any point during experiment conduction, the entire reactor tubing was replaced once every 1000 experiments. Additionally, consistency of the automatic refilling procedure of the precursors is critical to ensure continuous experimentation over an extended period by AlphaFlow. Supplementary Fig. 31 shows the reliability of the automated precursor refilling module of AlphaFlow over 14 refilling cycles.

### Sodium sulfide age consideration

All reagents and nanoparticles in this study had high stability within the timespans they were used, except for the sodium sulfide solution. Sodium sulfide, in formamide in this case, forms a diverse composition of byproducts depending on the moisture content, oxygen content, available ligands, and aging time^49^. Over time, the same reagent can produce varied results for the same injection conditions. After preparing a new batch of sodium sulfide reagent, the first sequence of a conventional half cycle was repeated continuously (CdSe > OAm > Na2S > FAm > FAm) over 60 h. The final optical features after each half-cycle are shown in Supplementary Fig. 28A, B. Early half-cycles result in a lower first absorption peak and peak-to-valley ratio than those of later cycles. Furthermore, our prior work with Na_2_S reagents has indicated that variable results are expected beyond the 60 h maximum shown. However, if the data is isolated to the aging range of 4.5 to 60 h, the variance across all half-cycle end features is manageable—shown in Supplementary Table 3. For all experiments conducted in the reinforcement learning studies, sodium sulfide solutions prepared within 5 to 60 h were used.

### Reinforcement learning algorithm overview

A detailed description of the RL agent is provided in Supplementary Note 5, and all code used is available online (GitHub)^64–67^ at the address listed below. In summary, the algorithm operates by conducting three sequential steps: (1) formatting all new data sets for training, (2) building the belief model, and (3) executing the rollout policy.

The data formatting step generates a set of state-action pairs and the corresponding responses. The state is comprised of a machine-readable sequence of the three previous precursor injection conditions. In the sequence selection study, this short-term memory is formed by one-hot encoding for the four possible injections on each step, then the action, which represents the most recent injection, is also encoded, and added to the string. The final state-action pair used for model training is then a sixteen-member string of binary values. The volume and time optimization studies generated a similar state-action sequence, except the injection number and cycle number were tracked, each with an integer value. The short-term memory and action steps were generated by adding non-dimensional forms of the injection volumes and reaction times selected at each step. As a result, the state-action pair string for the volume and time optimizations comprised of two integer values, four continuous values for the injection volumes, and four discrete values for the reaction time (where each level is one full oscillation). Each of these strings were paired with a resulting response value, represented by either terminal classification, discussed previously, or the slope reward. The slope reward is calculated by conducting a linear fit on the local reward improvement (a weighted mean of the three target parameters, λ_AP_, *R*_PV_, and *I*_PL_, with only increasing values) for the eight previous measurements as a function of λ_AP_.

The belief model is built by training an ensemble neural network regressor, and gradient-boosted decision tree classifier on the fully formatted data set. Each member of the regressor ensemble is assigned a randomly selected architecture and a random training set comprised of 75% of the total available data set. The regressor is then trained to map state-action pairs to a resulting slope reward. The classifier is trained on the full data set and is set to map state-action pairs to a terminal or non-terminal condition.

The rollout policy evaluates the belief model predictions for future action sequences and returns a recommendation for the next action to take on the real system. Every possible set of action sequences for four actions into the future are evaluated by predicting the reward for each action in a branch. The performance of each action sequence branch is quantified by the highest achieved reward in the sequence. The action sequences are then grouped by their first action. A decision policy is then applied to the first action groups to determine which next action provides the most value. During reaction space exploration, an upper confidence bounds policy was used. This method seeks to maximize both the mean and the standard deviation of the predicted performance for each of the action groups, which is intended to direct experiments where there is both a high chance of achieving high-quality materials and a high chance of sampling in regions with greater model uncertainty. During exploitation experiments, which occurred after exploration, the decision policy sought only to maximize the mean predicted performance.

### Digital twin studies

#### Digital twin structure

The digital twin is composed of four models: the viability classifier, change in absorption peak wavelength regressor, absorption peak intensity regressor, and peak-to-valley ratio regressor—shown in Supplementary Fig. 30. The viability classifier uses the same structure used in the RL belief model. All three regressors use the same ensemble neural network structure as the RL belief model with the following modifications: The absorption peak wavelength, absorption peak intensity, and peak-to-valley regressors used a 10, 10, and 75% subsampling rate, respectively. All regressors had an ensemble size of 200, erroneous data not caught by the automated processing scripts was filtered out, and the ensemble mean prediction uses data trimming for all predictions outside one standard deviation from the median.

#### Bayesian optimization algorithm

The BO algorithm used in the digital twin study follows the same design implemented in prior work^11,52,68^. The belief model is a 20-member ensemble neural network with the same structure as that used in the RL belief model. The algorithm uses a UCB decision policy with the predicted value (*q*_UCB_) defined as:

7 $${q}_{{{{{{{\mathrm{UCB}}}}}}}}={\mu }_{{rL}}+\frac{1}{\sqrt{2}}{\sigma }_{{rL}}$$

Where *μ*_*rL*_ is the mean predicted reward for a set of input conditions and *σ*_*rL*_ is the standard deviation of the prediction. The belief model was trained on local reward after all 20-injection conditions are applied (i.e., 40 total input parameters).

### Reporting summary

Further information on research design is available in the Nature Portfolio Reporting Summary linked to this article.

## Supplementary information

Supplementary Information

Description of Additional Supplementary Files

Supplementary Movie 1

Reporting Summary

## Data Availability

The source data generated in this study have been deposited in the repository “AlphaFlow” (https://github.com/AbolhasaniLab).

## Code availability

The source code for the data formatting, reinforcement learning algorithms, and surrogate models have been deposited in the repository “AlphaFlow” (https://github.com/AbolhasaniLab). Within this repository, notebook demos of the sequence selection and volume and time optimization are also available.
