# Supplementary material for: AlphaFlow: autonomous discovery and optimization of multi-step chemistry using a self-driven fluidic lab guided by reinforcement learning
Source: Nat Commun. 2023 Mar 14;14:1403. doi: 10.1038/s41467-023-37139-y (PMC10015005; doi:10.1038/s41467-023-37139-y)
Supplement: Supplementary file 1 — Supplementary Information [file 41467_2023_37139_MOESM1_ESM.pdf]

Supporting Information

AlphaFlow: Autonomous Discovery and Optimization of Multi-Step Chemistry using a Self-Driven Fluidic Lab Guided by Reinforcement Learning

Amanda A. Volk [1], Robert W. Epps[1], Daniel T. Yonemoto[2], Benjamin S. Masters[2], Felix N. Castellano[2], Kristofer G. Reyes[3] & Milad Abolhasani [1*]

[1] Department of Chemical and Biomolecular Engineering, North Carolina State University, 911 Partners Way, Raleigh, NC 27695-7905, USA

[2] Department of Chemistry, North Carolina State University, Raleigh, NC 27695-8204, USA

[3]Department of Materials Design and Innovation, University at Buffalo, Buffalo, NY 14260, USA

**Corresponding author. Email: abolhasani@ncsu.edu*

Supplementary Notes

Supplementary Note 1 – Reactor Instruction Transcription 4

Supplementary Note 2 – Chemical Inventory 4

Supplementary Note 3 – Equipment Inventory and Operation 4

Supplementary Note 4 – Droplet Preservation 5

Supplementary Note 5 – Reinforcement Learning Algorithm 5

Supplementary Figures and Tables

Supplementary Table 1 – Function Instruction Format 12

Supplementary Table 2 – Optimized Volume-Time Conditions 13

Supplementary Table 3 – Precision of Half-Cycles in Operational Window 14

Supplementary Figure 1 – Material and Time Cost Summary 15

Supplementary Figure 2 – Microdroplet Reaction System 16

Supplementary Figure 3 – Selector Valve Position Change 17

Supplementary Figure 4 – Initial Droplet Injection 20

Supplementary Figure 5 – Precursor Injection 21

Supplementary Figure 6 – Optical Sampling 23

Supplementary Figure 7 – Phase Separation 25

Supplementary Figure 8 – Waste and Cleaning 28

Supplementary Figure 9 – Syringe Refill 30

Supplementary Figure 10 – Parallel vs. Serial Injection System 32

Supplementary Figure 11 – Phase Separator Validation 33

Supplementary Figure 12 – Illustration of reward function variants 34

Supplementary Figure 13 – Comparison of RL-selected and conventional cALD injection sequences 35

Supplementary Figure 14 – Cycling with Aged and Fresh CdSe 36

Supplementary Figure 15 – Optimized Results vs. SILAR 36

Supplementary Figure 16 – Sequence Selection Forward Mapping Levels 37

Supplementary Figure 17 – Complete Volume and Time Optimization Data 38

Supplementary Figure 18 – Batch Replication of Optimized Conditions 39

Supplementary Figure 19 – Bayesian Optimization vs. Reinforcement Learning with Digital Twin 40

Supplementary Figure 20 – Measured Values for Digital Twin Sequence 41

Supplementary Figure 21 – Full System Operation Flow Chart 42

Supplementary Figure 22 – Absorption Light Reference Collection 43

Supplementary Figure 23 – UV-Vis Absorption Spectra Pre-Processing 44

Supplementary Figure 24 – Droplet Phase Length Measurement Validation 45
Supplementary Figure 25 – Phase Retention in Mobile Droplets.. 46
Supplementary Figure 26 – Conventional Cycle Reproducibility 47
Supplementary Figure 27 – Reaction Conduction Independence 48
Supplementary Figure 28 – Sodium Sulfide Operational Window 49
Supplementary Figure 29 – General Structure of Reinforcement Learning Algorithm. 49
Supplementary Figure 30 – Digital Twin Training-Testing Regressions 50
Supplementary Figure 31 – CdSe absorption spectra after the syringe refill protocol 51
***Supplementary References*...51**

Supplementary Notes

Supplementary Note 1 – Reactor Instruction Transcription

The reactor control software is designed to perform any combination of functions with specified operation parameters. The instructions follow a sequenced recipe system that is conducive to further protocol additions, is flexible for troubleshooting, and is easy to adapt to other reactive systems. After setting the universal control parameters, such as file saving paths and equipment communication addresses, the reactor control software is placed in a standby mode where it waits for the function instruction sheet to be updated. The instructions are read and executed line by line, and upon completion of the current list of functions, the reactor returns to standby mode and waits for additional lines to be added. Shown in Supplementary Table 1, each function is identified by the first value in the row, then the remaining values represent function specific properties. For example, if the user wanted to clean the reactor, inject 6 μL of CdSe, collect spectra, inject 4 μL of Na_2S, oscillate, and collect spectra again, the instruction list would be:

4, 120, 1, 1
0, 0, 6, 100
3, 50, 800, 6, 20
1, 1, 4, 100, 2200
2, 5, 800
3, 50, 800, 6, 20

Supplementary Note 2 – Chemical Inventory

Toluene (*Fisher*, anhydrous 99.8%), formamide (*Fisher*, ACS 99.5%), sodium sulfide nonahydrate (*Sigma*, ≥99.99% trace metals basis), cadmium acetate (*Sigma*, anhydrous 99.995%), oleylamine (*Sigma*, >98% primary amine), cadmium oxide (*Sigma*, 99.5%), 1-octadecene (*Acros Organics*, 90% tech.), oleic acid (*Fisher)*, and selenium powder (*Sigma*, >99.5%) were used as received.

Supplementary Note 3 – Equipment Inventory and Operation

Syringe pumps (*Harvard*, PHD Ultra), continuous flow positive displacement pumps (*VICI*, CP2A-4141-F1HP), refill and pressurization valves (*Rheodyne*, MXT715-000 & MXT715-105; *Valco*, EUT-CSD4UW), primary channel selector valves (*Cheminert*, C25-3180EUHB), optical phase sensors (*Digikey*, OCB350L062Z), UV-Vis spectrometer (*Ocean Insight*, OCEAN-HDX-UV-VIS), absorption light source (*Ocean Insight*, DH-2000-BAL), photoluminescence excitation source (*Thorlabs*, M365LP1 & LEDD1B)

Custom-designed flow cell mount, reactor spiral, and tee junction holders were 3D printed in polylactide. Custom-designed flow cell module and pressure vessels were machined in aluminum. A fluorinated ethylene propylene (FEP) tubing (1/16” outer diameter, OD, × 0.030”

inner diameter, ID) was used to construct the flow reactor module of *AlphaFlow* with a length of 55 cm. The precursor formulation module used Teflon Tee junctions (IDEX natural PEEK Tee with a 0.040” bore diameter). For sampling and oscillations, a nitrogen carrier flow rate of 50 µL/min was used. Sample integration times for *in-situ* characterization were 800 ms for absorption and 48 ms for photoluminescence (PL) spectroscopy. For the injection of precursors/solvents in the formulation module, an injection flow rate of 100 µL/min was used. The pressure vessels and the flow reactor were pressurized by nitrogen (22 psig).

Supplementary Note 4 – Droplet Preservation

Single droplet experimental systems have a high propensity for losing solvent volume during regular reactor operation. In some cases, there can be a significant loss due to solvent evaporation at the gas-liquid interface, film formation on the trailing edge of the droplet, or droplet break-up within the channels. The prevalence of this issue in the current system was evaluated by oscillating a single droplet through the reactor and continuously monitoring the droplet phase length. Shown in Supplementary Figure 25A and 25B, both a single droplet and a biphasic toluene and formamide droplet could be oscillated through the reactor spiral continuously without appreciable volume loss for 1.6 h. Shown in Supplementary Figure 25C and 25D, the same droplets could be continuously passed through the selector valve without any notable loss of volume, indicating that breakup and evaporation play a negligible role in droplet loss. It should be noted that during early testing of the adaptive separation system, continuously adding and separating formamide from a single toluene droplet would result a toluene volume loss equal to approximately 2% - 4% of the injected formamide volume. This change was equivalent to the adsorption of toluene into formamide observed in batch. Additionally, the volume loss did not occur when the injecting formamide was saturated with toluene before use. Unsaturated formamide was used in the experimental system as the toluene-oleylamine precursor injection volumes sufficiently compensate for this adsorption.

Supplementary Note 5 – Reinforcement Learning Algorithm

Reward Function

The reward function used in the sequence selection algorithm is based on the slope of a local reward term as a function of the first absorption peak intensity. First the measured optical features first absorption peak (y_{AP}), first absorption peak intensity (y_{API}), and absorption peak to valley ratio (y_{PV}), are converted into non-dimensional and weighted terms (Y_x):

$$Y_{AP:i,j} = a_{AP}(y_{AP:0,j} + |y_{AP:i,j} - y_{AP:0,j}| - b_{1,AP})/(b_{2,AP} - b_{1,AP}) \quad (1)$$

$$Y_{PV} = a_{PV}y_{PV}/b_{PV} \quad (2)$$

$$Y_{API} = a_{API}y_{API}/b_{API} \quad (3)$$

Where i is the injection number for droplet j, and a and b are parameter weights and scaling factors respectively with constant values of:

$$b_{1,AP} = 470\,nm,\ b_{2,AP} = 600\,nm,\ b_{PV} = 2,\ b_{API} = 0.046\ a.u.$$

$$a_{FAP} = 0.25,\ a_{PV} = 0.5,\ a_{FAPI} = 0.25 \qquad \textbf{(4)}$$

$Y_{AP:i,j}$ is defined so that all first absorption peaks that shift below the starting peak wavelength are treated as positive changes equal to the magnitude of the negative change. The local reward (r_L) is then defined as the sum of the non-dimensional and weighted parameters:

$$r_L = Y_{AP} + Y_{API} + Y_{PV} \qquad \textbf{(5)}$$

r_L is then converted into a positive change local reward term (r_{LP}) which adds the improvement from the r_{LP} of the previous injection to the current r_{LP}:

$$r_{LP:i,j} = \begin{cases} r_{L:0,j} & if\ i = 0, \\ r_{LP:i-1,j} & if\ r_{L:i,j} < r_{LP:i-1,j} \\ r_{L:i,j} & if\ r_{L:i,j} \geq r_{LP:i-1,j} \end{cases} \qquad \textbf{(6)}$$

Finally, the r_{LP} is paired with the corresponding Y_{AP} values and fitted to a linear regression over the last eight injection steps to calculate the slope reward (r_S):

$$r_{S:i,j}(Y_{AP}, r_{LP}) = \begin{cases} 0 & if\ i = 0 \\ slope\left(\langle Y_{AP:0,j},\ Y_{AP:1,j}, \dots Y_{AP:i,j}\rangle,\ \langle r_{LP:0,j},\ r_{LP:1,j}, \dots r_{LP:i,j}\rangle\right) & if\ i < 8 \\ slope\left(\langle Y_{AP:i-8,j},\ Y_{AP:i-7,j}, \dots Y_{AP:i,j}\rangle,\ \langle r_{LP:i-8,j},\ r_{LP:i-7,j}, \dots r_{LP:i,j}\rangle\right) & if\ i \geq 8 \end{cases}$$

(7)

Note that terminal conditions, which do not have numeric values for Y_{AP}, Y_{PV}, and Y_{API}, are penalized and assigned an r_S value of -1. The slope reward function is designed to maximize the quality metrics y_{API} and y_{PV} and encourage increases in y_{AP} without overvaluing large increases. The local reward can provide a high value for conditions that cause a large y_{API} increase but irreparably reduce y_{API} and y_{PV}. The quality metrics can be retained at equivalent wavelengths through longer injection sequences. Additionally, the use of r_{LP} over r_L prevents the scenario where reward decreases produce higher slope rewards at later injections. The final adjusted reward value (r) is formed by first calculating the change r_S relative to the prior reward (Δr_S):

$$\Delta r_S = r_{S:i,j} - r_{S:i-1,j} \qquad \textbf{(8)}$$

then performing a Yeo-Johnson power transform on Δr_S.

Reinforcement Learning Algorithms – Sequence Selection

The reinforcement learning algorithm follows a traditional RL structure, shown in Supplementary Figure 29. For every action (*i.e.*, injection condition) selected by the algorithm, a new state and reward are sent from the environment (the droplet reactor) to the RL agent. Two

variants of the algorithm were applied at different stages of this study. The first method was used to identify the injection sequence with fixed volumes and reaction times, and the second method was used to optimize the injection volumes and reaction times after fixing the injection sequence.

Data Formatting for Belief Model Training – In the sequence selection studies, the action (A) at step t is defined solely as the injecting reagent:

$$A_t = I_t \qquad \textbf{(9)}$$

Where I_i is a one hot encoded representation of the injecting reagent or starting CdSe, i.e.:

$$I_t^{[CdSe]} = \langle 0,0,0,0 \rangle \qquad \textbf{(10)}$$

$$I_t^{[OAm]} = \langle 1,0,0,0 \rangle \qquad \textbf{(11A)}$$

$$I_t^{[Na_2S]} = \langle 0,1,0,0 \rangle \qquad \textbf{(11B)}$$

$$I_t^{[Cd(Ac)_2]} = \langle 0,0,1,0 \rangle \qquad \textbf{(11C)}$$

$$I_t^{[FAm]} = \langle 0,0,0,1 \rangle \qquad \textbf{(11D)}$$

Note that when the memory extends beyond the start of the droplet, the corresponding steps are encoded as $\langle 0,0,0,0 \rangle$.The state (S_t) is then defined as the short-term memory (STM_t) of the most recent prior actions up to memory length (M):

$$S_t = STM_t = \langle A_{t-M}, A_{t-M+1}, \dots A_{t-1} \rangle \qquad \textbf{(12)}$$

Where M is equal to 3.

Belief Model Training – The same belief model structure was used for the sequence selection experiments and the volume-time optimization experiments. The belief models consist of an ensemble deep neural network regressor (R) and a gradient boosted decision tree classifier (C). The regressor ensemble members (ε) are constructed using the Scikit-Learn (Version 1.0.2) multi-layer perceptron regressor function with an adaptive learning rate and a limited-memory Broyden–Fletcher–Goldfarb–Shanno solver.[1] Hidden layers in each neural network were constructed by randomly selecting the number of nodes in each of three layers. The number of nodes in the first, second, and third hidden layers were selected from ranges of 100 to 200, 50 to 100, and 20 to 50 respectively. Default values were used for all other parameters.

The regressor models are trained to map S_t and A_t to the adjusted reward at timestep t (r_t). Each model is trained on a 75% subsampling of the full available data set. Note that if insufficient data is available for subsampling, the full data set is used. The classifier was built using the default Scikit-Learn (Version 1.0.2) gradient boosting classifier function. The classifier was trained by mapping S_t and A_t to a binary representation of either terminal or non-terminal conditions. The full data set was used for classifier training.

Roll-Out Policy – The rollout policy uses the regressor and classifier models with forward projections of different injection sequences to evaluate the most favorable action from the current state. The forward mapped action matrix (A') is a N_{Branch} long list of injection sequences each of length N_{Level} that propose actions to be taken in series from the current state. The branches of A' consist of every permutation with replacement of the four possible injections for four levels ($N_{Level} = 4$), so $N_{Branch} = 256$. A' is represented as:

$$A' = \begin{bmatrix} A_{t+1}^{[1]} & \cdots & A_{t+N_{Level}}^{[1]} \\ \vdots & \ddots & \vdots \\ A_{t+1}^{[N_{Branch}]} & \cdots & A_{t+N_{Level}}^{[N_{Branch}]} \end{bmatrix} \quad \textbf{(13)}$$

The state forward matrix (S') is built using the current state and A':

$$S' = \begin{bmatrix} S_{t+1}^{[1]} & \cdots & S_{t+N_{Level}}^{[1]} \\ \vdots & \ddots & \vdots \\ S_{t+1}^{[N_{Branch}]} & \cdots & S_{t+N_{Level}}^{[N_{Branch}]} \end{bmatrix} \quad \textbf{(14)}$$

Where the state at forward mapping level a and branch b ($S_a^{[b]}$) is equal to the most recent relative injection sequence:

$$S_{t+a}^{[b]} = STM_{t+a}^{[b]} = \langle A_{t+a-M}^{[b]}, A_{t+a-M+1}^{[b]}, \dots A_{t+a-1}^{[b]} \rangle = \langle I_{t+a-M}^{[b]}, I_{t+a-M+1}^{[b]}, \dots I_{t+a-1}^{[b]} \rangle \quad \textbf{(15)}$$

The reward prediction forward matrix (r') is formed by randomly sampling from the regressor ensemble members for twenty duplicates ($D = 20$) and applying the cumulative probability from the classifier for the correspond state action pair to get a duplicate (d) matrix set ($r''(d)$):

$$r''(d) = \begin{bmatrix} r_{t+1}^{[1]} & \cdots & r_{t+N_{Level}}^{[1]} \\ \vdots & \ddots & \vdots \\ r_{t+1}^{[N_{Branch}]} & \cdots & r_{t+N_{Level}}^{[N_{Branch}]} \end{bmatrix}_d \quad \textbf{(16)}$$

$$r_{t+a}^{[b]} = P_{t+a}^{[b]} \, \varepsilon(S_{t+a}^{[b]}, A_{t+a}^{[b]}) \quad \textbf{(17)}$$

Where $P_{t+a}^{[b]}$ is defined as a function of the classifier probability predictions ($p_{t+a}^{[b]}$):

$$p_{t+a}^{[b]} = p_{t+a-1}^{[b]} \, C\left(S_{t+a}^{[b]}, A_{t+a}^{[b]}\right) \quad \textbf{(18)}$$

$$p_t^{[b]} = 1$$

$$P_{t+a}^{[b]} = \begin{cases} 0 \; if \; p_{t+a-1}^{[b]} C\left(S_{t+a}^{[b]}, A_{t+a}^{[b]}\right) \leq 0.3 \\ 1 \; if \; p_{t+a-1}^{[b]} C\left(S_{t+a}^{[b]}, A_{t+a}^{[b]}\right) > 0.3 \end{cases} \quad \textbf{(19)}$$

The final r' is formed by taking the average across duplicates:

$$r' = \sum_{k=1}^{D} \frac{r''(k)}{D} \quad \textbf{(20)}$$

And the reward uncertainty matrix (r'_{Uncert}) is created by taking the standard deviation across duplicates:

$$r'_{Uncert} = \sqrt{\sum_{k=1}^{D} \frac{\left(r' - r''(k)\right)^2}{D-1}} \quad \textbf{(21)}$$

Next, the value for each injection option I (Q_I) is calculated by first taking the upper confidence bounds (UCB) of the reward and uncertainty terms to create a value matrix (q'):

$$q' = r' + \lambda \, r'_{Uncert} \quad \textbf{(22)}$$

Where λ is the exploration weight constant. The branch value ($q^{[b]}$) is then calculated by taking the maximum among all estimated level values for each branch:

$$q^{[b]} = \max\left(\langle q_{t+1}^{[b]}, q_{t+2}^{[b]}, \ldots q_{t+N_{Level}}^{[b]} \rangle\right) \quad \textbf{(23)}$$

Finally, $q^{[b]}$ is grouped by the first injection in the forward prediction sequences, and Q_I is calculated by averaging over the highest 25% of values in each of the groups.

The entropy term (N_I), based on the UCB1 algorithm,[33] is calculated by comparing the total number of injections saved in the training data set (n_T) to the number of a specific injection (n_I):

$$N_I = \sqrt{\frac{2 \log(n_T + 1)}{n_I + 1}} \quad \textbf{(24)}$$

The final recommended action ($A_{t+1,Rec}$) is the injection that produces the maximum value for the sum of the value and entropy terms:

$$A_{t+1,Rec} = I_{t+1,Rec} = argmax(Q_I + N_I) \quad \textbf{(25)}$$

Reinforcement Learning Algorithms – Volume and Time Optimization

Data Formatting for Belief Model Training – The algorithms used for the volume-time optimization campaigns are similar in structure to the sequence optimization, but several modifications are necessary to accommodate the continuous parameters. For these experiments, A_t is defined as a function of the non-dimensional injection volume (V_t) and time (T_t) parameters:

$$A_t = \langle V_t, T_t \rangle \quad \textbf{(26)}$$

STM_t is again defined as the most recent sequence of actions up to M steps back:

$$STM_t = \langle A_{t-M}, A_{t-M+1}, \dots A_{t-1} \rangle \quad \textbf{(27)}$$

S_t includes STM_t along with the added terms cycle number (n_C) and injection step per cycle (n_I):

$$S_t = \langle n_{C,t}, n_{I,t}, STM_t \rangle \quad \textbf{(28)}$$

Where n_C is an integer value corresponding to the full cycle count for a single droplet, and n_I is a one-hot encoded representation of the injection number per cycle for the five consecutive injections in each cycle.

Belief Model Training – Belief models are training using the same methods detailed in the sequence selection algorithm with no changes, except for the size of the input layers.

Roll-Out Policy – Forward mapping and estimation of the reward and uncertainty is conducted using a different approach than the sequence selection algorithm. First, the volume and time parameters are given discrete value representations (V^{Desc} and T^{Desc} respectively) through a seven level (n_{DLevel}), uniform discretization across the full ranges of continuous values such that:

$$V^{Desc} = floor(V\, n_{DLevel}) \quad \textbf{(29A)}$$

$$T^{Desc} = floor(T\, n_{DLevel}) \quad \textbf{(29B)}$$

The first layer of the forward mapped action matrix (A') is formed by creating an array of every combination of possible discrete values between the two parameters to form an n_{DLevel}^2 long list of next possible actions. Each branch in A' is built by randomly sampling a continuous value from the ranges specified by inversion of V^{Desc} and T^{Desc} for each discrete value pair. The remaining actions in each branch of A' are built by randomly selecting values across the full continuous spaces of V and T for four total levels. This process is repeated 100 times for all branches in the action matrix, using new random values for all levels. The reward prediction forward matrix (r'') is built using the same methods described previously, where the classifier and regressor are iteratively sampled for each level in each branch. The value of branch b for duplicate d ($q_d^{[b]}$) is then defined as the maximum value of the cumulative sum of reward for each branch and duplicate ($r_{CSum,i}^{[b,d]}$):

$$r_{CSum,i}^{[b,d]} = \sum_{k=1}^{i} r_k^{[b,d]} \quad \textbf{(30)}$$

$$q_d^{[b]} = \max\left(\langle r_{CSum,t+1}^{[b,d]}, r_{CSum,t+2}^{[b,d]}, \dots r_{CSum,t+NLevel}^{[b,d]} \rangle\right) \quad \textbf{(31)}$$

The mean value and value uncertainty for a given branch ($q^{[b]}$ and $q_{Uncert}^{[b]}$ respectively) are formed by taking the mean and standard deviation, respectively, across all value duplicates:

$$q^{[b]} = \sum_{k=1}^{D} \frac{q_k^{[b]}}{D} \quad \textbf{(32)}$$

$$q_{Uncert}^{[b]} = \sqrt{\sum_{k=1}^{D} \frac{\left(q^{[b]} - q_k^{[b]}\right)^2}{D-1}} \quad \textbf{(33)}$$

V_{t+1}^{Desc} and T_{t+1}^{Desc} are then selected using the UCB of the value terms to identify the branch corresponding to the most promising discrete volume-time pair (b_{rec}):

$$b_{rec} = argmax(q^{[b]} + \lambda\, q_{Uncert}^{[b]}) \quad \textbf{(34)}$$

The final V_{t+1} and T_{t+1} are selected by randomly sampling from the ranges indicated by the discrete volume-time pair.

Digital Twin Studies

Digital Twin Structure – The digital twin is composed of four models: the viability classifier, change in absorption peak wavelength regressor, absorption peak intensity regressor, and peak to valley ratio regressor – shown in Supplementary Figure 30. The viability classifier uses the same structure used in the RL belief model. All three regressors use the same ensemble neural network structure as the RL belief model with the following modifications: The absorption peak wavelength, absorption peak intensity, and peak to valley regressors used a 10%, 10%, and 75% subsampling rate respectively. All regressors had an ensemble size of 200, erroneous data not caught by the automated processing scripts was filtered out, and the ensemble mean prediction uses data trimming for all predictions outside one standard deviation from the median.

Bayesian Optimization Algorithm – The BO algorithm used in the digital twin study follows the same design implemented in prior work.[2] The belief model is a 20-member ensemble neural network with the same structure of that used in the RL belief model. The algorithm uses a UCB decision policy with the predicted value (q_{UCB}) defined as:

$$q_{UCB} = \mu_{rL} + \frac{1}{\sqrt{2}}\sigma_{rL} \quad \textbf{(35)}$$

Where μ_{rL} is the mean predicted reward for a set of input conditions and σ_{rL} is the standard deviation of the prediction. The belief model was trained on local reward after all 20 injection conditions are applied (*i.e.*, 40 total input parameters).

Supplementary Figures and Tables

Supplementary Table 1 – Function Instruction Format

Function	Func. #	Property 1	Property 2	Property 3	Property 4
Initial Injection	0	Injection Pump Number (#)	Injection Volume (µL)	Injection Flow Rate (µL/min)	-
Inject Precursor	1	Phase Sensor/Injection Pump Number (#)	Injection Volume (µL)	Injection Flow Rate (µL/min)	Delay Time (ms)
Oscillate Droplet	2	Number of Oscillations (#)	Oscillation Flow Rate (µL/min)	-	-
Optical Spectra	3	Number of Spectra*Flow Rate (#*(mL/min))	Carrier Flow Rate (µL/min)	Absorption Integration Time (ms)	Photoluminescence Integration Time (ms)
Waste Droplet	4	Line Clear Time (s)	Injection Line Flush Yes (1) or No (0)	Solvent Flush Yes (1) or No (0)	-
Refill Syringes	5	Refill Flow Rate (µL/min)	Flush Out Volume (µL)	-	-
Separate Phase	6	Approach Flow Rate (µL/min)	Reverse Delay (ms)	Forward Flow Rate (µL/min)	Split Flow Rate (µL/min)

Supplementary Table 2 – Optimized Volume-Time Conditions

		480 nm		*520 nm*		*560 nm*	
**Cycle Number**	**Injecting Precursor**	**Volume (µL)**	**Time (s)**	**Volume (µL)**	**Time (s)**	**Volume (µL)**	**Time (s)**
0	*Starting QD*	10	-	10	-	10	-
1	*OAm*	5.5	440	7.6	306	8.1	218
1	*Na_2S*	4.1	395	7.3	351	1.6	173
1	*FAm*	2.0	262	8.0	262	4.1	395
1	*$CdAc_2$*	9.5	84	5.7	262	1.0	129
1	*OAm*	7.5	351	5.1	218	2.7	395
2	*OAm*	6.5	306	3.0	84	2.2	306
2	*Na_2S*	3.5	173	2.9	351	6.1	173
2	*FAm*	4.7	218	5.4	173	8.7	218
2	*$CdAc_2$*	8.2	351	9.3	84	7.7	173
2	*OAm*	1.6	173	7.7	218	8.6	440
3	*OAm*	2.0	262	4.7	173	8.0	129
3	*Na_2S*	7.4	306	4.8	440	2.7	218
3	*FAm*	5.4	262	9.8	218	5.2	262
3	*$CdAc_2$*	7.2	84	5.3	262	6.9	395
3	*OAm*	8.1	173	5.4	129	8.4	351
4	*OAm*	7.9	129	3.4	306	2.0	351
4	*Na_2S*	5.9	173	7.7	129	6.4	395
4	*FAm*	8.8	173	1.1	129	8.3	173
4	*$CdAc_2$*	3.5	218	8.2	84	1.0	40
4	*OAm*	5.0	129	7.2	306	4.9	351

Supplementary Table 3 – Precision of Half-Cycles in Operational Window

	Tol Abs. @350 nm	Rel. Int. PL	First Abs Peak (nm)	HWHM	Peak / Valley	Tol Length (cm)
Mean	0.079	199464	485.9	0.088	1.90	2.74
Stdev.	0.0028	161295	0.97	0.0016	0.047	0.100

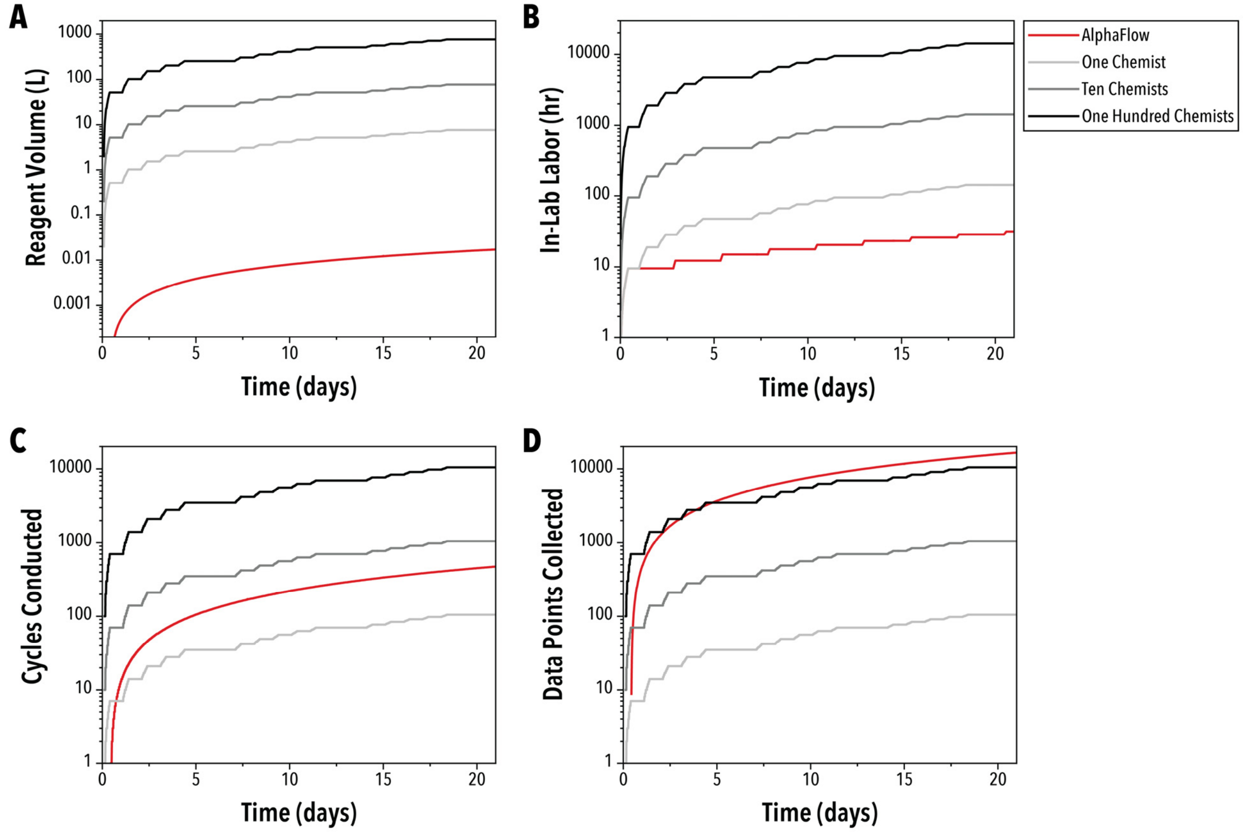

Supplementary Figure 1 – Material and Time Cost Summary. *Estimated (A) reagent volume consumed, (B) total in-lab labor requirement, (C) total cALD cycles conducted, and (D) total measurement collected as a function of time for AlphaFlow and different sets of human experimentalists.*

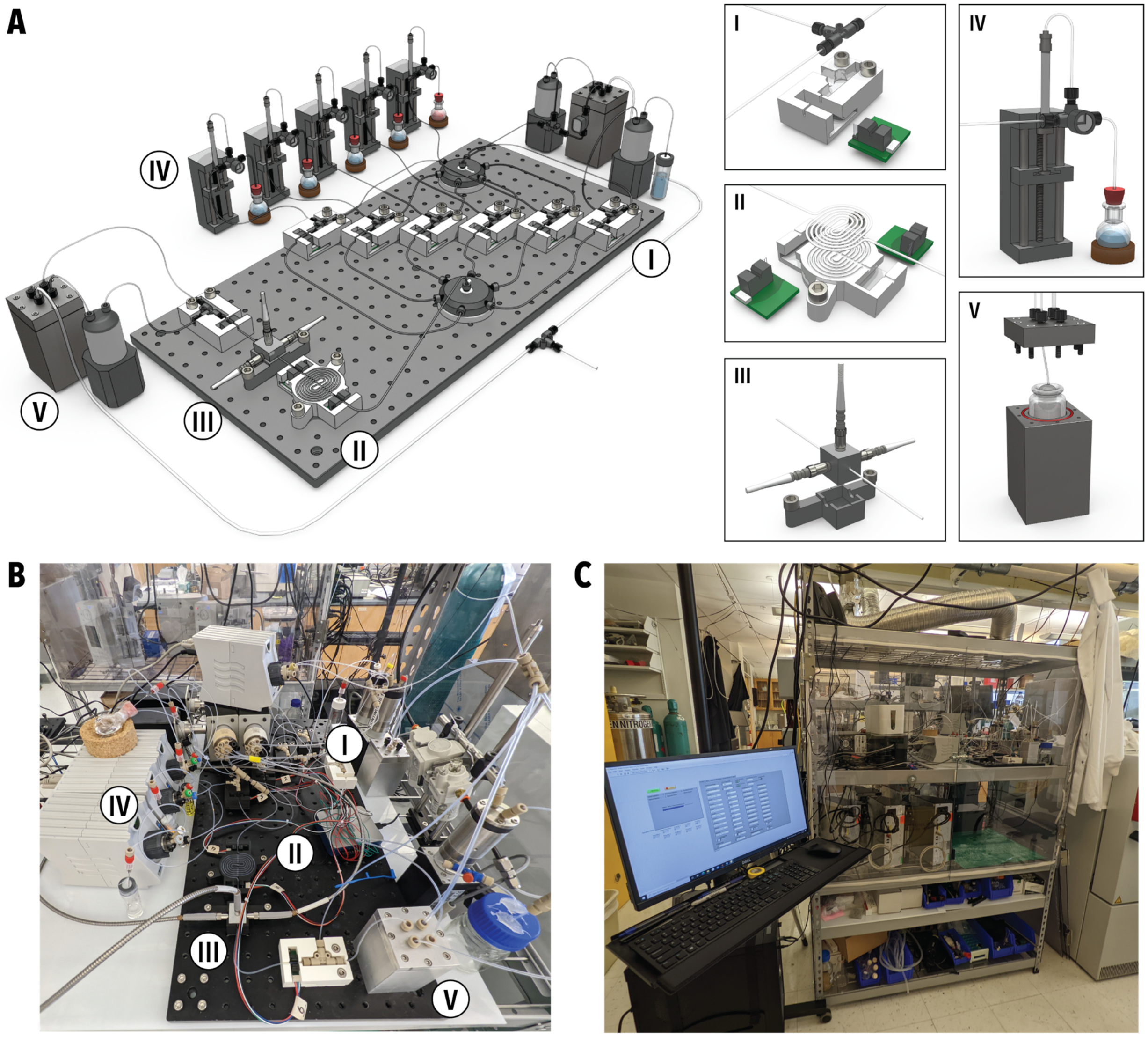

Supplementary Figure 2 – Microdroplet Reaction System. (A) *Schematic of full reactor system with (I) precursor injection and phase separation, (II) droplet oscillation, (III) optical sampling, (IV) syringe injection and refill, and (V) waste collection modules. Photograph of (B) the flow system and corresponding modules and (C) the full AlphaFlow setup.*

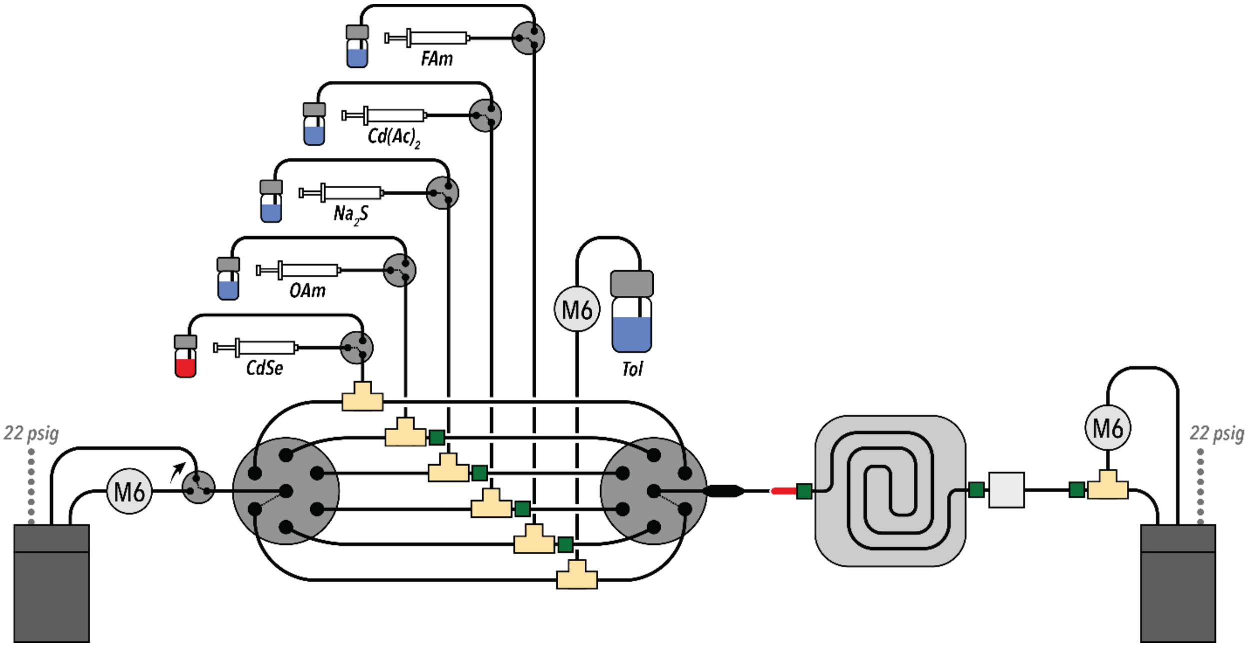

Supplementary Figure 3A – Selector Valve Position Change Step 1. *(1) The pressurization valve is switched to connect the upstream pressure vessel directly to the reactor, bypassing the continuous flow carrier pump.*

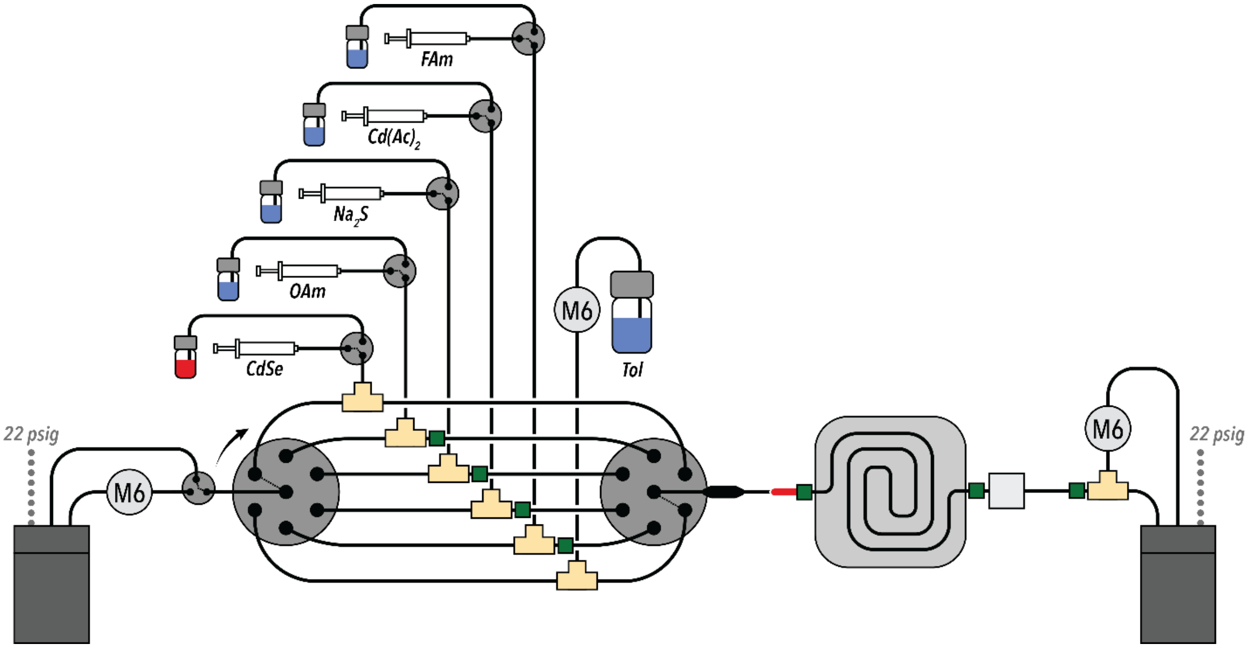

Supplementary Figure 3B – Selector Valve Position Change Step 2. *(2) The upstream selector valve is moved one increment towards the desired position.*

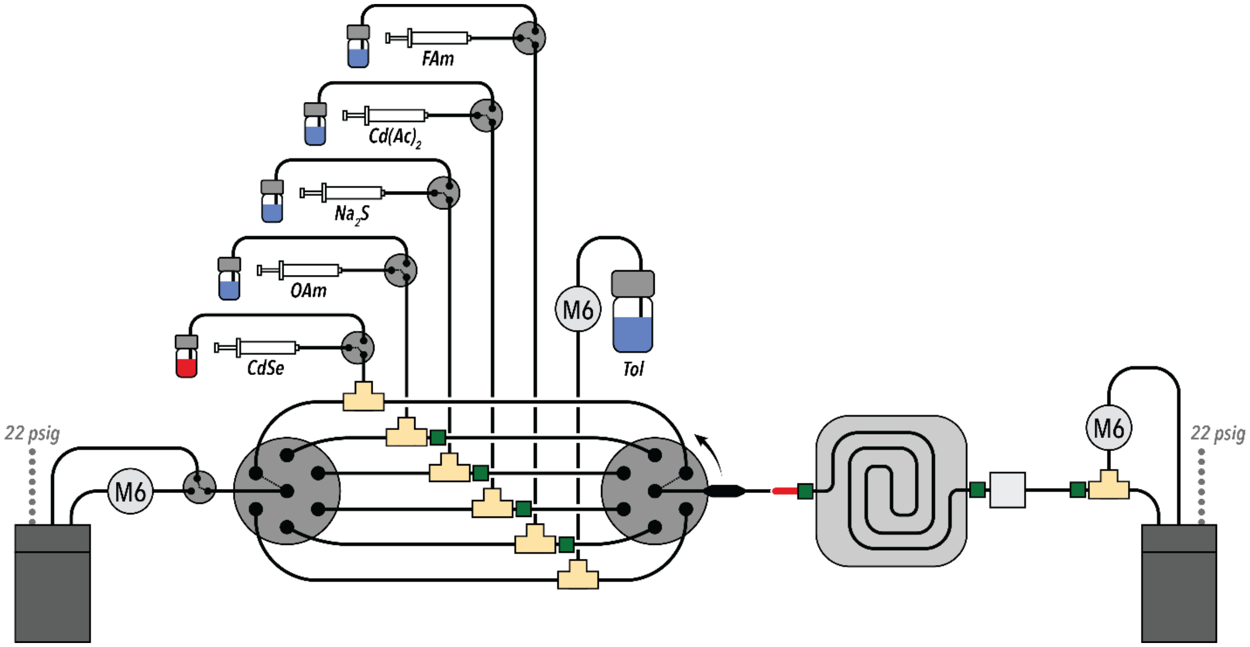

Supplementary Figure 3C – Selector Valve Position Change Step 3. *(3) The downstream selector valve is moved to match the upstream valve position.*

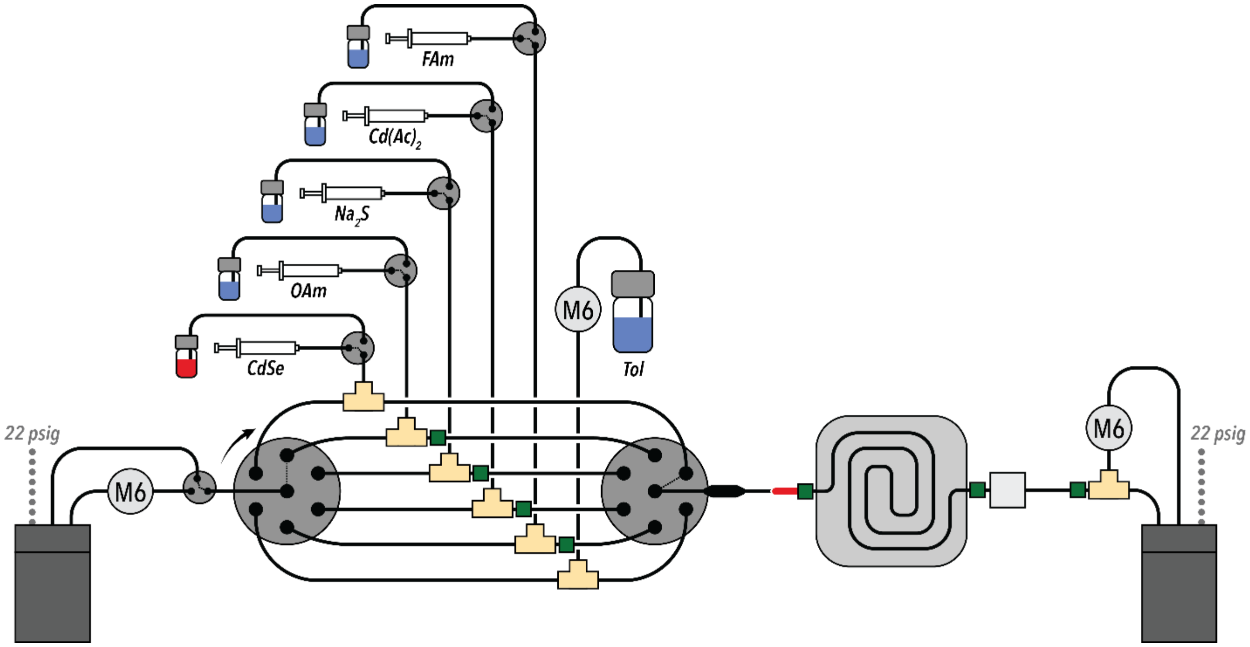

Figure 3D – Selector Valve Position Change Step 4. *(4) The upstream valve is again move one increment towards the target, which in this case is the OAm injection channel.*

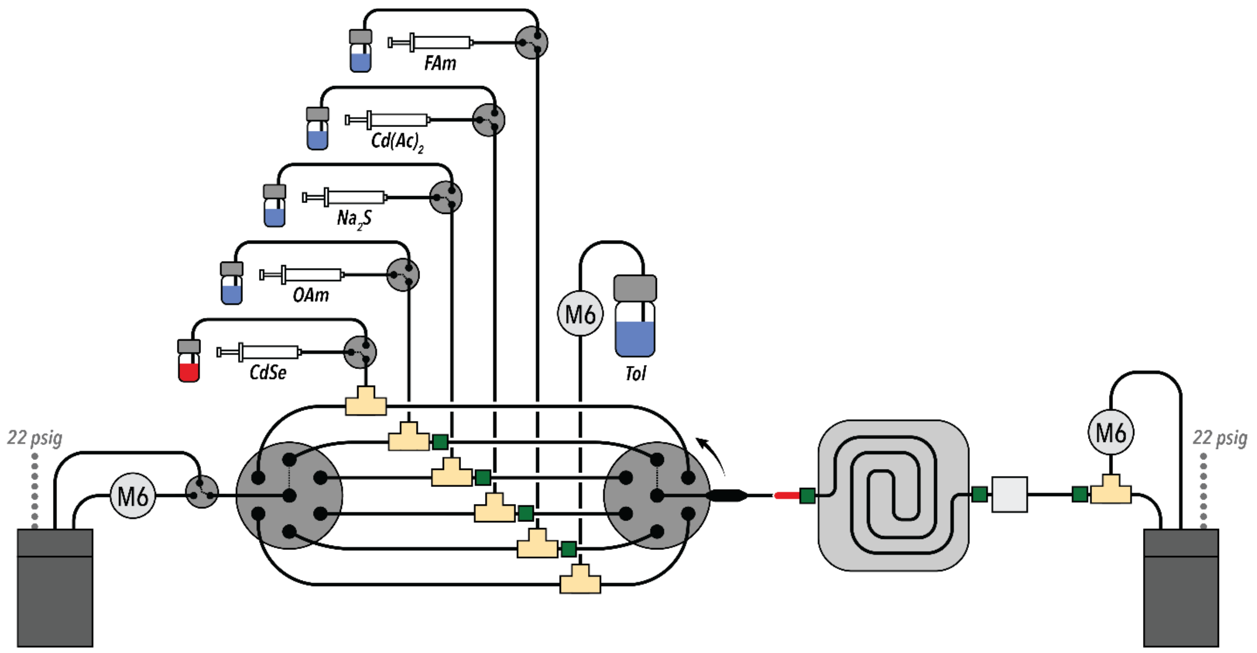

Supplementary Figure 3E – Selector Valve Position Change Step 5. *(5) The downstream selector valve is again moved to match the upstream valve position.*

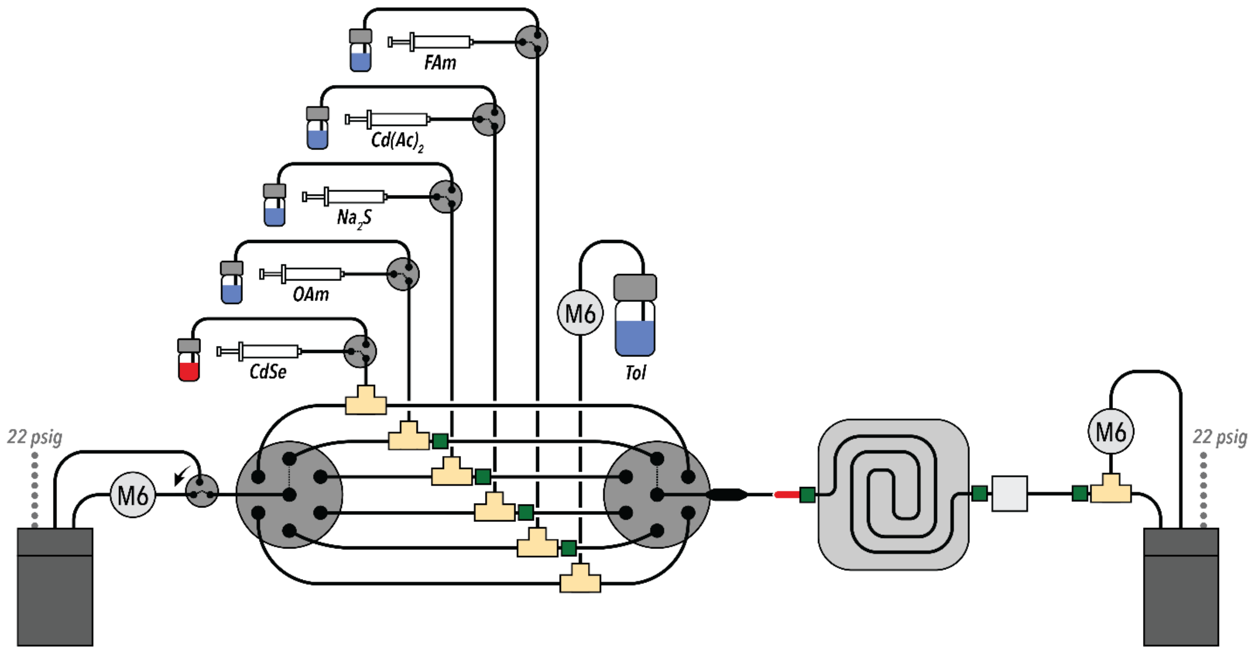

Supplementary Figure 3F – Selector Valve Position Change Step 6. *(6) The alternating position increments are repeated until the desired valve position is reached, then the pressurization valve switched to reconnect the continuous flow pump to the reactor.*

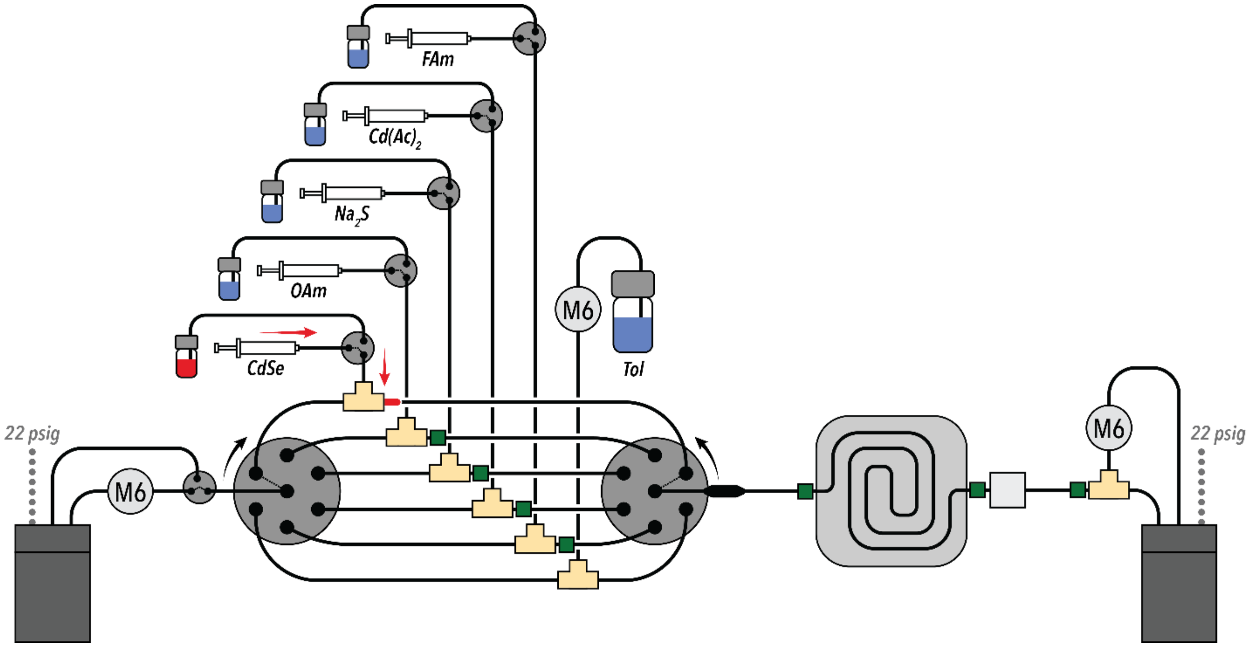

Supplementary Figure 4A – Initial Droplet Injection Steps 1-2. *(1) The selector valve position is changed to the desired injection channel, (2) then the corresponding syringe is set to inject the target volume.*

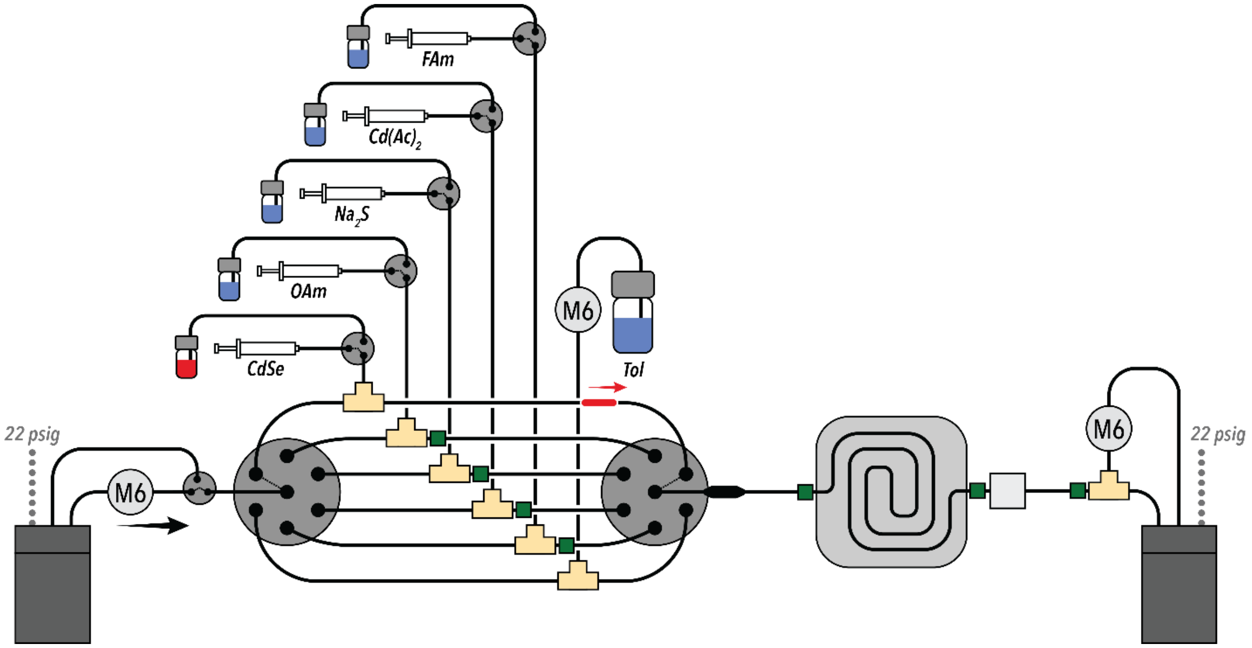

Supplementary Figure 4B – Initial Droplet Injection Step 3. *(3) The carrier flow pump is set to flow forward, pushing the droplet toward the start of the reactor spiral.*

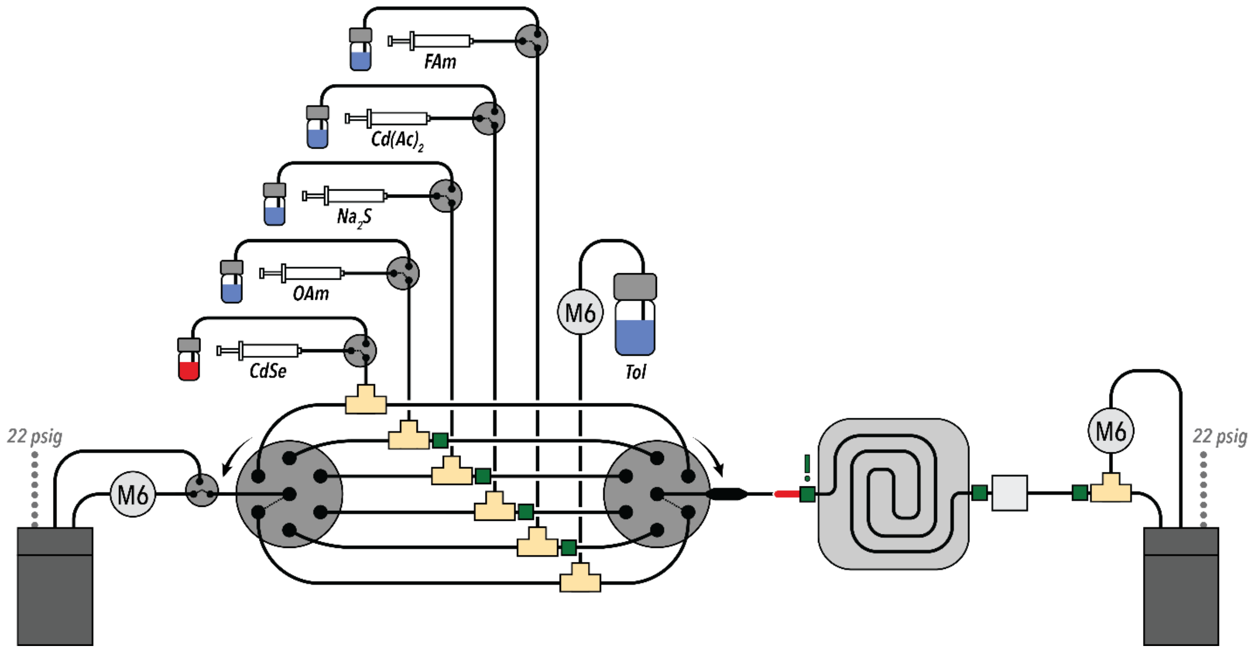

Supplementary Figure 4C – Initial Droplet Injection Steps 4-6. *(4) When the droplet is detected by the reactor spiral phase sensor, (5) the carrier flow is stopped, (6) then the valves are returned to their primary position – i.e. the toluene injection line.*

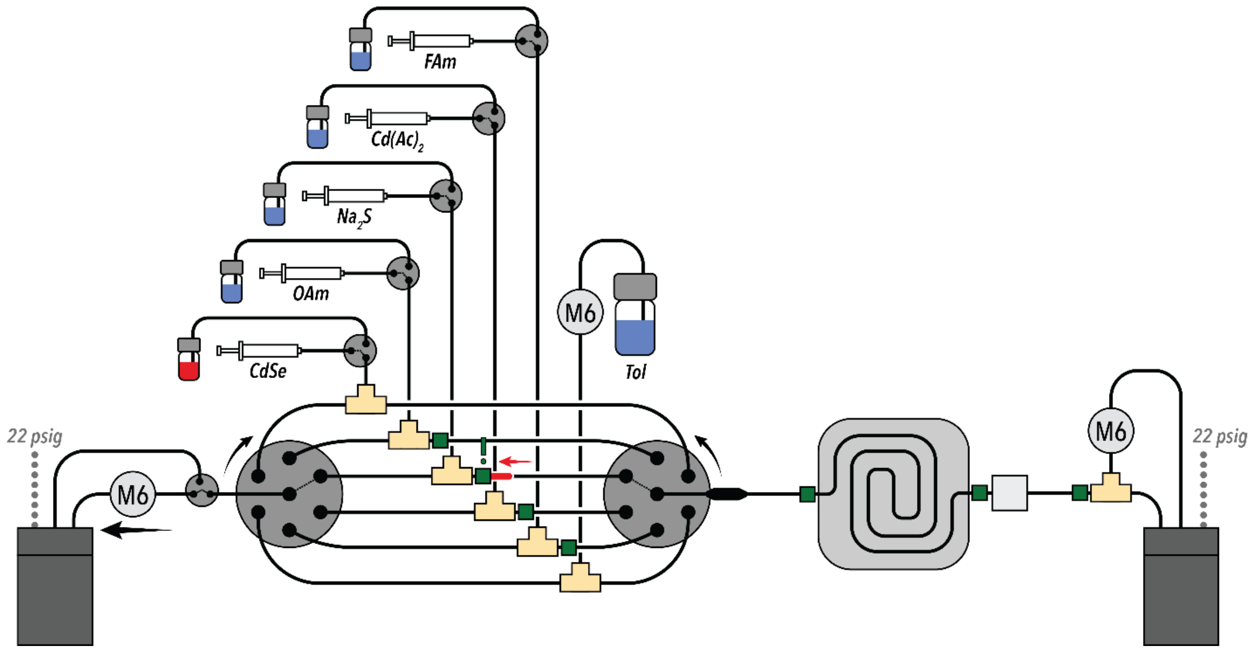

Supplementary Figure 5A – Precursor Injection Steps 1-3. *(1) The selector valves are set to the desired injection channel, and (2) the carrier flow is set to reverse until (3) the corresponding injection phase sensor detects the droplet.*

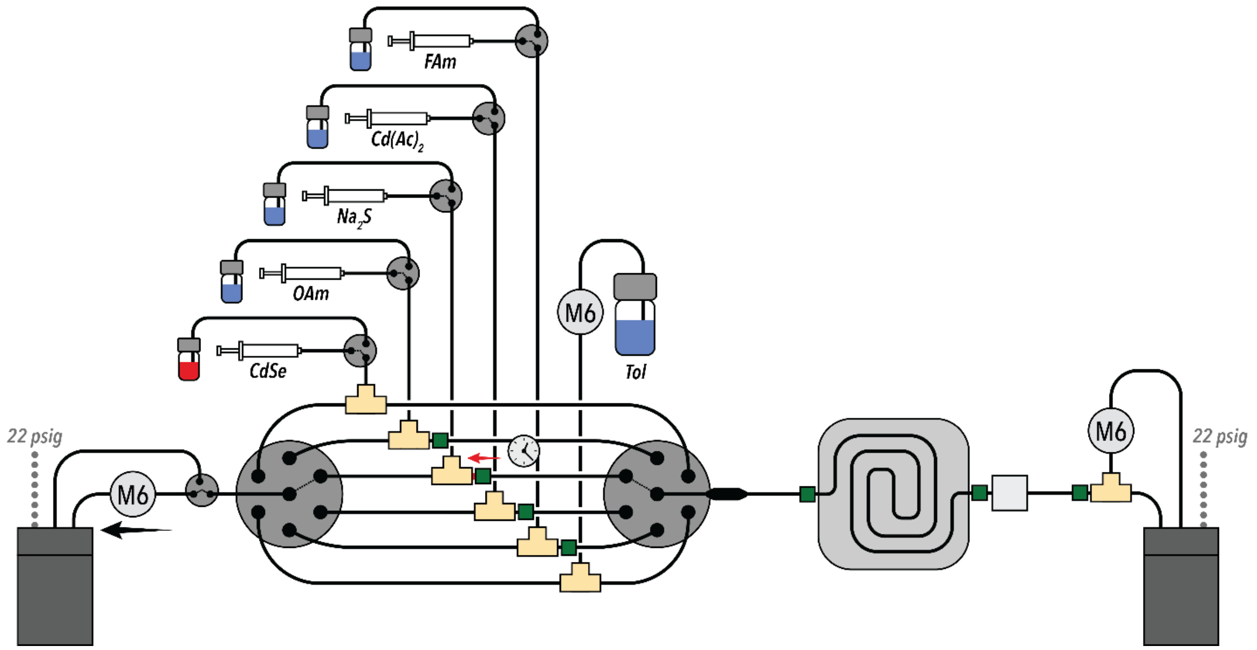

Supplementary Figure 5B – Precursor Injection Steps 4-5. *After the droplet is detected, (4) the reverse flow continues for the set injection delay time (1700 ms), (5) then the flow is stopped.*

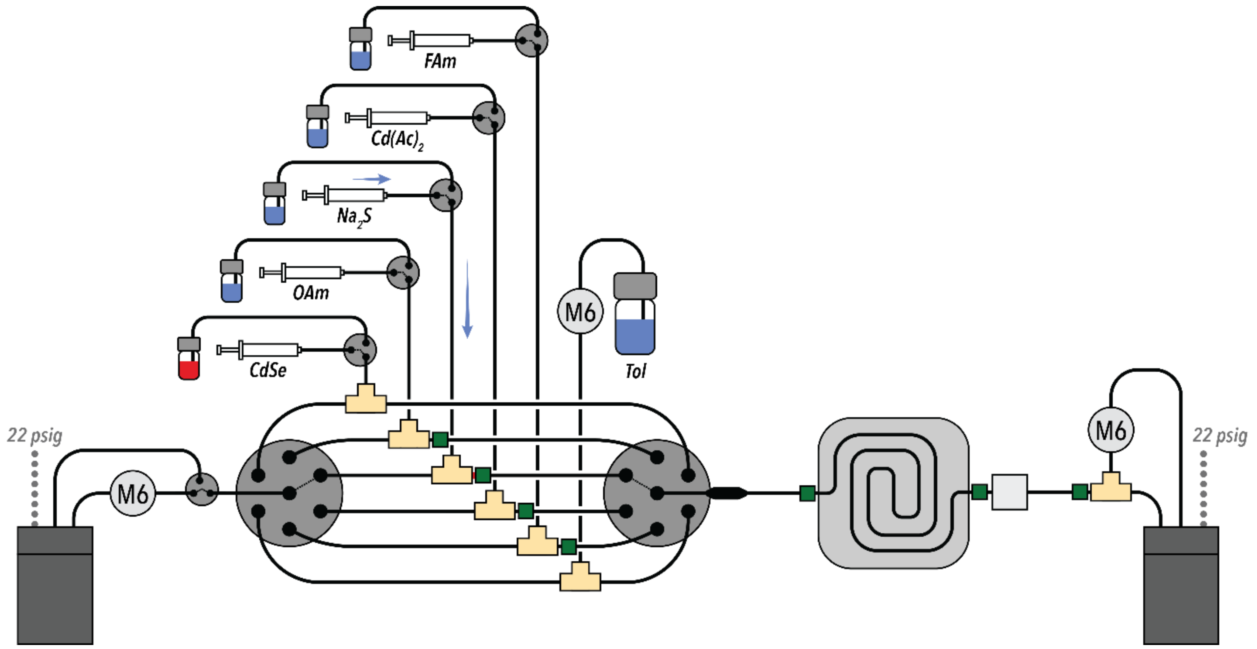

Supplementary Figure 5C – Precursor Injection Step 6. *(6) After the carrier flow is stopped, the injection syringe is set to inject the target volume into the droplet.*

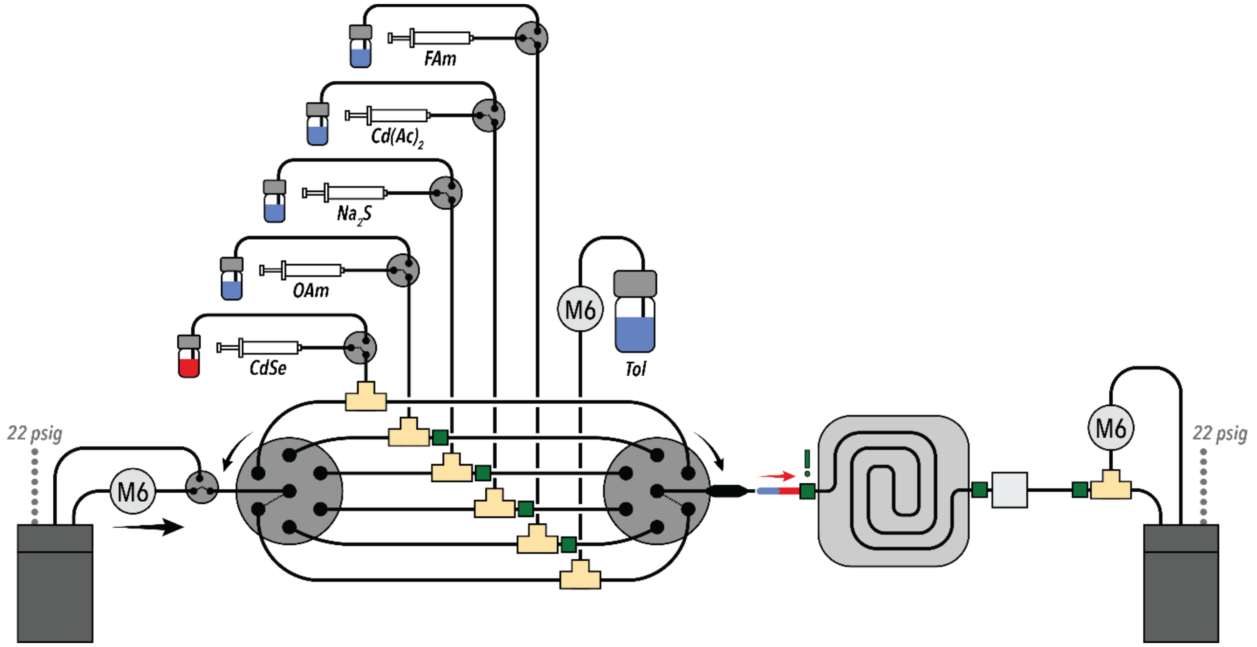

Supplementary Figure 5D – Precursor Injection Steps 7-9. *(7) The carrier flow is then set to forward until (8) the reactor spiral phase sensor detects the combined droplet, (9) then the selector valves are returned to their primary positions.*

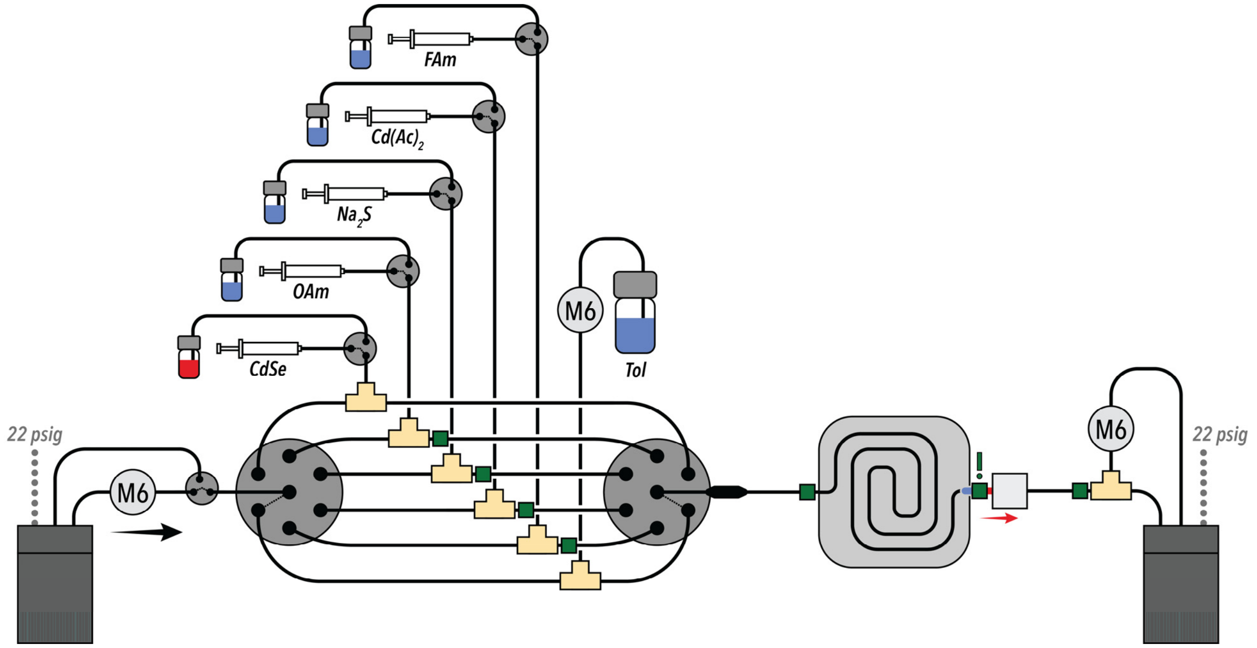

Supplementary Figure 6A – Optical Sampling Steps 1-2. *(1) The carrier pump is set to flow forward until (2) the flow cell phase sensor detects the droplet. This transit time coupled with the measured reactor spiral length is used to calculate the average droplet velocity.*

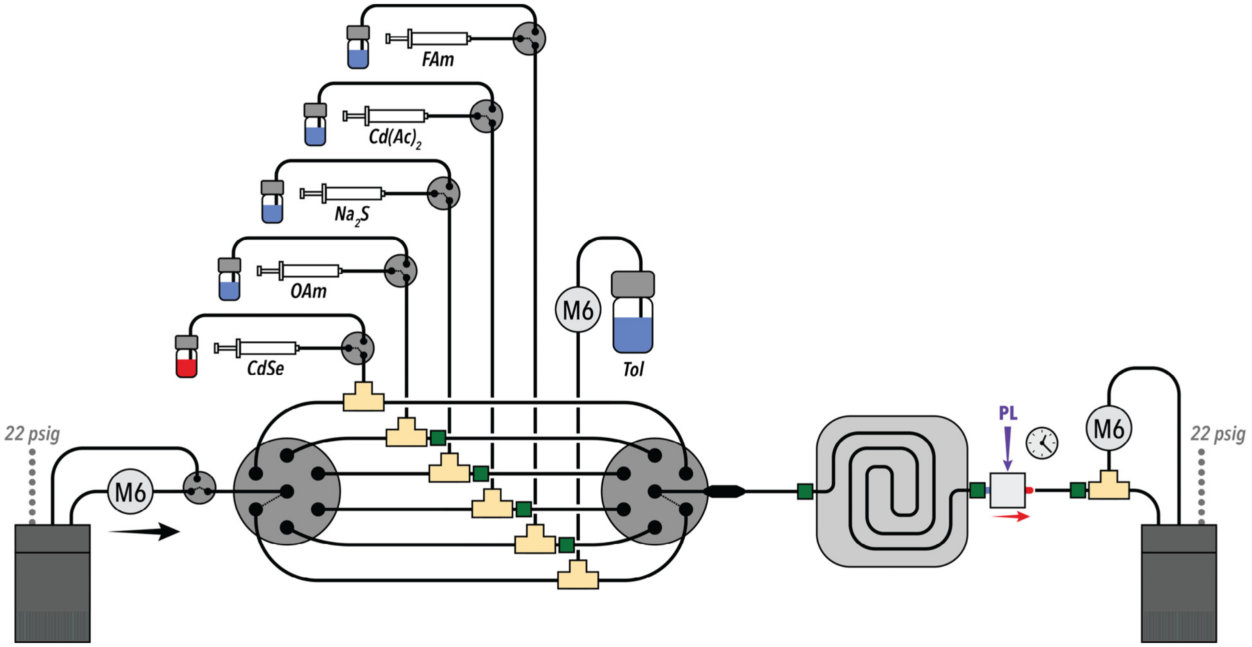

Supplementary Figure 6B – Optical Sampling Steps 3-4. *After detecting the droplet, (3) the photoluminescence excitation LED is turned on and (4) spectra are collected continuously for a set time duration.*

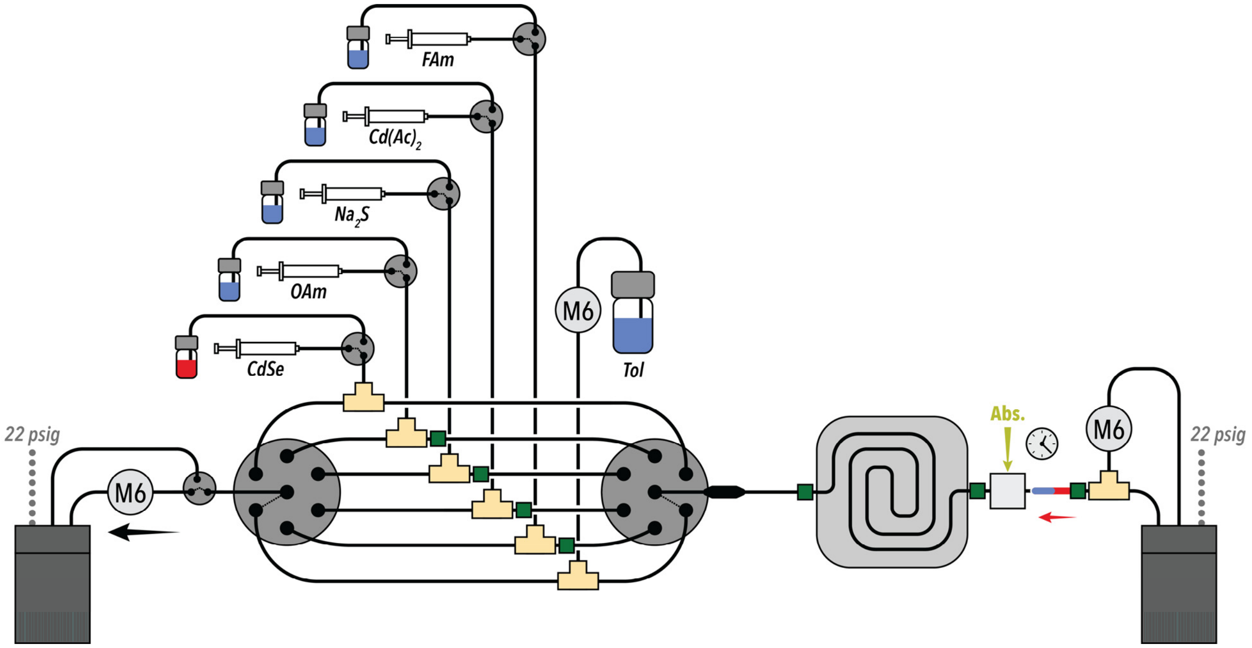

Supplementary Figure 6C – Optical Sampling Steps 5-7. *After the delay time, (5) the carrier flow is reversed, (6) the LED is switch off, the absorption light source is turned on, and (7) absorption spectra are collected continuously for a set time duration.*

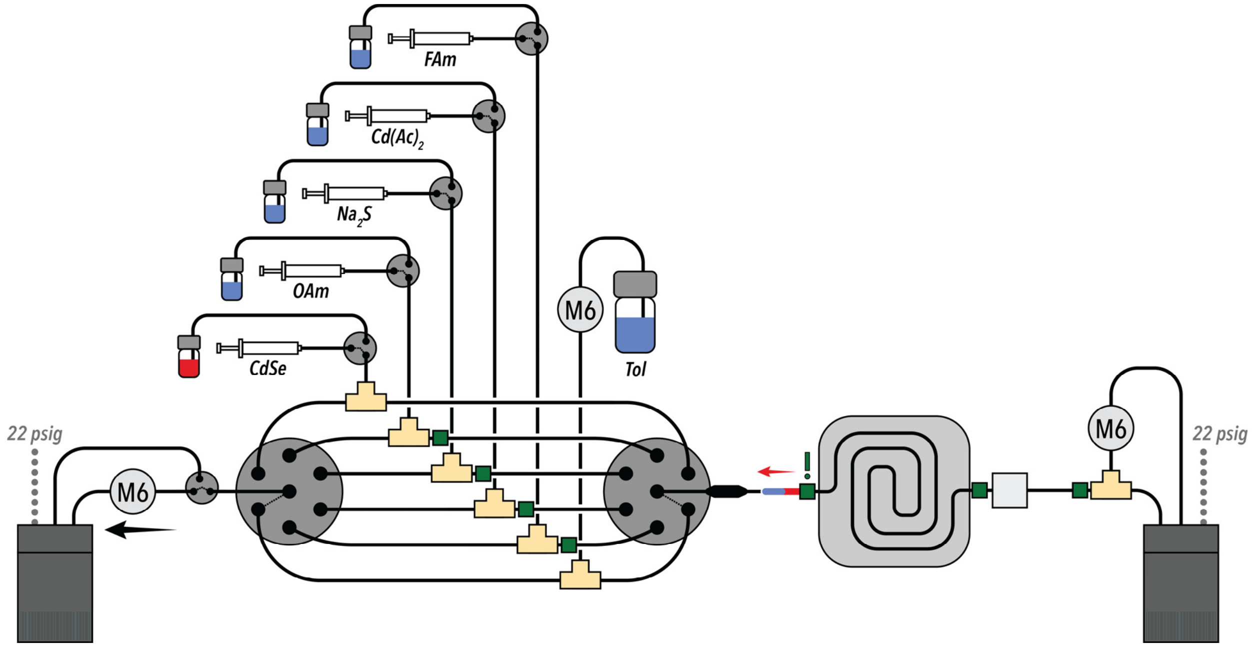

Supplementary Figure 6D – Optical Sampling Steps 8-9. *Reverse carrier flow is continued past the absorption sampling time until (8) the reactor spiral phase sensor detects the droplet, (9) at which point the carrier flow is stopped.*

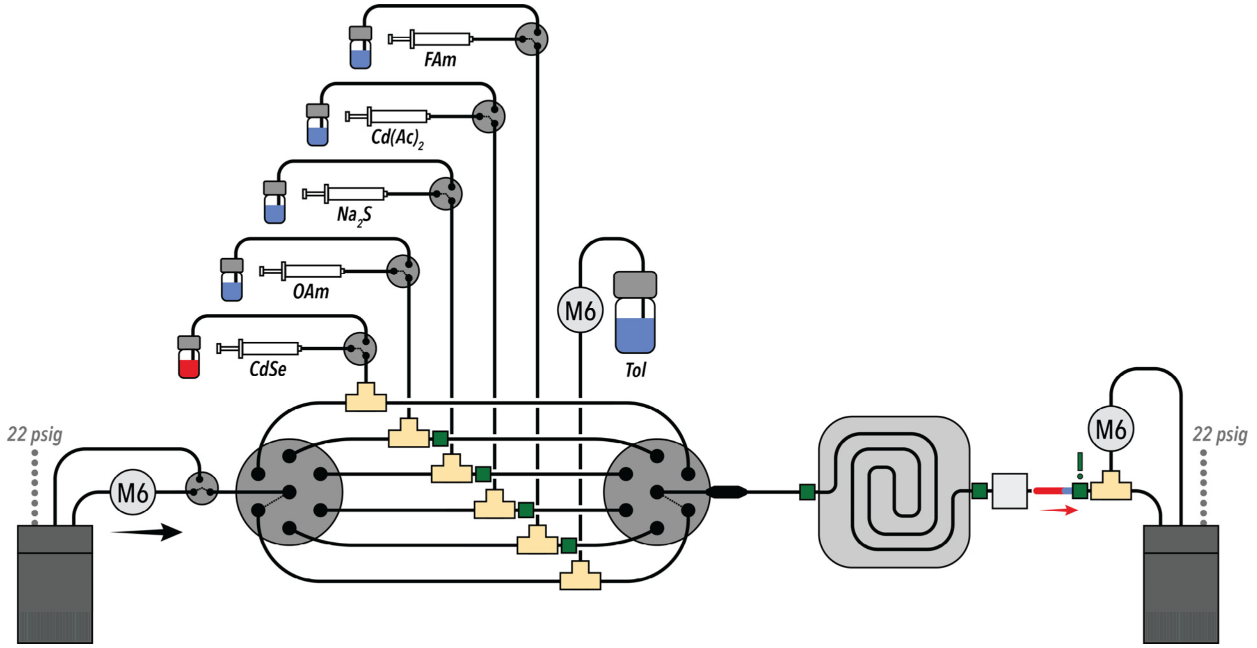

Supplementary Figure 7A – Phase Separation Steps 1-2. *(1) The carrier pump is set to forward flow until (2) the separator phase sensor detects the droplet.*

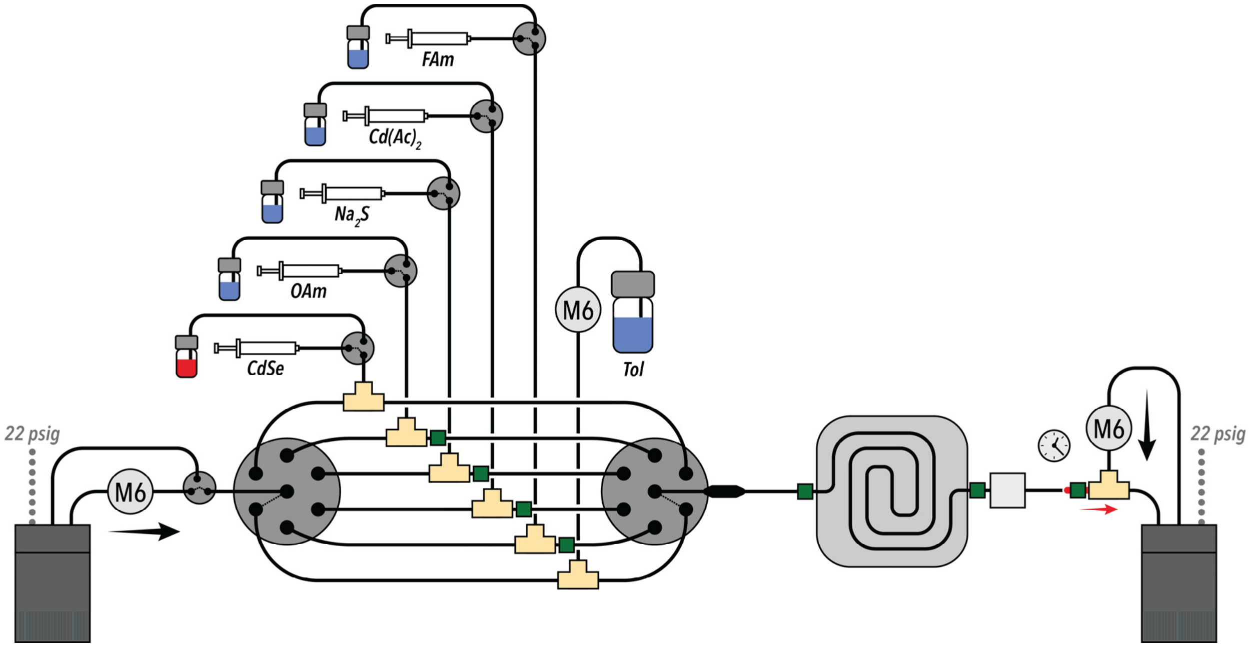

Supplementary Figure 7B – Phase Separation Steps 3-4. *After detection, (3) the separation carrier pump is set to flow forward, and (4) the primary carrier pump continues for a set delay time. This delay time is based on the calibration curve shown in SI Section S.X, and the corresponding formamide length is based on the measurements made in the most recent optical sampling step.*

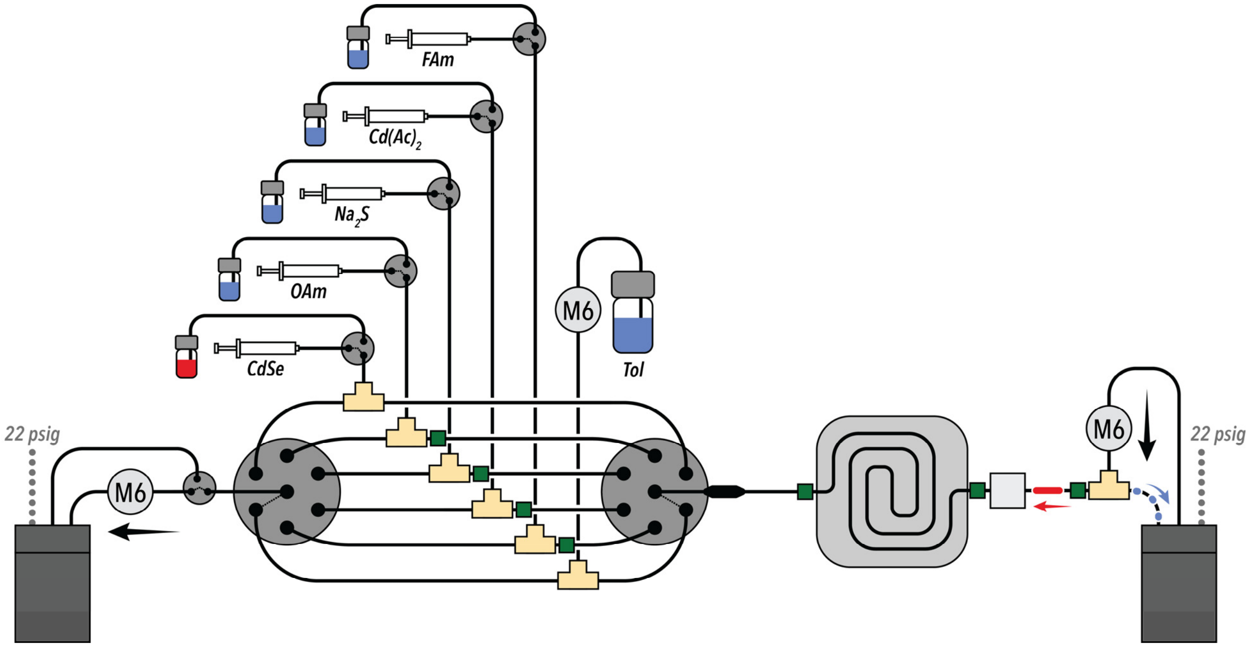

Supplementary Figure 7C – Phase Separation Step 5. *After the delay time, (5) the primary carrier pump is set to reverse.*

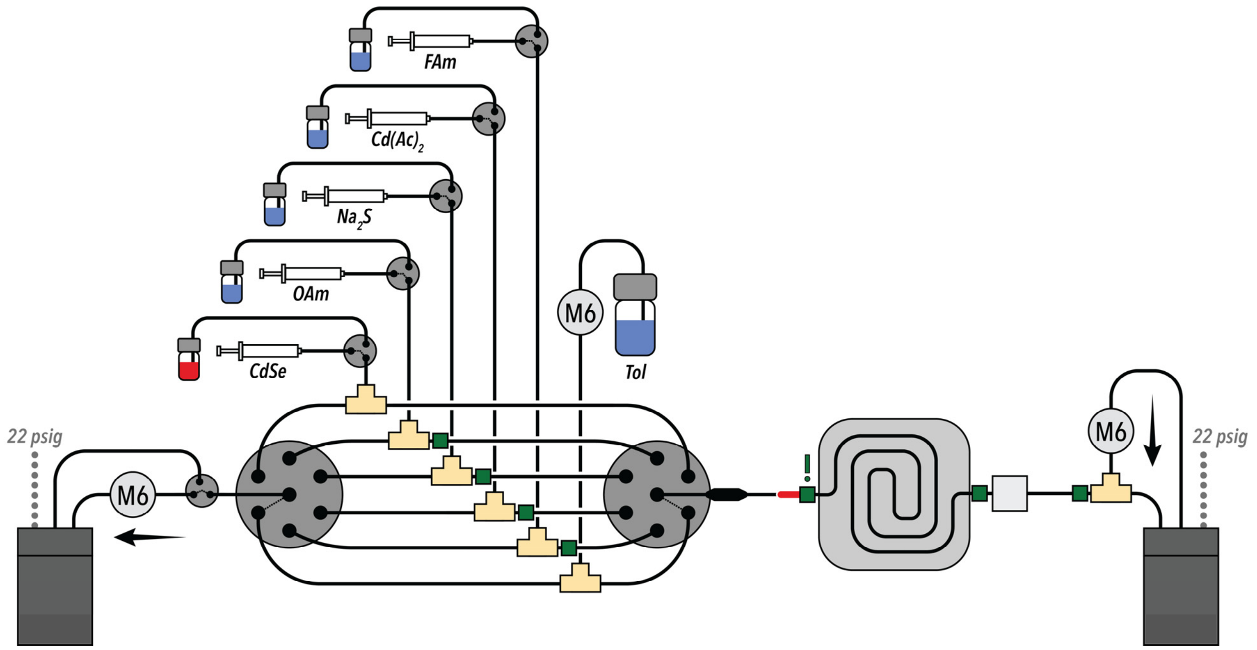

Supplementary Figure 7D – Phase Separation Steps 6-8. *(6) The separator carrier pump is stopped after 30 sec to ensure that the formamide phase has been fully transferred to the waste valve, and (7) when the reactor spiral phase sensor detects the droplet (8) the primary carrier pump is stopped.*

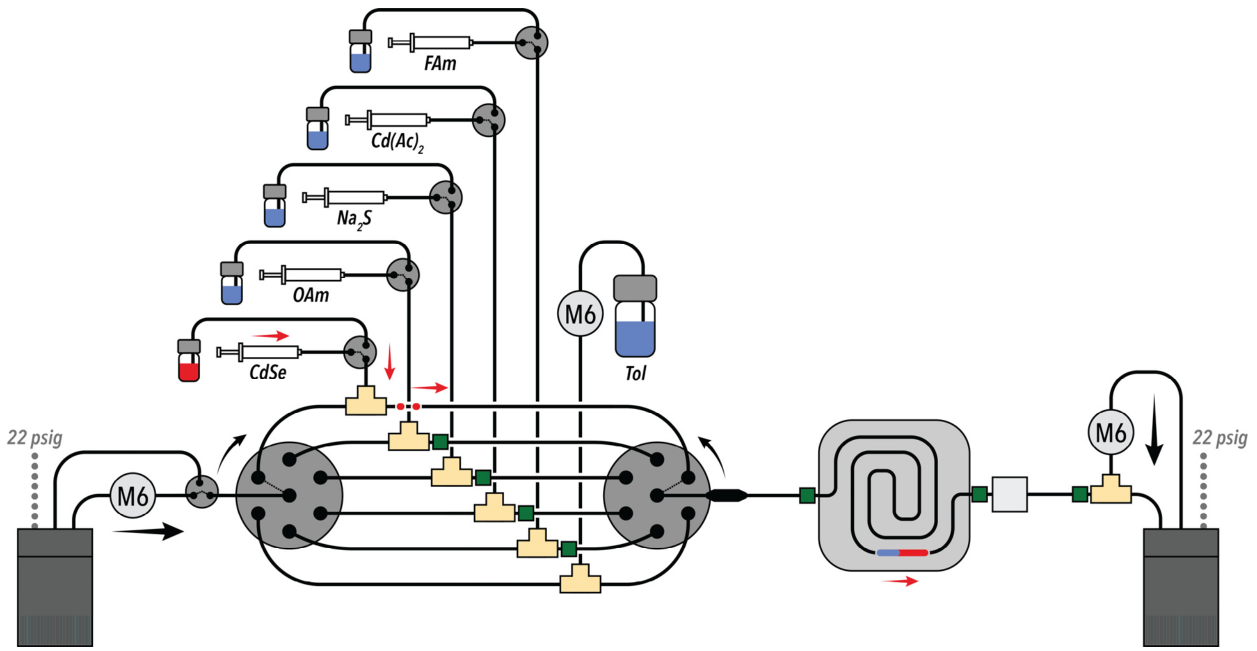

Supplementary Figure 8A – Waste and Cleaning Steps 1-3. *(1) Both carrier pumps are set to forward flow, (2) the selector valves are set to the first injection channel position, and (3) 2 μL of the corresponding reagent are injected into the channel.*

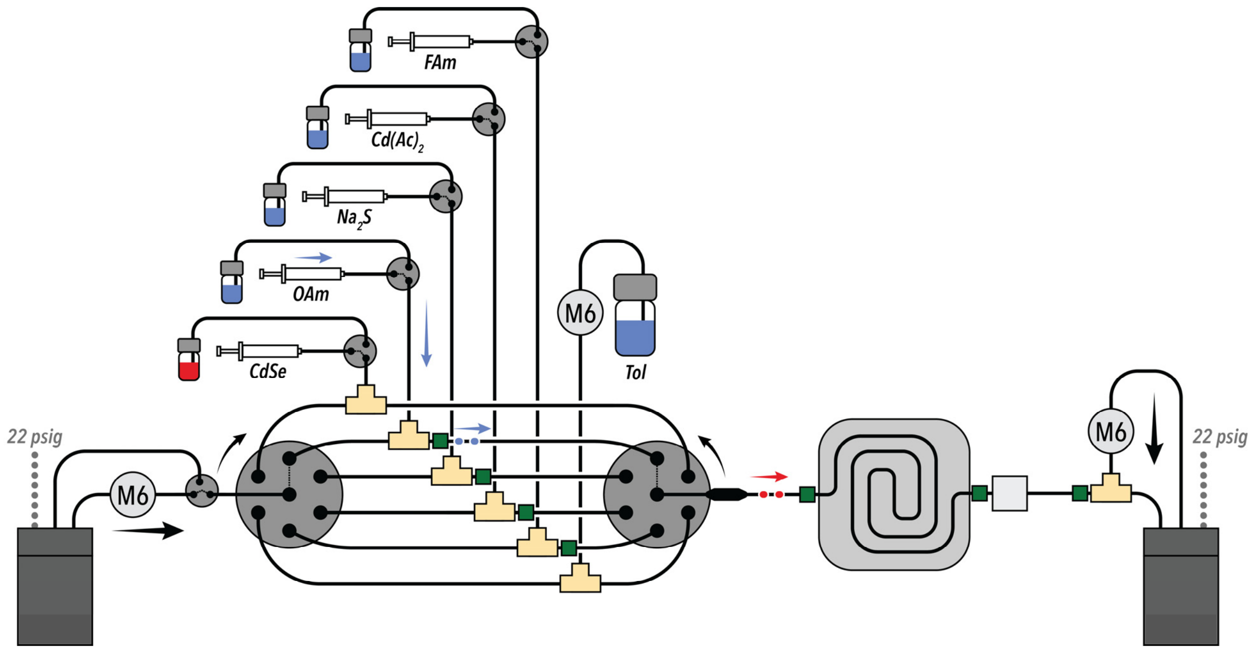

Supplementary Figure 8B – Waste and Cleaning Step 4. *(4) Steps 1-3 are repeated for each of the remaining four injection precursors.*

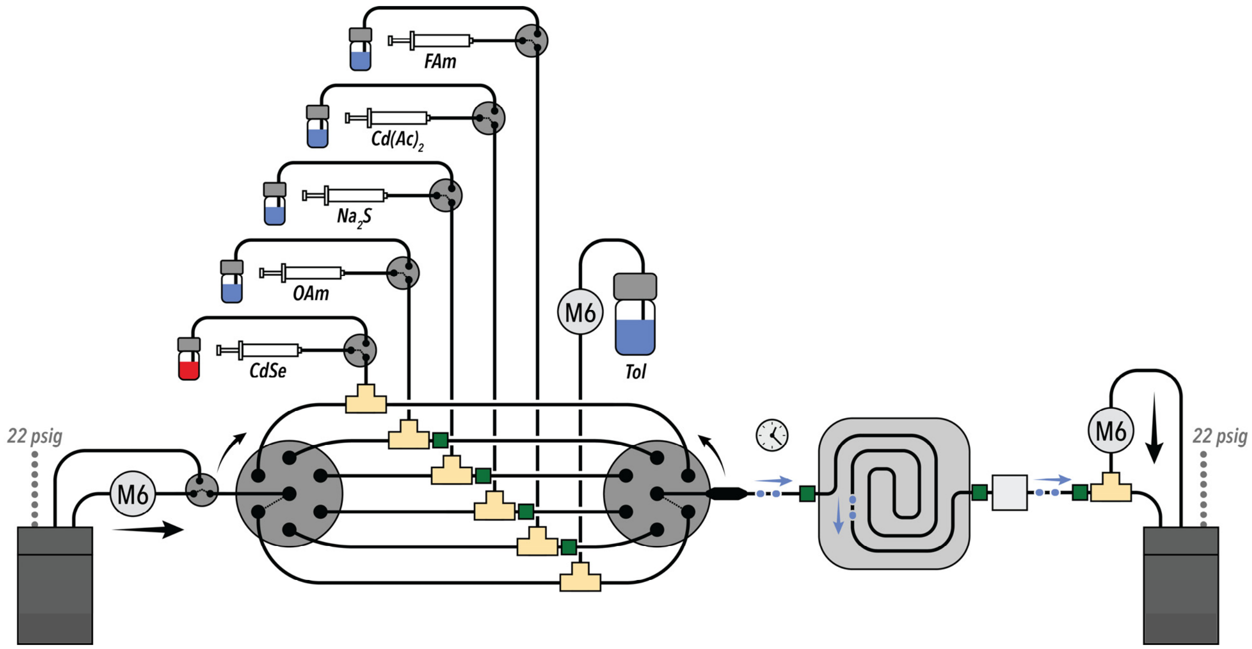

Supplementary Figure 8C – Waste and Cleaning Steps 5-6. *(5) The selector valve is set to the primary position, and (6) the carrier pumps continue to flow forward for 1 min.*

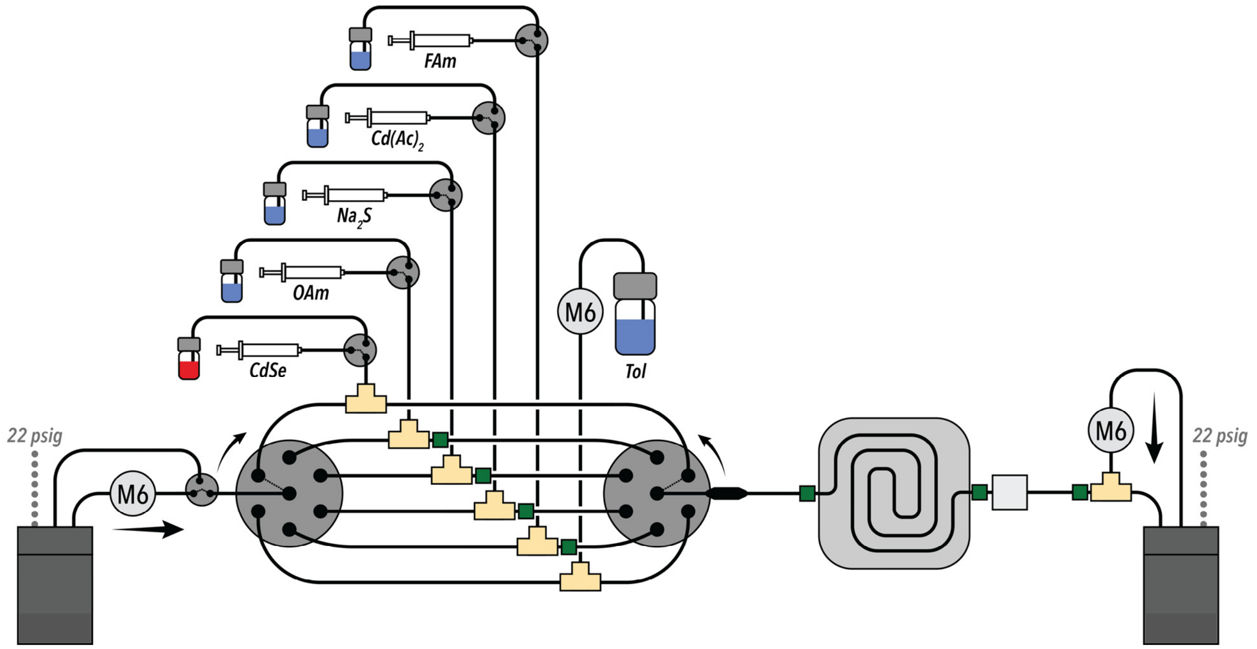

Supplementary Figure 8D – Waste and Cleaning Step 7. *After waiting 1 min, (7) the selector valves slowly increment through all positions. This step ensures that no smaller droplets are caught in the selector valve channels or fittings.*

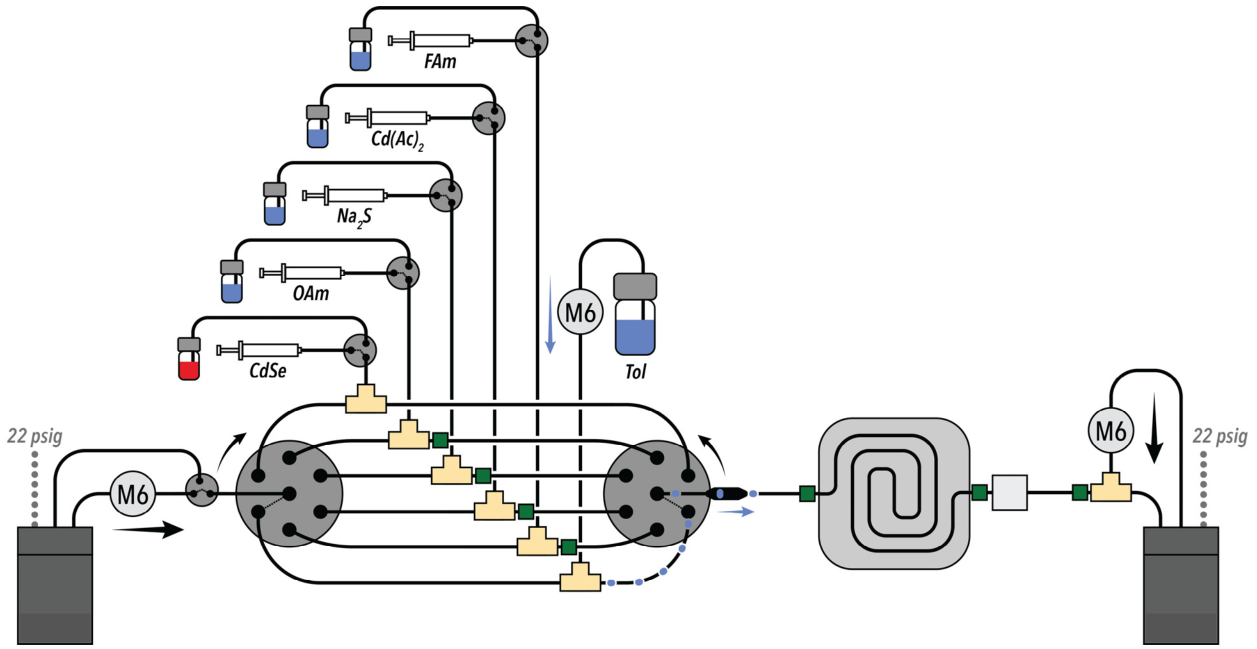

Supplementary Figure 8E – Waste and Cleaning Steps 8-10. *(8) After returning to the primary valve position, (9) 50 µL of toluene are injected into the reactor channel, (10) then both carrier pumps are stopped after all droplets have been removed from the reactor.*

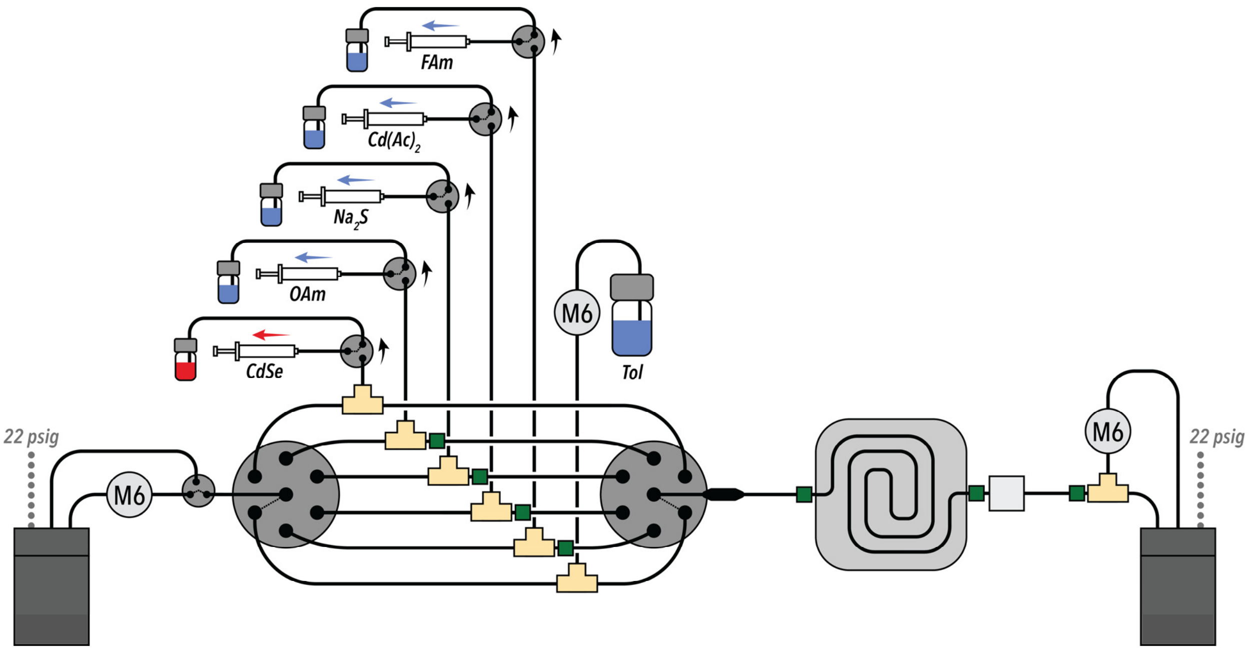

Supplementary Figure 9A – Syringe Refill Steps 1-2. *(1) All refill valves are switch to from the reactor to the source precursors, and (2) the syringes are set to withdraw until their full volumes are reached. The current syringe volumes are continuously tracked throughout experimentation by the reactor control software, so the withdraw volume in this stage is simply the current volume minus the maximum volume of each syringe.*

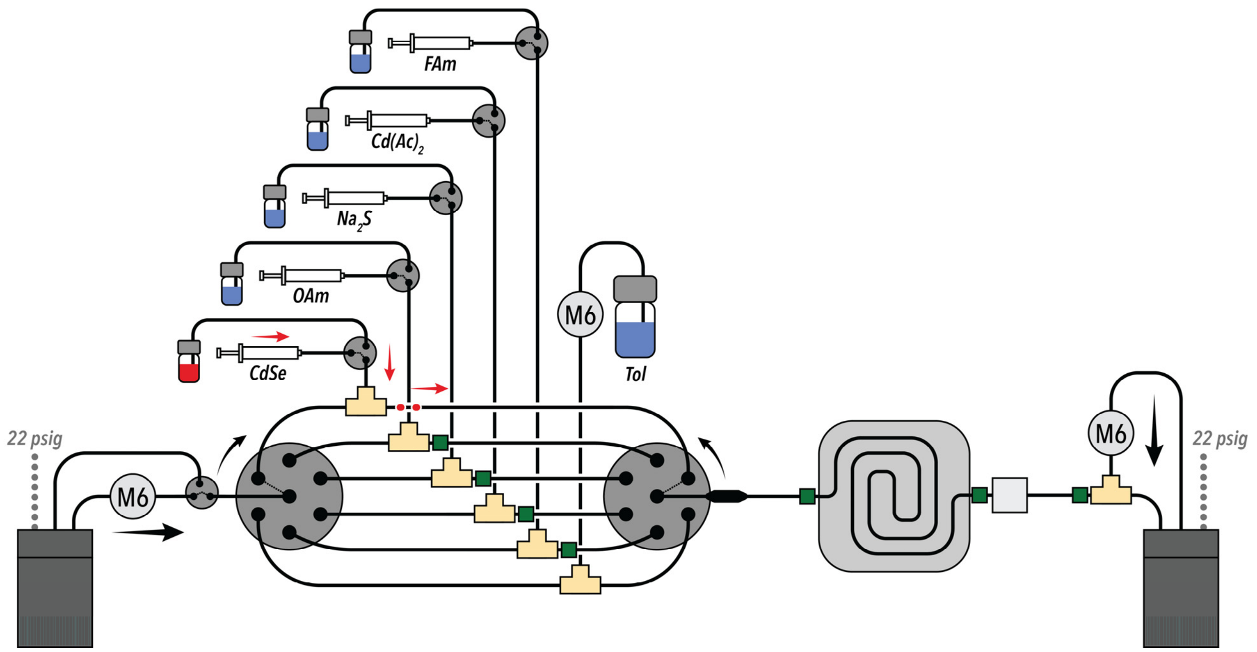

Supplementary Figure 9B – Syringe Refill Steps 3-6. *(3) The refill valves are returned to the reactor side position, (4) both carrier pumps are set to flow forward, (5) then the selector valves are set to the first precursor where (6) 50 µL are injected into the channel.*

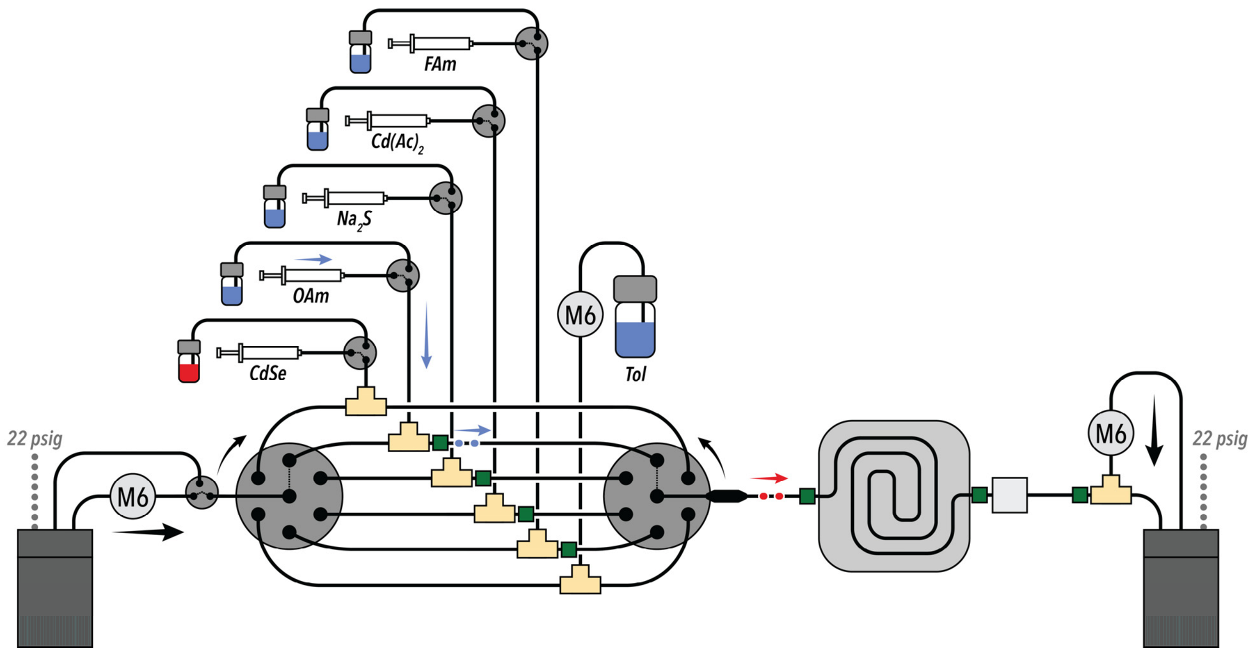

Supplementary Figure 9C – Syringe Refill Steps 7-9. *(7) Steps 5-6 are repeated for each of the refilled injecting precursors, (8) then all droplets are purged from the reactor, and (9) the selector valves are returned to their primary position.*

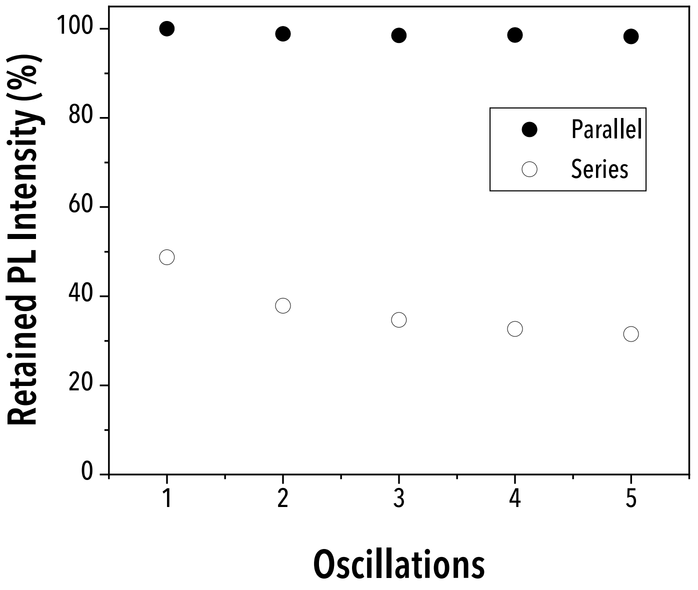

Supplementary Figure 10 – Parallel vs. Serial Injection System. *The percentage of the starting CdSe QD photoluminescence intensity retained as a function of oscillation number in the reactor segment for a parallel and serial injection configuration. Both studies featured reactor washing and line purging protocols for all reagents. In the serial injection design, the starting CdSe droplet passed through all reagent injection tees without deliberate reagent injection.*

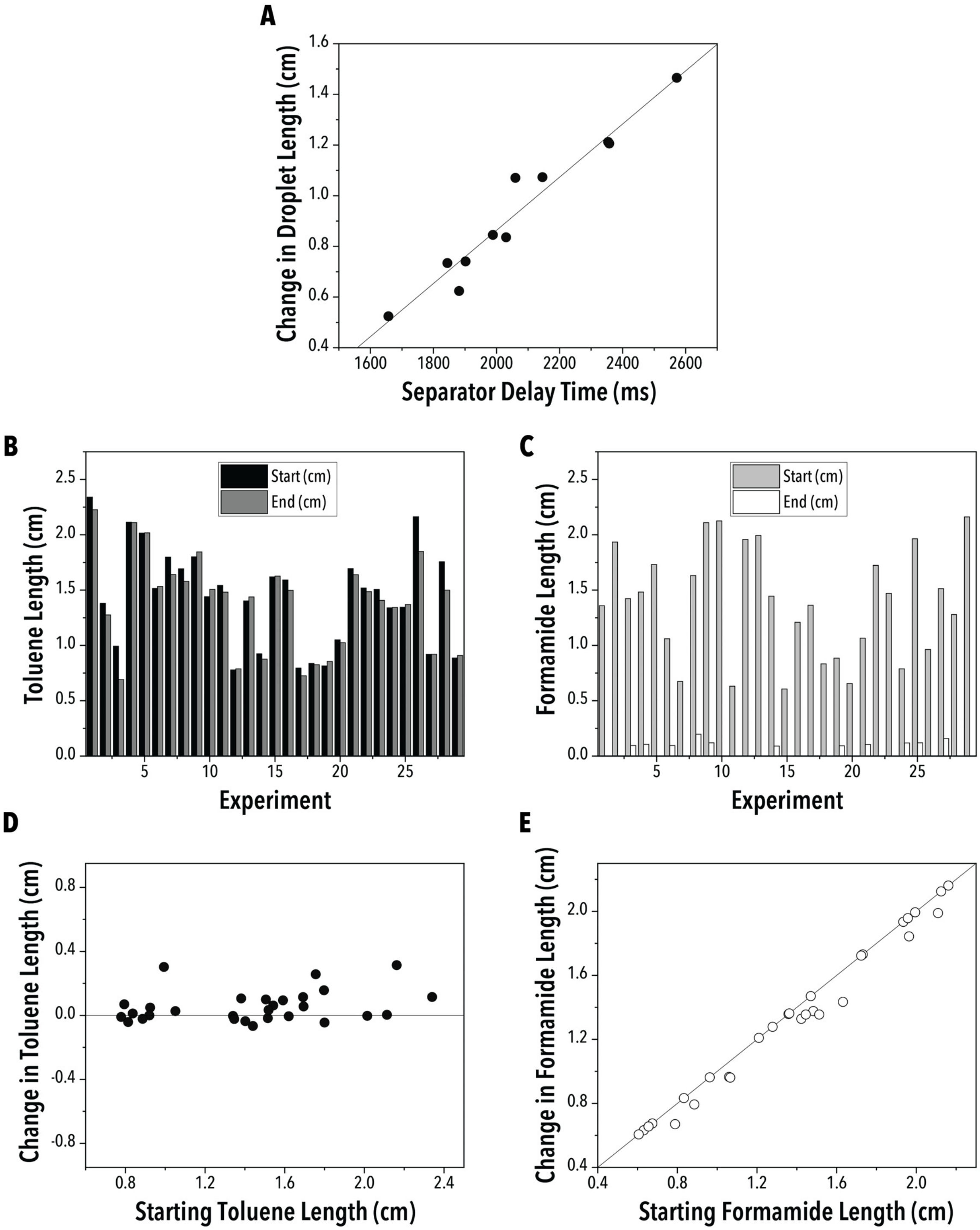

Supplementary Figure 11 – Phase Separator Validation. *(A) Measured change in droplet length as a function of the separator delay time for ten 6 µL toluene and 6 µL formamide droplets with a linear fit. (B) The measured toluene and (C) formamide phase lengths before and after droplet separation for a collection of random phase volume droplets and (D) the change in toluene and (E) formamide phase length as a function of the starting droplet size.*

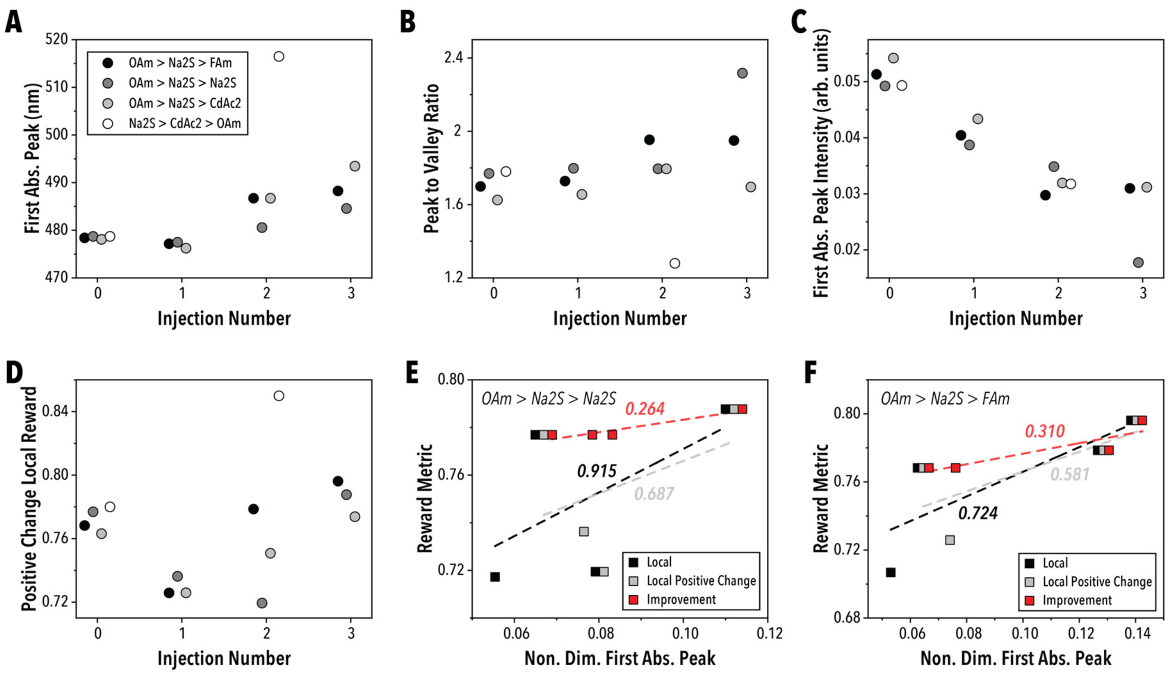

Supplementary Figure 12 – Illustration of reward function variants. *(A) First absorption peak wavelength, (B) absorption peak to valley ratio, (C) first absorption peak intensity, and (D) weighted mean reward calculated using positive increases in the first absorption peak for four different sample injection sequences. Slope reward calculation methods for the slope of the weighted mean reward, weighted mean positive change reward, and improvement reward for (E) CdSe > OAm > Na_2S > Na_2S and (F) CdSe > OAm > Na_2S > FAm. Non. Dim. First Abs. Peak: Non-dimensionalized first absorption peak wavelength.*

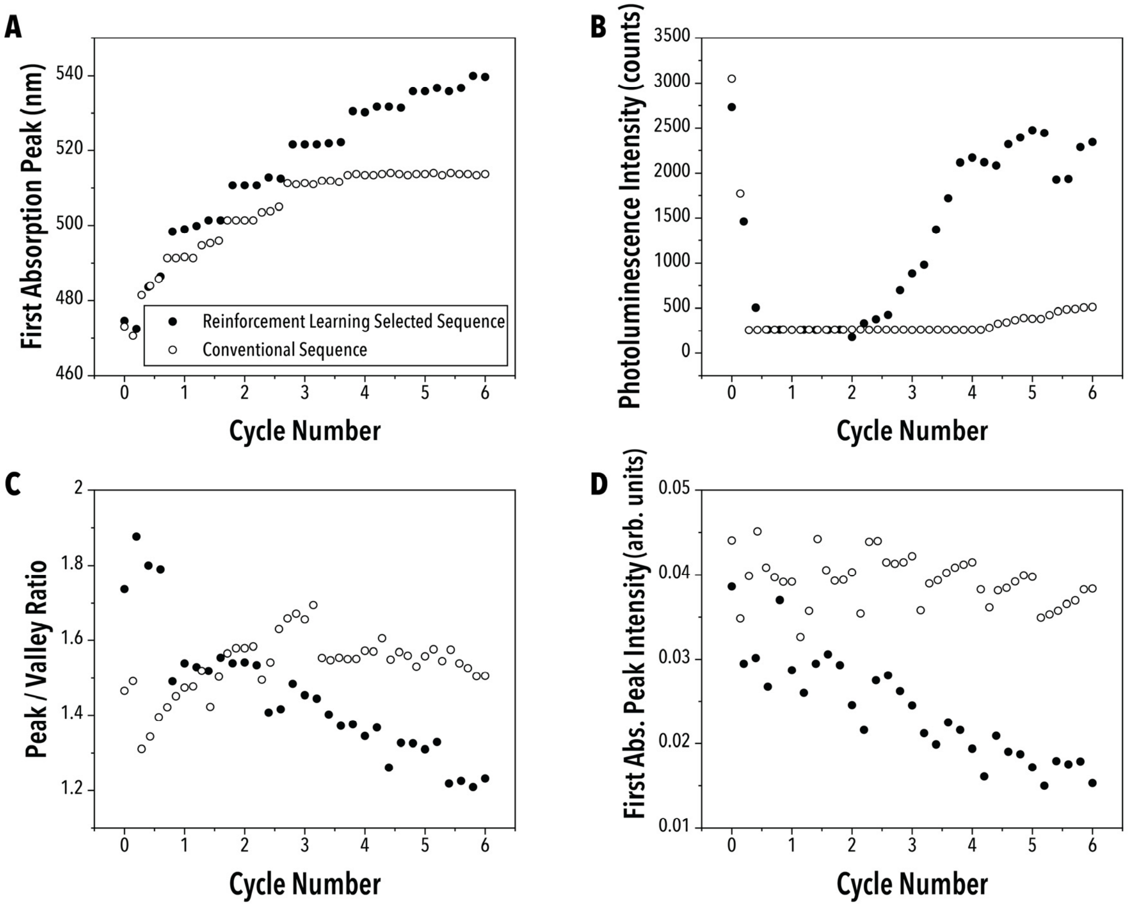

Supplementary Figure 13 – Comparison of RL-selected and conventional cALD injection sequences. *In-situ obtained (A) first absorption peak wavelength, (B) photoluminescence peak intensity, (C) peak to valley ratio, and (D) first absorption peak intensity for the RL-selected sequence and conventional sequence of cALD chemistry. Data is taken from the sequence selection campaign using the 480 nm starting CdSe.*

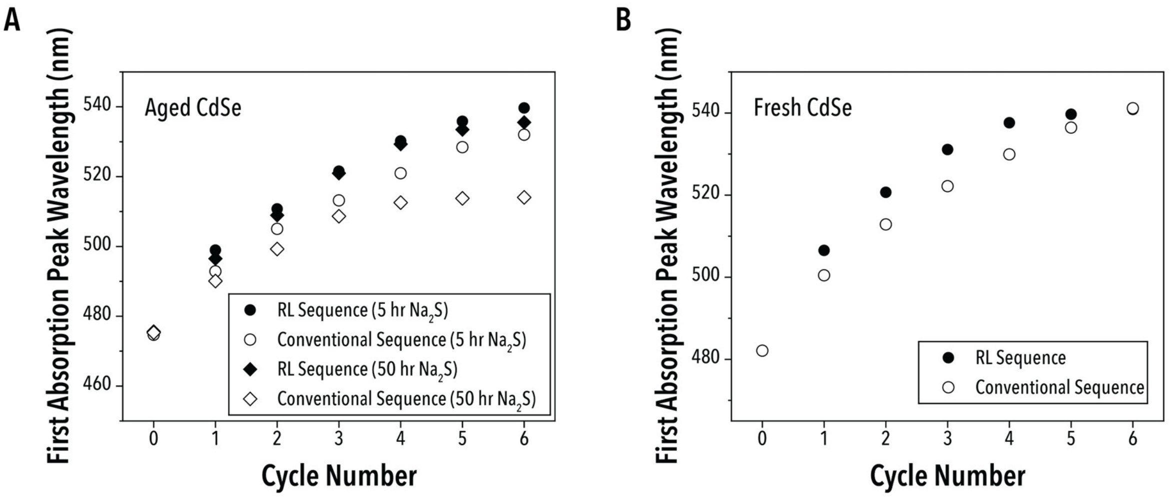

Supplementary Figure 14 – Cycling with Aged and Fresh CdSe. *First absorption peak wavelength as a function of cycle number for the RL selected injection sequence and conventional injection sequence with (A) CdSe diluted 20 days before use (with corresponding sodium sulfide age shown in the legend) and (B) diluted within 2 days of use.*

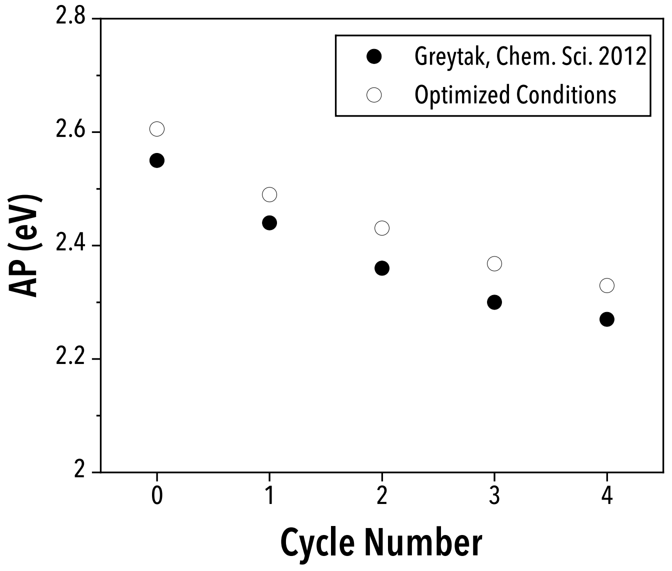

Supplementary Figure 15 – Optimized Results vs. SILAR. *First absorption peak as a function of cycle number for the optimized 480 nm volume and time exploitation and literature data on SILAR from Greytak et al., Chem. Sci., 2012, 3, 2028-2034.*

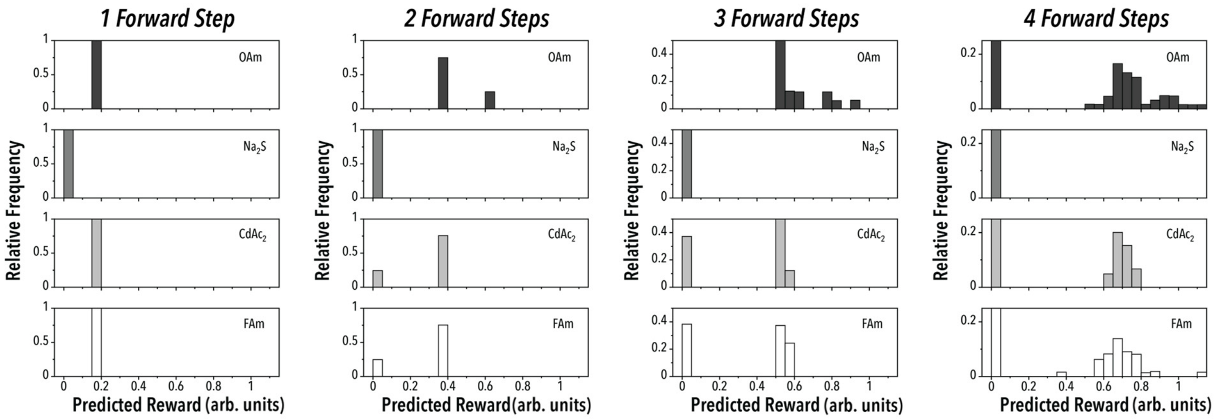

Supplementary Figure 16 – Sequence Selection Forward Mapping Levels. *Frequency histograms of the forward predicted reward at four levels of forward prediction for the four reagent injection options in the cALD chemistry exploration campaigns, starting from the initial QD.*

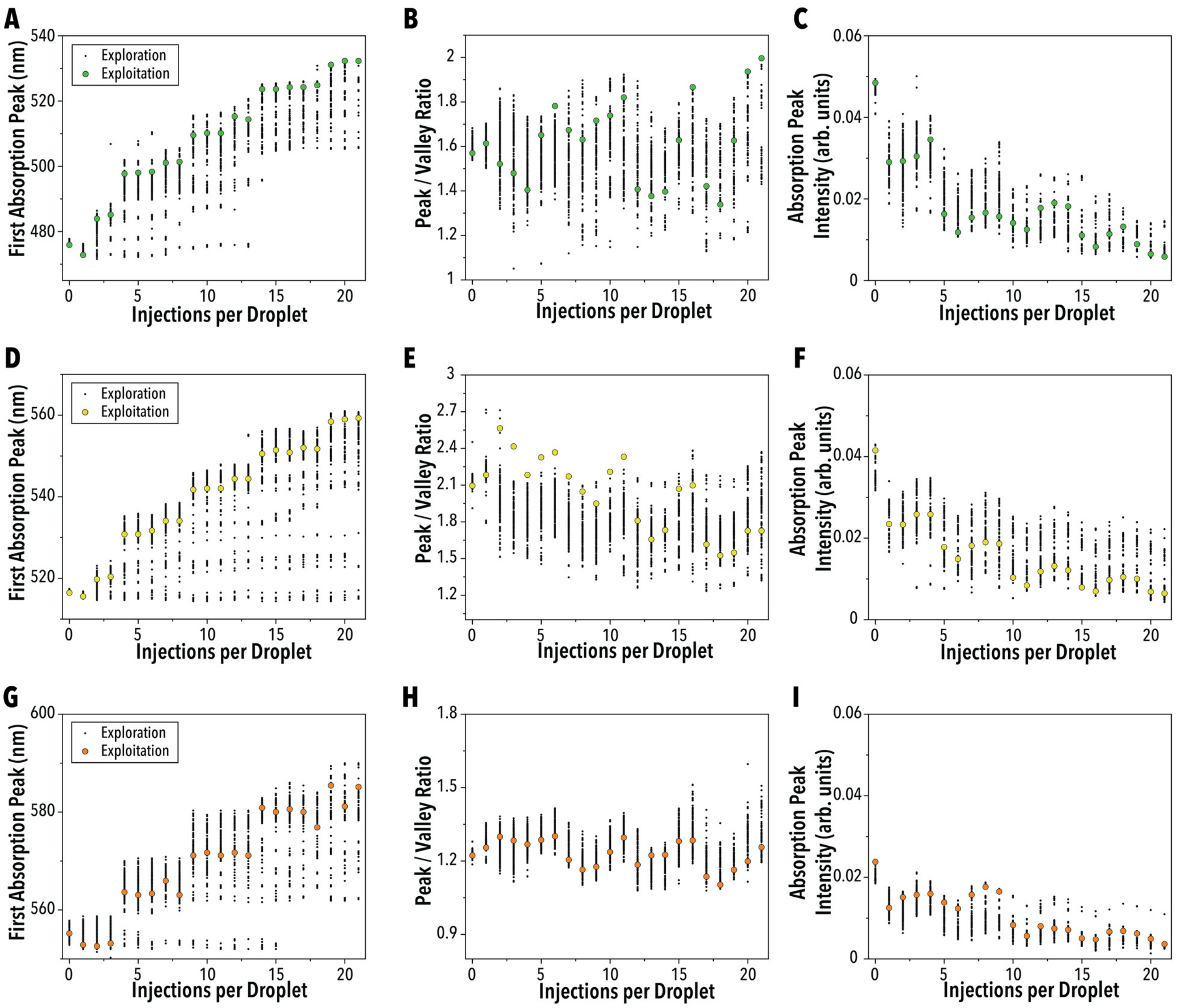

Supplementary Figure 17 – Complete Volume and Time Optimization Data. *First absorption peak wavelength, peak to valley ratio, and absorption peak intensity as a function of the total number of precursor injections per droplet for all exploration and exploitation experiments of the volume and time optimization campaigns with QD starting wavelength of (A, B, C) 480 nm, (D, E, F) 520 nm, and (G, H, I) 560 nm.*

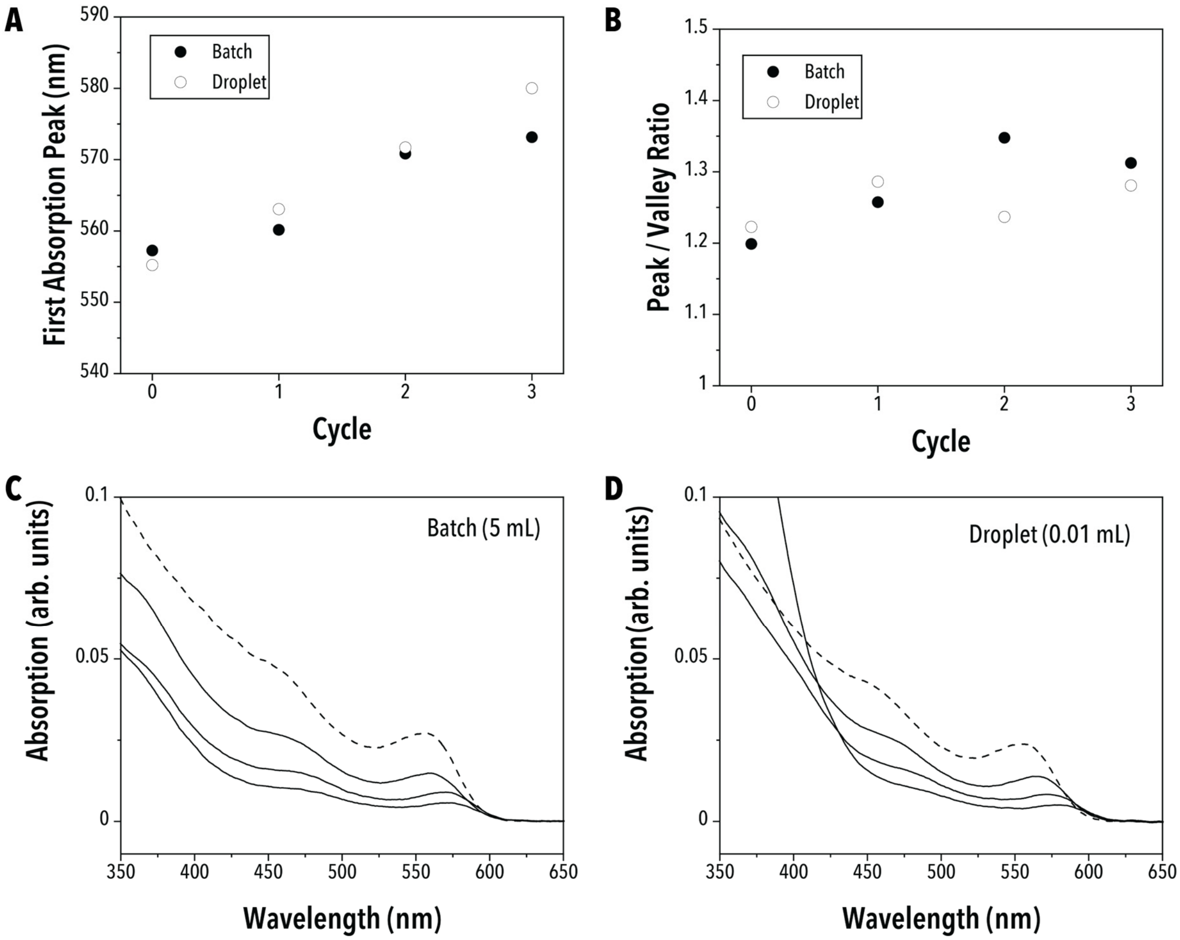

Supplementary Figure 18 – Batch Replication of Optimized Conditions. *(A) First absorption peak and (B) peak to valley ratio as a function of cycle number for the optimized conditions in the droplet reactor and the same conditions replicated in batch with (C and D) corresponding absorption spectra. The starting QDs are shown with a dashed line. Batch experiments were conducted at 500x scale up.*

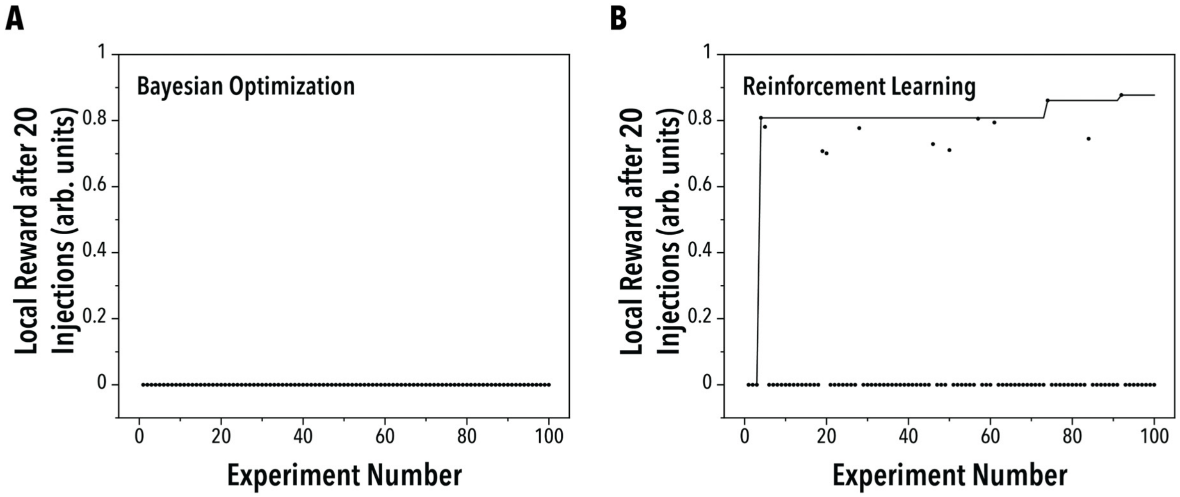

Supplementary Figure 19 – Bayesian Optimization vs. Reinforcement Learning with Digital Twin. *The local reward after 20 injections as a function of experiment number (i.e., one complete droplet) for optimization campaigns run on the digital twin with (A) Bayesian optimization and (B) reinforcement learning. Terminal conditions are given a reward of zero.*

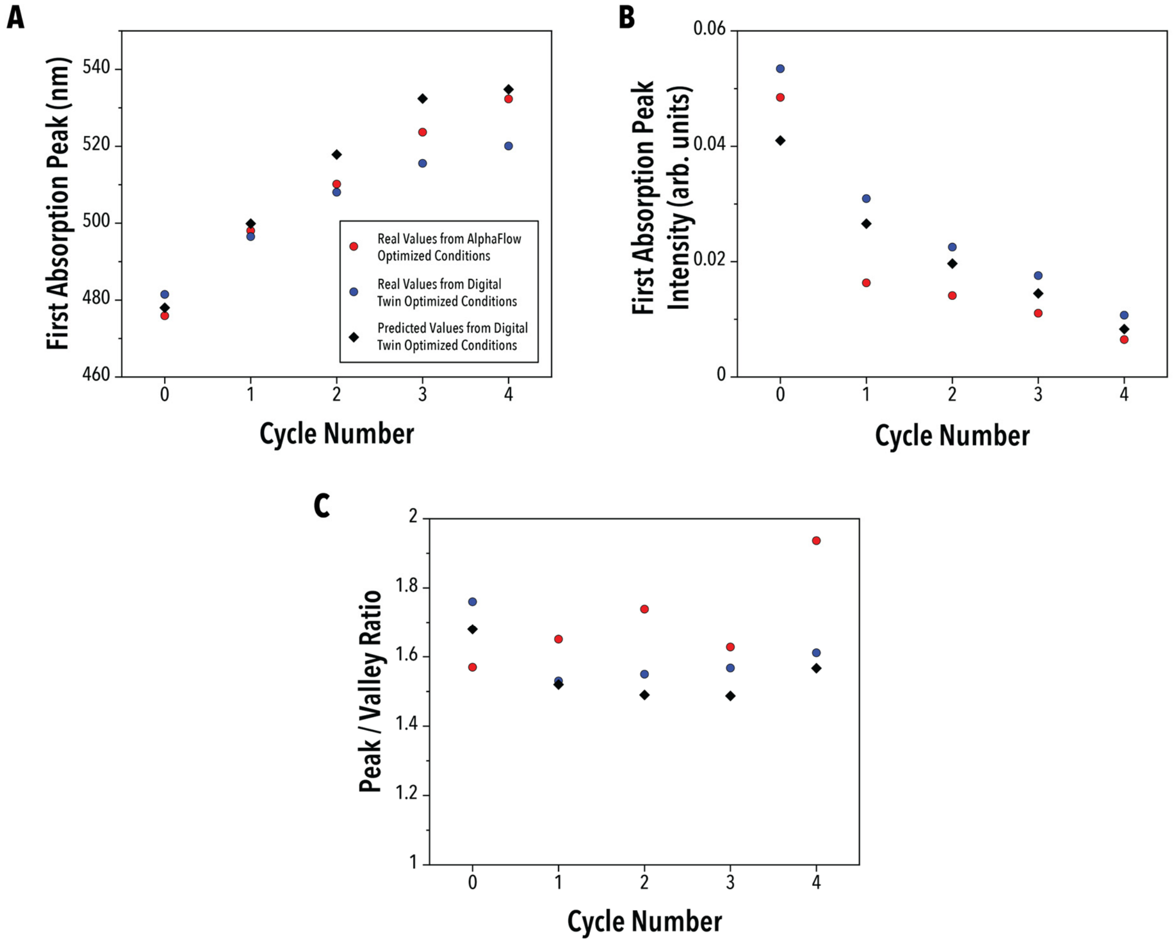

Supplementary Figure 20 – Measured Values for Digital Twin Sequence. *(A) The first absorption peak wavelength, (B) first absorption peak intensity, and (C) peak to valley ratio for the AlphaFlow optimized volume and time conditions, the digital twin optimized volume and time conditions, and the outputs predicted by the digital twin with the same optimized conditions.*

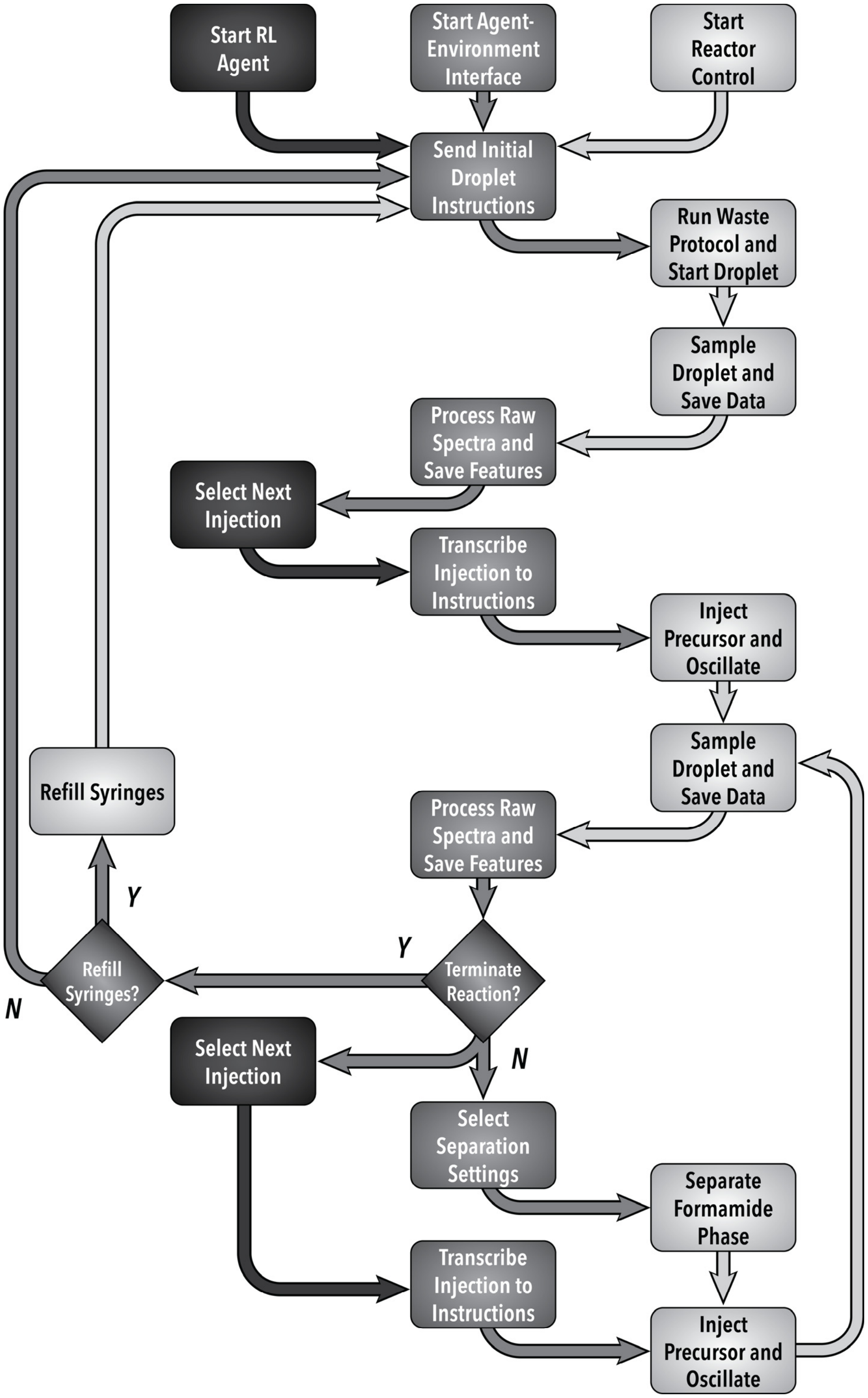

Supplementary Figure 21 – Full System Operation Flow Chart.

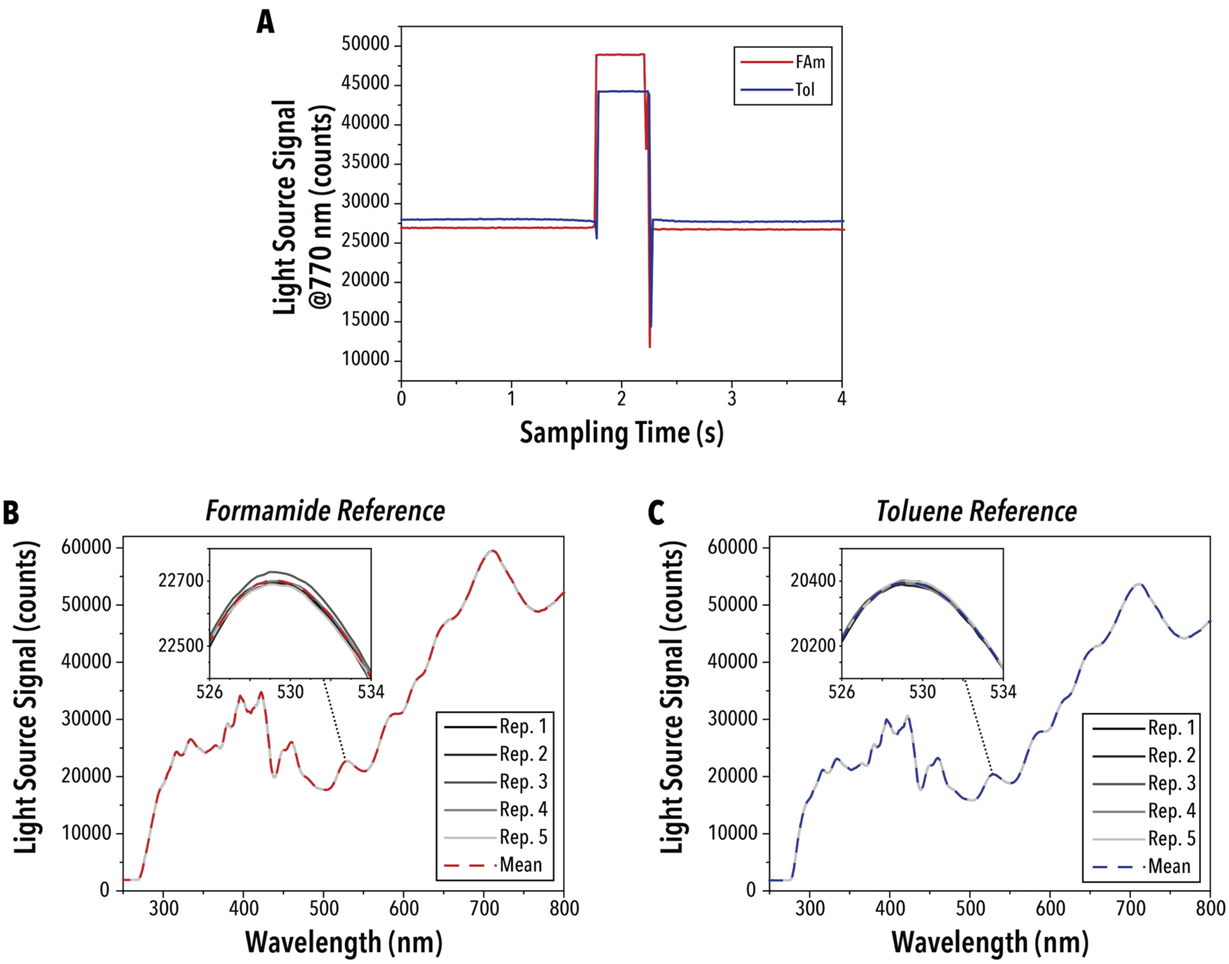

Supplementary Figure 22 – Absorption Light Reference Collection. *(A) Light source signal intensity as a function of the sampling time for the formamide and toluene reference droplets. Extracted light reference spectra for (B) formamide and (C) toluene reference samples with corresponding replicates used for the final reference spectra.*

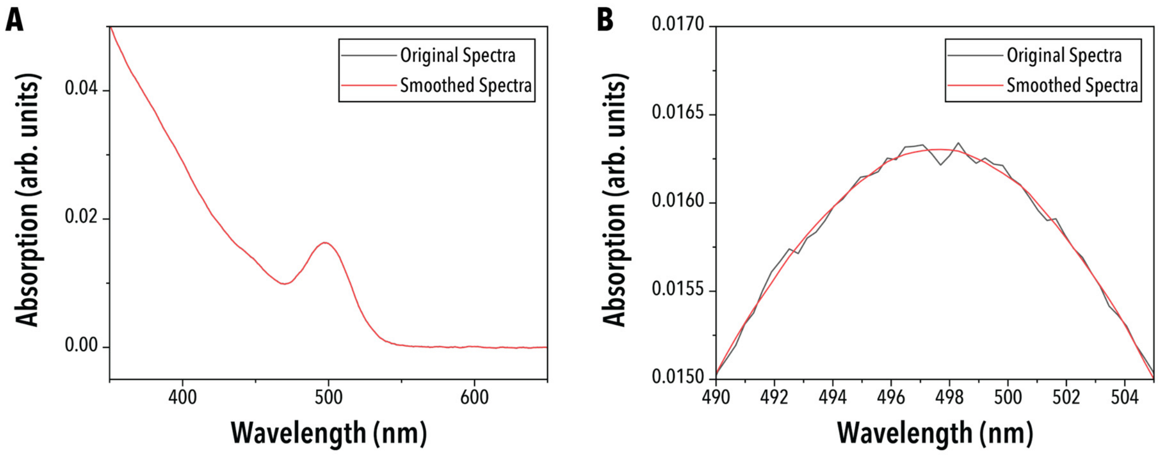

Supplementary Figure 23 – UV-Vis Absorption Spectra Pre-Processing. *(A) Sample absorption spectra with and without Savitzky-Golay filtering with (B) a close zoom of the absorption peak.*

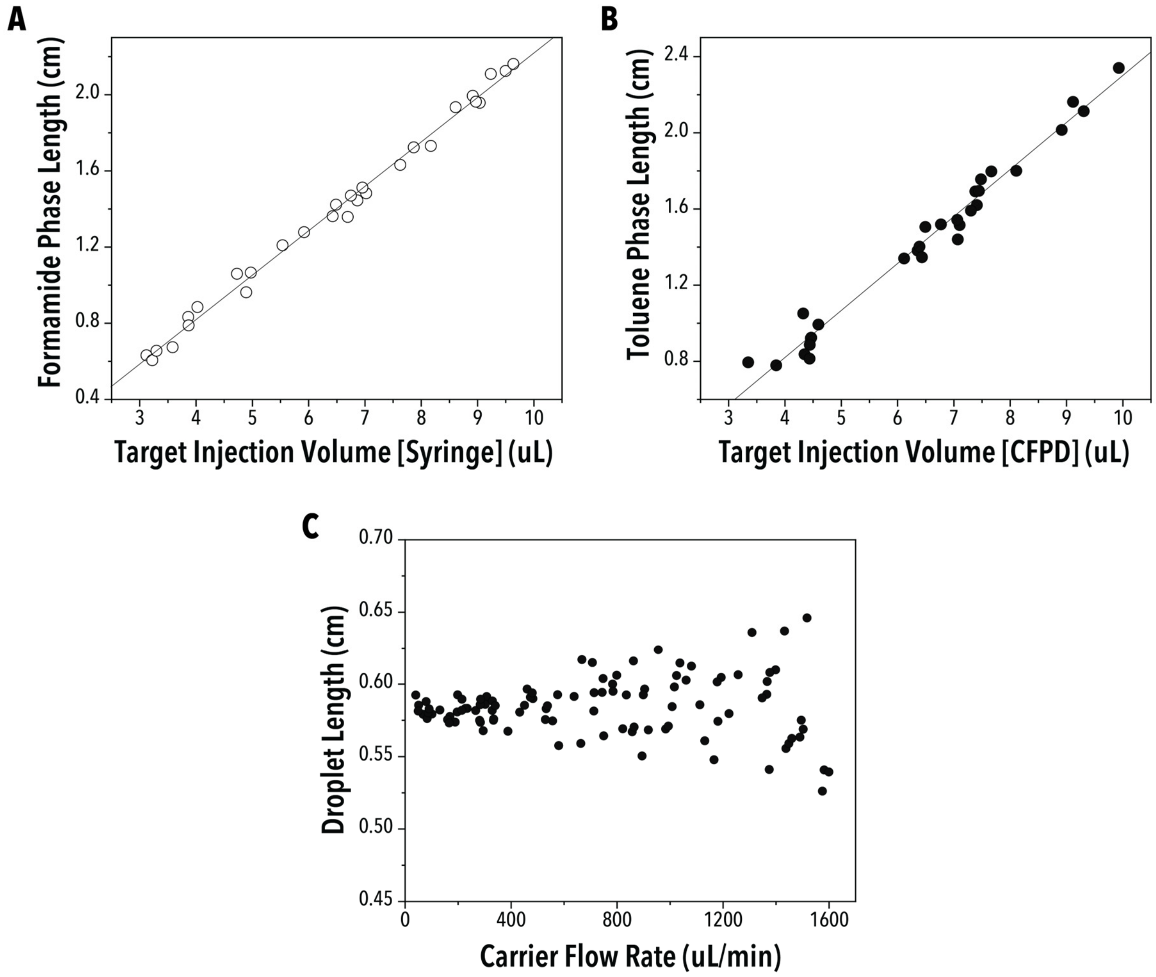

Supplementary Figure 24 – Droplet Phase Length Measurement Validation. *Measured (A) formamide and (B) toluene droplet phase lengths as a function of the set injection volume using a Harvard apparatus syringe pump and VICI M6 continuous flow positive displacement pump respectively for 29 combined droplets, and (C) the measured droplet length of a single 3 µL toluene droplet as a function of the carrier flow rate.*

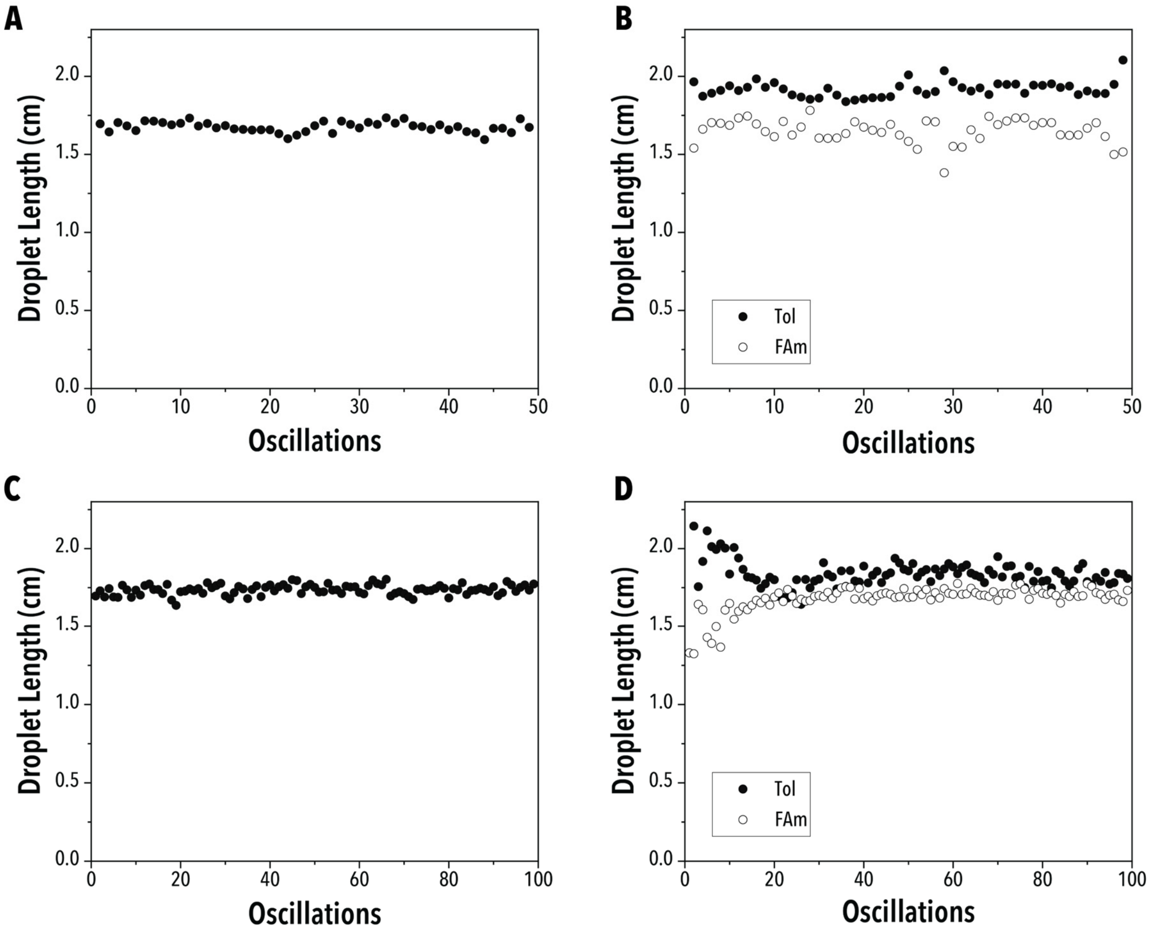

Supplementary Figure 25 – Phase Retention in Mobile Droplets. *Measured droplet phase length for (A) a single toluene droplet and (B) a biphasic toluene and formamide droplet over 50 oscillations in the reactor spiral and 100 oscillations through both the selector valve and reactor spiral (C and D respectively).*

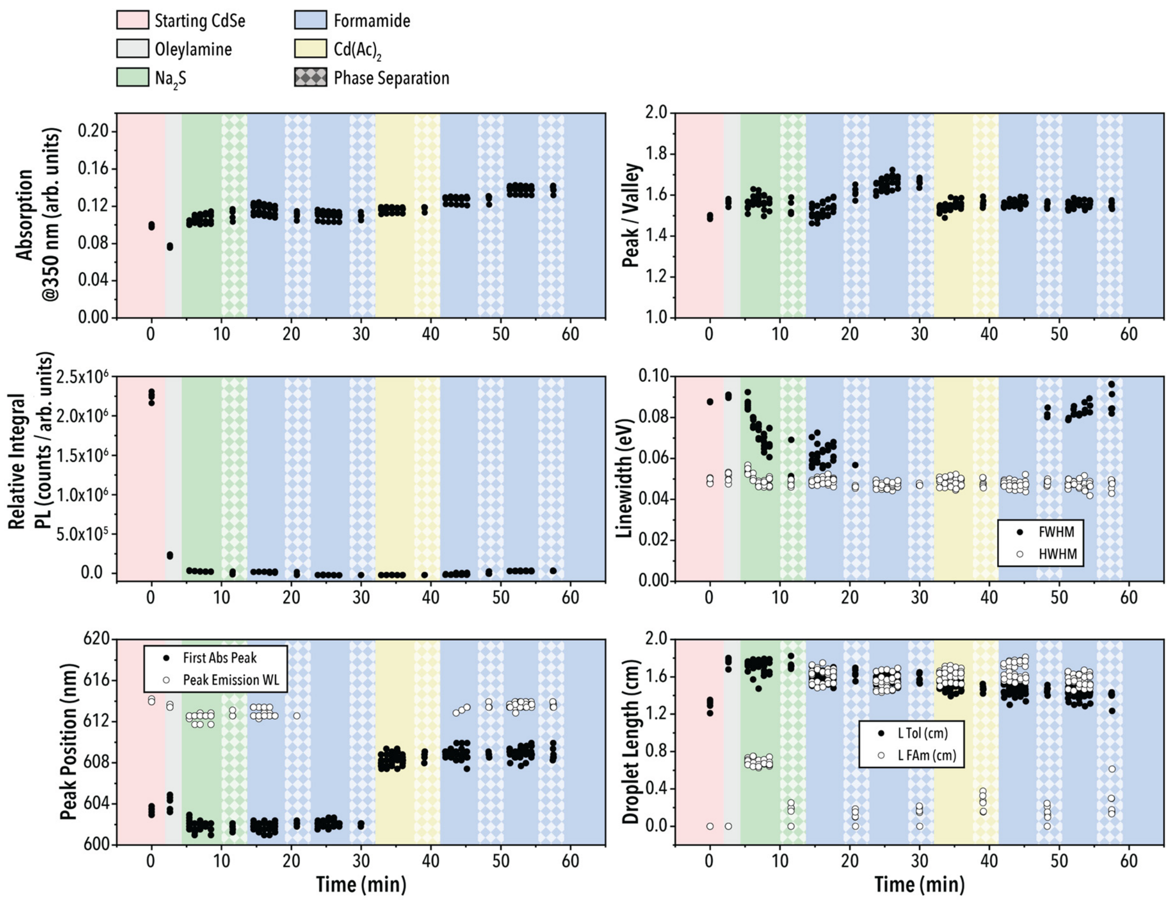

Supplementary Figure 26 – Conventional Cycle Reproducibility. *Absorption intensity, peak to valley ratio, relative integral photoluminescence intensity, emission and absorption linewidths, emission and absorption peak wavelength, and measured droplet phase lengths as a function of experiment time for five replicates of single conventional cycle sequences. The plot background color corresponds to the most recent precursor injection, and the background pattern corresponds to (solid) droplet oscillation and (diamond) phase separation.*

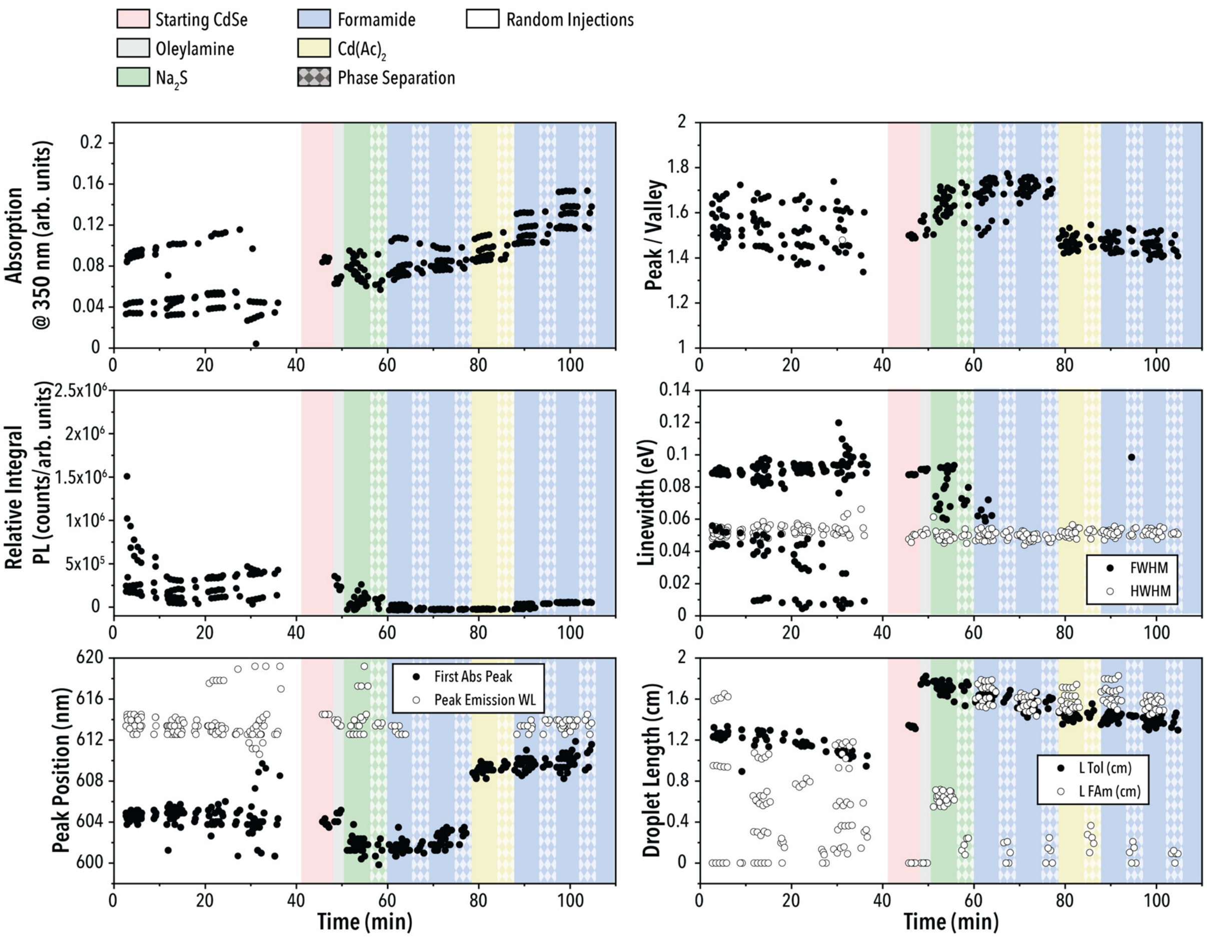

Supplementary Figure 27 – Reaction Conduction Independence. *Conventional Cycle Reproducibility***.** *Absorption intensity, peak to valley ratio, relative integral photoluminescence intensity, emission and absorption linewidths, emission and absorption peak wavelength, and measured droplet phase lengths as a function of experiment time for five replicates of single conventional cycle sequences, each preceded by three random injection conditions and the reactor washing protocol. The plot background color corresponds to the most recent precursor injection, and the background pattern corresponds to (solid) droplet oscillation and (diamond) phase separation.*

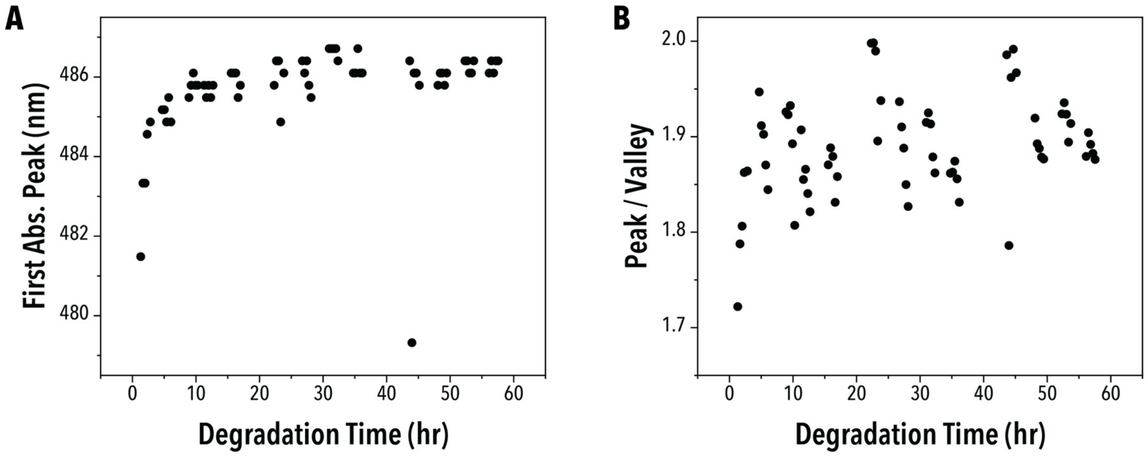

Supplementary Figure 28 – Sodium Sulfide Operational Window. *(A) First absorption peak wavelength and (B) peak to valley ratio after the conventional have cycle for 65 consecutive replicates, starting with a fresh sodium sulfide precursor.*

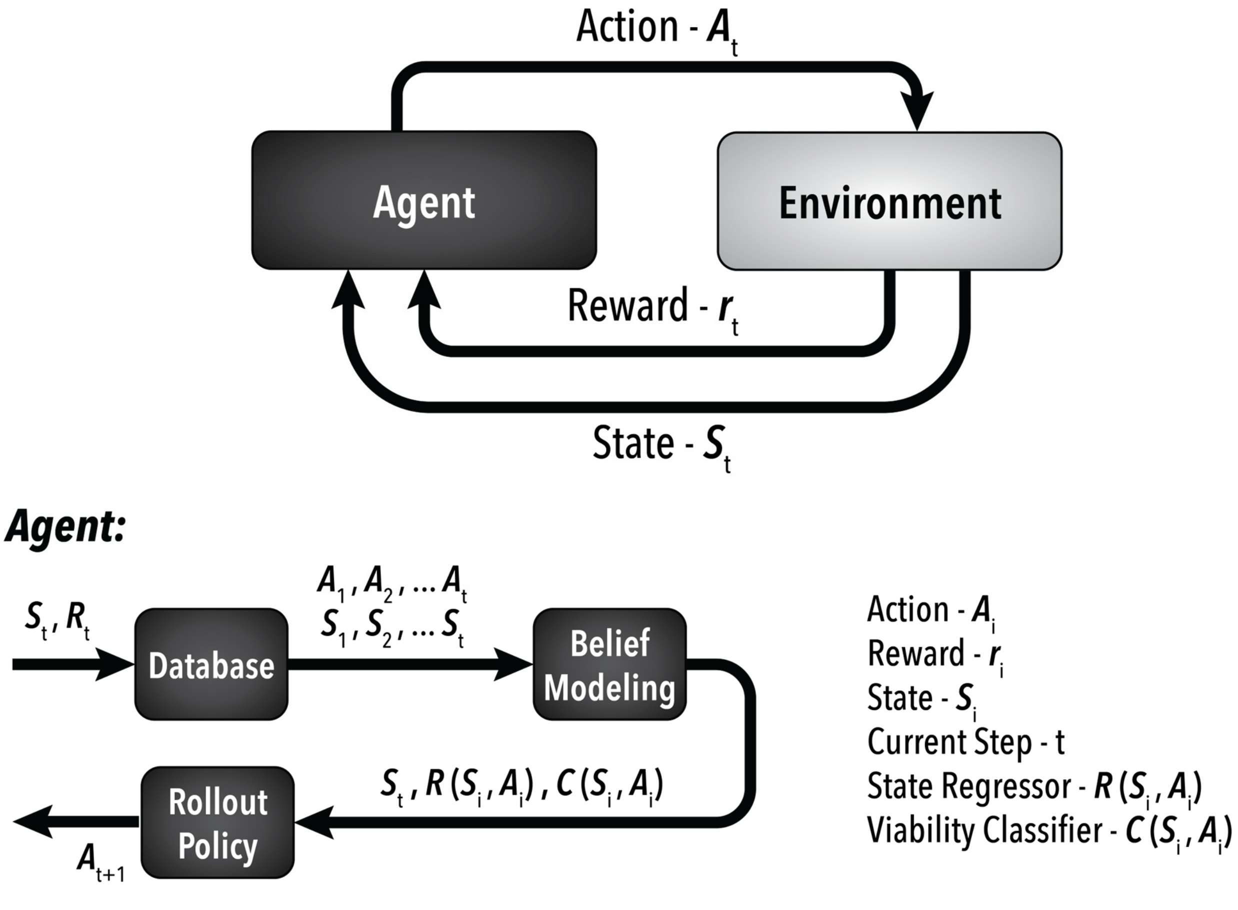

Supplementary Figure 29 – General Structure of Reinforcement Learning Algorithm. *Process flow diagram of the over algorithm structure employed in AlphaFlow studies.*

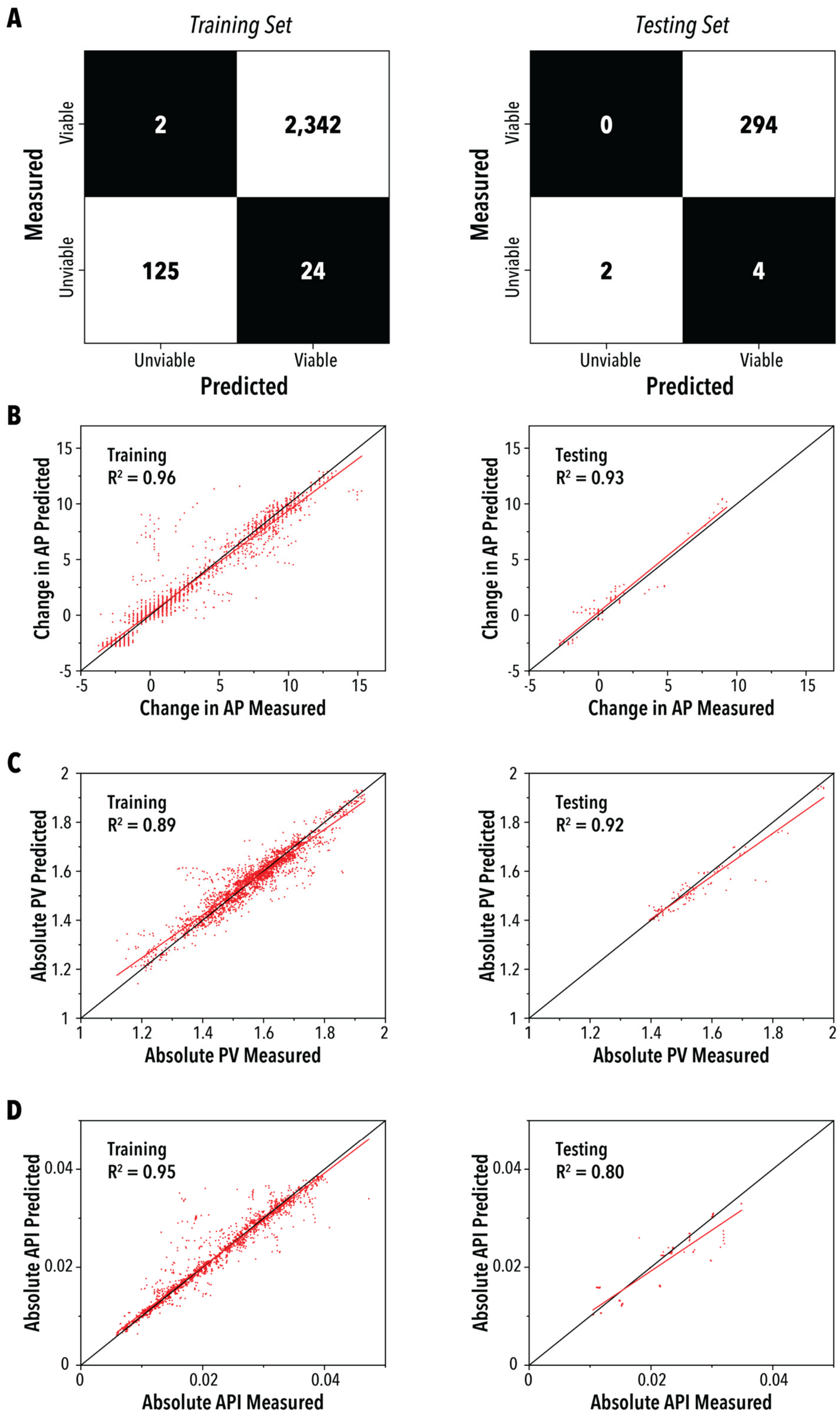

Supplementary Figure 30 – Digital Twin Training-Testing Regressions. *Training and testing data set regressions for the (A) viability classifier, (B) first absorption peak regressor, (C) peak to valley ratio regressor, and (D) first absorption peak intensity regressor used on the digital twin.*

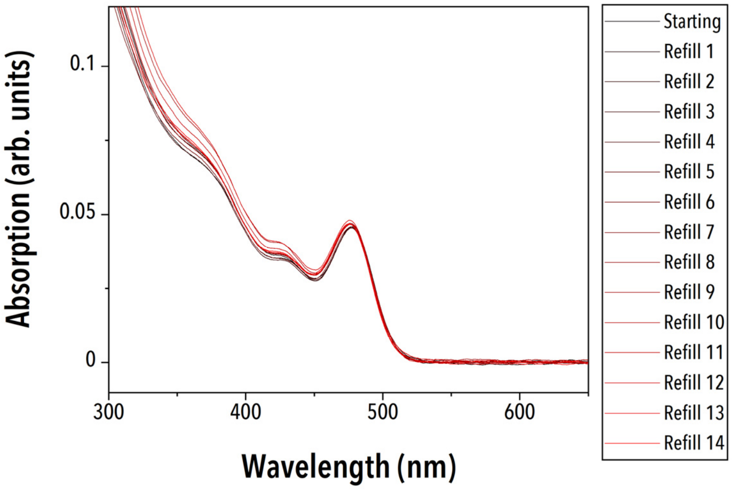

Supplementary Figure 31 – CdSe absorption spectra after the syringe refill protocol. *In-situ obtained UV-Vis absorption spectra of the starting CdSe quantum dot solution acquired using 10 µL droplets immediately after running the syringe refill protocols. The data was taken from all fourteen refills conducted in the 480 nm volume and time optimization campaigns, and the standard deviation of the absorption at 365 nm across all refills is 0.003.*

Supplementary References

1. Saputro, D. R. S. & Widyaningsih, P. Limited memory Broyden-Fletcher-Goldfarb-Shanno (L-BFGS) method for the parameter estimation on geographically weighted ordinal logistic regression model (GWOLR). *AIP Conf. Proc.* **1868**, 040009 (2017).

2. Epps, R. W. *et al.* Artificial Chemist: An Autonomous Quantum Dot Synthesis Bot. *Adv. Mater.* **32**, 2001626 (2020).
