# Supplementary material for: AlphaFlow: autonomous discovery and optimization of multi-step chemistry using a self-driven fluidic lab guided by reinforcement learning
Source: Nat Commun. 2023 Mar 14;14:1403. doi: 10.1038/s41467-023-37139-y (PMC10015005; doi:10.1038/s41467-023-37139-y)
Supplement: Supplementary file 2 — Description of Additional Supplementary Files [file 41467_2023_37139_MOESM2_ESM.pdf]

File name: Supplementary Movie 1

Description: A video of different AlphaFlow modules' operation
